# A Genome-First Approach to Characterize *DICER1* Pathogenic Variant Prevalence, Penetrance, and Phenotype

**DOI:** 10.1001/jamanetworkopen.2021.0112

**Published:** 2021-02-25

**Authors:** Uyenlinh L. Mirshahi, Jung Kim, Ana F. Best, Zongming E. Chen, Ying Hu, Jeremy S. Haley, Alicia Golden, Richard Stahl, Kandamurugu Manickam, Ann G. Carr, Laura A. Harney, Amanda Field, Jessica Hatton, Kris Ann P. Schultz, Andrew J. Bauer, D. Ashley Hill, Philip S. Rosenberg, Michael F. Murray, David J. Carey, Douglas R. Stewart

**Affiliations:** 1 Geisinger Clinic, Geisinger Health System, Danville, Pennsylvania; 2 Clinical Genetics Branch, Division of Cancer Epidemiology and Genetics, National Cancer Institute, Rockville, Maryland; 3 Biostatistics Branch, Biometric Research Program, Division of Cancer Treatment and Diagnosis, National Cancer Institute, National Institutes of Health, Rockville, Maryland; 4 Department of Laboratory Medicine and Pathology, Mayo Clinic, Rochester, Minnesota; 5 Department of Endocrinology, Main Line Health System, Wynnewood, Pennsylvania; 6 Division of Genetic and Genomic Medicine, Nationwide Children’s Hospital, Columbus, Ohio; 7 Weststat, Inc, Rockville, Maryland; 8 ResourcePath, Sterling, Virginia; 9 Cancer and Blood Disorders, Children’s Minnesota, Minneapolis; 10 International Pleuropulmonary Blastoma/*DICER1* Registry, Minneapolis, Minnesota; 11 International Ovarian and Testicular Stromal Tumor Registry, Minneapolis, Minnesota; 12 The Thyroid Center, Division of Endocrinology and Diabetes, Children’s Hospital of Philadelphia, Philadelphia, Pennsylvania; 13 Division of Pathology and Center for Cancer and Immunology Research, Children's National Health System, Washington, DC; 14 Department of Integrative Systems Biology, George Washington University School of Medicine and Health Sciences, Washington, DC; 15 Department of Genetics, Yale School of Medicine, New Haven, Connecticut

## Abstract

**Importance:**

Genetic disorders are historically defined through phenotype-first approaches. However, risk estimates derived from phenotype-linked ascertainment may overestimate severity and penetrance. Pathogenic variants in *DICER1* are associated with increased risks of rare and common neoplasms and thyroid disease in adults and children. This study explored how effectively a genome-first approach could characterize the clinical traits associated with germline *DICER1* putative loss-of-function (pLOF) variants in an unselected clinical cohort.

**Objective:**

To examine the prevalence, penetrance, and phenotypic characteristics of carriers of germline *DICER1* pLOF variants via genome-first ascertainment.

**Design, Setting, and Participants:**

This cohort study classifies *DICER1* variants in germline exome sequence data from 92 296 participants of the Geisinger MyCode Community Health Initiative. Data for each MyCode participant were used from the start of the Geisinger electronic health record to February 1, 2018.

**Main Outcomes and Measures:**

Prevalence of germline *DICER1* variation; penetrance of malignant tumors and thyroid disease in carriers of germline *DICER1* variation; structured, manual review of electronic health records; and *DICER1* sequencing of available tumors from an associated cancer registry.

**Results:**

A total of 92 296 adults (mean [SD] age, 59 [18] years; 98% white; 60% female) participated in the study. Germline *DICER1* pLOF variants were observed in 1 in 3700 to 1 in 4600 participants, more than double the expected prevalence. Malignant tumors (primarily thyroid carcinoma) were observed in 4 of 25 participants (16%) with *DICER1* pLOF variants, which is comparable (by 50 years of age) to the frequency of neoplasms in the largest registry- and clinic-based (phenotype-first) *DICER1* studies published to date. *DICER1* pLOF variants were significantly associated with risks of thyroidectomy (odds ratio [OR], 6.0; 95% CI, 2.2-16.3; *P* = .007) and thyroid cancer (OR, 9.2; 95% CI, 2.1-34.7; *P* = .02) compared with controls, but there was not a significant increase in the risk of goiter (OR, 1.8; 95% CI, 0.7-4.9). A female patient in her 80s who was a carrier of a germline *DICER1* hotspot variant was apparently healthy on electronic health record review. The term *DICER1* did not appear in any of the medical records of the 25 participants with a pLOF *DICER1* variant, even in those affected with a known *DICER1*-associated tumor or thyroid phenotype.

**Conclusions and Relevance:**

This cohort study was able to ascertain individuals with germline *DICER1* variants based on a genome-first approach rather than through a previously established *DICER1*-related phenotype. Use of the genome-first approach may complement more traditional approaches to syndrome delineation and may be an efficient approach for risk estimation.

## Key Points

**Question:**

What are the prevalence, risk, and phenotypic spectrum of individuals with a germline putative loss-of-function (pLOF) variant in *DICER1* according to a genome-first approach in a population-scale cohort?

**Findings:**

In this cohort study, *DICER1* pLOF variants were more than twice as common (even after adjustment for relatedness) than previously observed. Malignant tumors were observed in 16% of participants with a *DICER1* pLOF variant, which is comparable to the frequency of neoplasms in the largest phenotype-first *DICER1* studies published to date.

**Meaning:**

The genome-first approach complements more traditional approaches to syndrome delineation and may be an efficient approach for risk estimation in monogenic disorders.

## Teaser

This cohort study examines the prevalence and penetrance of germline *DICER1* putative loss-of-function variants via genome-first ascertainment.

## Introduction

The phenotype-first approach, the traditional and proven strategy in clinical cancer genetics, historically has been productive in linking phenotype with germline variation. The Geisinger MyCode Community Health Initiative exemplifies an alternative to this method: the genome-first approach, in which individuals with pathogenic variants are ascertained on the basis of genotype. Specifically, the Geisinger-Regeneron DiscovEHR collaboration links electronic health records (EHRs) to population-scale exome sequencing to create a platform for genomic discovery, drug development, and clinical genomic implementation.^1^ The genome-first approach coupled with deep phenotyping demonstrated its utility and accuracy in ascertainment of the predictive value of *CFTR* [OMIM 602421] screening and cystic fibrosis.^2^ Previous analyses of these data have focused on more common genetic disorders (eg, *BRCA1/2* [OMIM 113705] and hypercholesterolemia).^3,4^

Heterozygous germline pathogenic variants in *DICER1* [OMIM 606421], an essential component of the microRNA (miRNA) processing pathway, underlie an autosomal dominant tumor-predisposition disorder that confers increased risk of a variety of rare and common neoplasms in children and adults.^5^ The neoplasm risks associated with pathogenic *DICER1* variants include pleuropulmonary blastoma (PPB, a lung sarcoma); cystic nephroma; Wilms tumor and renal sarcomas; Sertoli-Leydig cell tumor (SLCT); gynandroblastoma; thyroid nodules; thyroid cancer; and nasal, eye, pituitary and pineal tumors.^6,7,8^ Nonneoplastic syndrome manifestations include thyroid disease (especially multinodular goiter^7^), macrocephaly,^9^ retinal changes,^10^ and kidney and urinary tract anomalies.^11^ Although the prevalence of these *DICER1*-specific neoplasms is low, the prevalence of loss-of-function *DICER1* variants in the general population is estimated to be approximately 1 in 10 600 people and is more common than expected.^12^

As with many monogenic disorders, the ascertainment of individuals with pathogenic germline variants in *DICER1* is often triggered by symptomatic individuals. Typically, a child or adolescent who unsuspectingly harbors a germline *DICER1* loss-of-function variant presents with a lung, kidney, or ovarian mass. Pathologic diagnosis of a PPB, cystic nephroma, or SLCT then leads to genetic counseling, *DICER1* testing, and cascade testing in other family members. In this analysis, we apply the genome-first approach to a population-scale (>92 000 individuals), EHR-linked, exome-sequenced cohort to investigate the prevalence, risk, and phenotypic spectrum of individuals with germline putative loss-of-function (pLOF) variants in *DICER1.*

## Methods

### Cohort Description, Exome Sequencing, and Variant Annotation

The study cohort consisted of individuals who consented to participate in the MyCode Community Health Initiative, an institutional review board–approved program to create a biorepository of blood, serum, and DNA samples for broad research use, including genomic analysis. The *DICER1* variants in this study were derived from the first 92 296 participants, of whom 1165 (1.26%) were younger than 19 years. Exome sequencing for the DiscovEHR collaboration has been previously described in detail.^13^ Familial relationship was inferred using identity-by-descent analysis (PLINK software, version 1.9), and pedigrees were reconstructed using PRIMUS software.^14^ This study was approved by the General Institutional Review Board; participants in MyCode have provided broad consent for research use of their exome and EHR data. Imputed pedigrees such as those in this manuscript are therefore generated from the exome data and not through recontact of the patient. Under the rules of the institutional review board, imputed pedigrees do not require additional consent. This study followed the Strengthening the Reporting of Observational Studies in Epidemiology (STROBE) reporting guideline.

### EHR Review

Individuals with pLOF *DICER1* variants identified by germline exome sequencing underwent open record review through a Geisinger Institutional Review Board–approved protocol. We developed an abstraction form to document the EHR imaging and interpretations, hospitalizations, surgical procedures, and medical history. In addition, the data abstraction was guided by thyroid-related clinical traits (unigoiter, multinodule goiters, thyrotoxicosis, and thyroidectomy) and all malignant tumors obtained from a structured EHR database. Previous EHR reviews found that reviewers were able to locate more information when they were guided by the diagnosis or procedure, especially when the dates of diagnosis or procedure were included. Three independent reviewers (K.M., J.H., and Y.H.), including a clinical geneticist (K.M.) and an endocrinologist (Y.H.), performed the EHR review, which included examining clinical laboratory test results, problem lists, practitioner notes, and scanned documents. In addition, the reviewers also performed broad searches in the EHR for the terms *DICER1*, *DICER*, *DICER syndrome*, *genetics*, *pleuropulmonary blastoma*, and *ppb*.

### *DICER1* Variant Classification and Matching of Heterozygote Carriers to Noncarriers

We applied our published scheme with modifications to classify *DICER1* variation into 4 categories: pLOF (similar to “pathogenic” by the American College of Medical Genetics and Genomics and the Association for Molecular Pathology [ACMG-AMP] criteria),^15^ predicted deleterious (similar to “likely pathogenic” by the ACMG-AMP criteria), variant of uncertain significance (VUS), and likely benign (LB).^12,16^ For statistical analyses, individuals with germline *DICER1* pLOF, predicted deleterious, VUS, or LB variants (carriers) were matched to noncarriers (individuals with reference *DICER1* sequence) by sex, age, race, and smoking status; for analyses of missense variants, smoking was used as a covariate. CADD^17^ and REVEL^18^ (bioinformatic variant pathogenicity prediction methods) were also used to investigate the utility of these tools in predicting the consequence of germline *DICER1* variation. A CADD score of 20 or higher or a REVEL score of 0.5 or higher were considered damaging based on the developer’s recommendation.

### EHR-Linked Phenotypes in Carriers and Noncarriers

We used the *International Classification of Diseases, Ninth Revision (ICD-9)*, *International Statistical Classification of Diseases and Related Health Problems, Tenth Revision (ICD-10)*, and procedure or *Current Procedural Terminology* codes (eTable 1 in the Supplement) to find diagnoses related to thyroid disease (including thyroidectomy) and malignant tumors in the EHR. For individuals with *DICER1* pLOF variants, cancer diagnoses were confirmed using data in the Geisinger Cancer Registry (1943-2017); nonmelanoma skin cancers were excluded. We also searched the cancer registry for known *DICER1*-associated tumors in the MyCode cohort, regardless of germline *DICER1* status.

### Pathology Review and *DICER1* Somatic Sequencing

Archival pathology materials on tumors were obtained and subjected to DNA extraction and *DICER1* sequencing, as previously described.^19^

### Statistical Analysis

Kaplan-Meier analyses with contingency tables were performed for thyroid and malignant tumor phenotypes using the R version 3.6.0 survival package (R Foundation for Statistical Computing).^20^ Dates of entry of each MyCode participant into the Geisinger registry were used from the start of the Geisinger EHR to February 1, 2018. Statistical difference in Kaplan-Meier curves was determined using Cox proportional hazards analysis and likelihood ratio test^21^; 2 × 2 contingency tables were then used to test for association of variants and clinical traits by odds ratios (ORs) and 95% CIs. The significance of the association was evaluated using the Fisher exact test values. Associations between *DICER1* variants and phenotypes were considered significant if Bonferroni-corrected multiple testing resulted in *P* < .008 (calculated for α = .05 for 6 clinical traits tested).

## Results

### Germline *DICER1* pLOF Variants

A total of 92 296 individuals (mean [SD] age, 59 [18] years; 98% white; 60% female) participated in the study. The cohort demographic characteristics were similar to those previously reported in DiscovEHR studies^3^ and summarized in eTable 2 in the Supplement. Table 1 gives the numbers of unique variants and individual carriers of *DICER1* variants (full details in eTable 3 in the Supplement), classified according to our previously published scheme.^12^ There were 12 unique *DICER1* pLOF variants, including 5 (42%) frameshift, 2 (17%) stop-gain, 3 (25%) canonical splice site, 1 (8%) initiator loss, and 1 (8%) hotspot codon missense variation; all variants were confirmed by Sanger sequencing. The 12 unique pLOF variants were observed in 25 individuals (1 in 3700 people). Pedigrees inferred from identify-by-descent analysis^14^ indicated that 9 of the pLOF variant carriers in 4 pedigrees were closely related (eFigure 1 in the Supplement). When calculated using only unrelated individuals (n = 20), the prevalence was 1 in 4600.

**Table 1.** Unique and Total Numbers for 4 Categories of *DICER1* Variation in 92 296 DiscovEHR Participants, With Estimation of Prevalence for Variation, With and Without Familial Correction^a^

Criteria	Number and prevalence
Putative loss of function or hotspot	
MAF < 0.001	12 Unique variants
Protein-truncating variants	25 Total carriers: 1 in 3700
Stop-gained, frameshift, initiation loss, or canonical splice site (missense hotspot [E1705, D1709, G1809, D1810, E1813] and variant reported as pathogenic in ≥1 publication)	20 Unrelated carriers; 1 in 4600 people
Predicted deleterious	
MAF < 0.001	40 Unique variants
Nonsynonymous missense	84 Total carriers
Located in nonhotspot and bioinformatics pathogenicity prediction (metaSVM score, deleterious)	1 In 1100 people
Variant of uncertain significance	
MAF < 0.001	8 Unique variants
In-frame deletion or insertion or stop-loss or start-gain	27 Total carriers
Splice region (ada/rf >0.6)	1 In 3400 people
Likely benign	
MAF < 0.001	424 Unique variants
Nonsynonymous missense	2289 Total carriers
Bioinformatics pathogenicity prediction (metaSVM score, tolerated) (synonymous variants)	

Abbreviations: ada, adaptive boosting; MAF, minor allele frequency; rf, random forest.

^a^ See eTable 2 in the Supplement for information on specific variants.

### Manual EHR Review of Individuals With *DICER1* pLOF Variation

The demographic characteristics and thyroid-related clinical features of the 25 participants with *DICER1* pLOF variation are given in Table 2 and are similar to the sequenced cohort (eTable 4 in the Supplement). Notably, 2 *DICER1* pLOF carriers were deceased. One was a White woman (patient 24) with a history of pineoblastoma (when she was an adolescent) and meningioma (in her 40s) with a germline *DICER1* frameshift variant (p.Ser1823Valfs) who died in her 40s; cause of death was not documented in the EHR. The second was a White man (patient 4) with an absent germline *DICER1* start site (p.Met1?) and a history of hypothyroidism who had been taking levothyroxine since his 40s and had renal cell carcinoma in his late 40s (and had undergone nephrectomy), diverticular rupture and partial colectomy (in his 50s), ultrasonic evidence of pancreatic cysts (in his 60s), and liver cirrhosis. He died in his 70s from complications of cirrhosis.

**Table 2.** Demographic and Clinical Characteristics of Germline *DICER1* Pathogenic Variant Carriers^a^

Patient No./Sex/Age, y/Race	*DICER1* cDNA	*DICER1* protein	Age at diagnosis						Minimum encounter age, y^e^	Time encountered, y	No. of encounters
			Hypothyroidism^b^	Uninodular goiter^b^	Other goiter^b^	Thyrotoxicosis^b^	Thyroidectomy^c^	Tumor site, morphologic type/age, y^d^			
1/F/<10/AA	c.-45-2A>G	NA	NA	NA	NA	NA	NA	NA	0.0	7.3	40
2/F/50s/W	c.2T>C	Met1?	50s	NA	NA	NA	NA	NA	40s	14.4	16
3/F/80s/W	c.2T>C	Met1?		NA	NA	NA	NA	NA	60s	11.9	30
4/M/70s/W	c.2T>C	Met1?	70s	NA	NA	NA	NA	Kidney, RCC/40s	60s	10.9	153
5/F/20s/W	c.2T>C	Met1?	NA	NA	NA	NA	NA	NA	Teens	5.4	11
6/F/30s/W	c.2T>C	Met1?	30s	NA	NA	NA	NA	NA	Teens	15.3	76
7/M/60s/W	c.381delinsTA	Glu128Argfs	NA	60s	NA	NA	NA	NA	40s	15.8	56
8/M/30s/W	c.381delinsTA	Glu128Argfs	NA	NA	NA	NA	NA	NA	20s	14.3	54
9/F/40s/W	c.1030delinsAT	Phe344Ilefs	NA	40s	40s	40s	40s	NA	40s	1.8	10
10/F/80s/W	c.1030delinsAT	His341Glnfs	NA	NA	NA	NA	NA	NA	70s	17.0	143
11/F/50s/W	c.1525C>T	Arg509Ter	50s	NA	NA	NA	NA	NA	50s	2.4	6
12/F/70s/W	c.1525C>T	Arg509Ter	NA	NA	NA	NA	NA	NA	60s	2.3	24
13/F/20s/W	c.2805-2delinsTA	NA	NA	NA	NA	NA	NA	NA	<10	15.6	41
14/F/70s/W	c.2805-2delinsTA	NA	NA	70s	NA	NA	NA	Thyroid gland, benign/60s	50s	15.9	61
15/F/30s/W	c.2805-2delinsTA	NA	NA	NA	NA	NA	NA	NA	30s	2.9	26
16/M/60s/W	c.2805-2delinsTA	NA	NA	NA	NA	NA	NA	NA	50s	2.7	249
17/M/60s/W	c.2805-2delinsTA	NA	NA	NA	NA	NA	NA	NA	50s	7.0	15
18/F/40s/W	c.2805-2delinsTA	NA	30s	NA	NA	NA	NA	NA	30s	10.1	10
19/F/60s/W	c.2805-2delinsTA	NA	60s	NA	NA	NA	NA	NA	50s	16.5	135
20/F/50s/W	c.4050 + 1G>A	NA	50s	NA	NA	50s	NA	Thyroid gland, PC/20s	30s	18.0	95
21/M/70s/W	c.5003_5004delinsT	Asn1668Ilefs	70s	NA	NA	NA	NA	NA	50s	16.0	167
22/M/40s/W	c.5003_5004delinsT	Asn1668Ilefs	40s	40s	30s	NA	NA	Thyroid gland, PC/30s	30s	16.6	93
23/F/80s/W	c.5127T>A	Asp1709Glu	NA	NA	NA	NA	NA	Breast, benign/70s	60s	15.9	120
24/F/40s/W	c.5467_5475delinsG	Ser1823Valfs	40s	NA	NA	NA	NA	Pineoblastoma/teens; meningioma/40s	30s	6.5	29
25/F/20s/W	c.5622C>A	Tyr1874Ter	NA	NA	NA	NA	NA	NA	Teens	13.9	43

Abbreviations: AA, African American/Black; NA, not applicable; PC, papillary carcinoma; RCC, renal cell carcinoma; W, White.

^a^ Clinical diagnoses for thyroid-related conditions are shown as the earliest age (in years) at which the clinical features were recorded. Recorded visits to primary care practitioners in electronic health records are presented as encounters with earliest age recorded, number of years of visits, and total number of visits.

^b^ *International Classification of Diseases, Ninth Revision (ICD-9)* or *International Classification of Diseases, Tenth Revision (ICD-10)* diagnosis codes.

^c^ *Current Procedural Terminology* codes.

^d^ Tumor registry except for benign thyroid gland, which was recorded as *ICD-9* or *ICD-10* codes.

^e^ The age at which the patient had the first encounter with a Geisinger provider, according to the electronic health record.

A woman in her 80s (patient 23) harbored a germline *DICER1* p.Asp1709Glu hotspot variant (variant allele frequency, 23%). This variant has been reported to be somatically mutated in fetal adenocarcinoma of the lung,^22^ SLCT, and primitive germ cell tumor (yolk sac).^23^ In addition, in silico bioinformatic tools (REVEL score, 0.89; metaSVM score, deleterious; CADD score, 25.5) predict more severe consequences on protein function. However, EHR review found a history of liver cysts on abdominal ultrasonography and a history of breast biopsy with no evidence of atypia or malignant tumor. We note that mesenchymal hamartoma, a cystic lesion of the liver, is reported to arise from a *DICER1* mutation.^24^ This patient has had 120 encounters with Geisinger physicians during the last 16 years; thus, her lack of thyroid-related clinical findings is not attributable to a lack of medical care.

Notably, the terms *DICER1* or *DICER* did not appear in any of the EHRs of participants with a *DICER1* pLOF variant. Furthermore, EHR review found that most carriers had sufficient data in the EHR, with a median number of encounters of 43 (range, 6-249) and a median length of follow-up of 14 years (range, 2-18 years). These findings are comparable to the matched noncarriers, who had a median number of encounters of 62 (range, 0-974) and a median length of follow-up of 14 years (range, 0-21 years).

### Significant Excess of Malignant Thyroid Tumors in Individuals With a *DICER1* pLOF Variant

**Figure 1.**
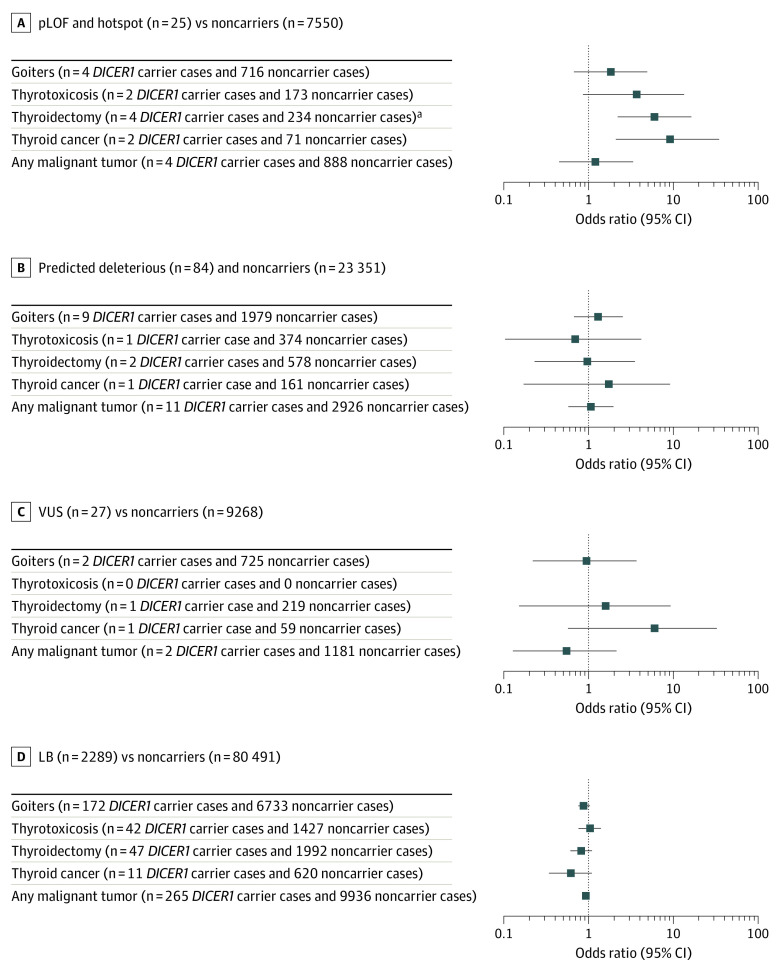
Association of Germline *DICER1* Variants and Thyroid Phenotypes and Malignant Tumors Stratified by Pathogenicity Odds ratios (95% CIs) for the prevalence of each phenotype for individuals from the putative loss-of-function (pLOF) and hotspot variant, predicted deleterious, variant of uncertain significance (VUS), and likely benign (LB) groups compared with noncarriers (DiscovEHR participants who were noncarriers of a *DICER1* variant) were calculated using the Fisher exact test. Carriers and noncarriers were matched by sex, race, and smoking status. Numbers in parentheses after each phenotype denote counts in *DICER1* carrier cases and noncarrier cases (eTable 5 in the Supplement). ^a^*P* < .008 is considered significant with Bonferroni correction for multiple testing.

Four biopsy-proven malignant tumors (excluding nonmelanoma skin cancers), identified from the Geisinger Cancer Registry, were observed in the 25 individuals with a *DICER1* pLOF variants (Table 3). Of these, high-quality DNA was available only from the papillary thyroid carcinoma, which harbored multiple somatic *DICER1* hotspot variants, in addition to the germline p.Asn1668Ilefs. In the 25 individuals with a *DICER1* pLOF variant compared with 7550 noncarriers matched by age, sex, race, and smoking status, we observed significantly greater associations with thyroid cancer (OR, 9.2; 95% CI, 2.1-34.7; *P* = .02) and thyroidectomy (OR, 6.0; 95% CI, 2.2-16.3; *P* = .007); thyroidectomy remained significant after Bonferroni correction (Figure 1; eTable 5 in the Supplement). There was no excess of malignant thyroid tumors in carriers of predicted deleterious, VUS, and LB variants compared with matched noncarriers.

**Table 3.** Somatic *DICER1* Variants Observed in Malignant Tumors Arising in Individuals With Germline *DICER1* Putative Loss-of-Function Variant and Somatic *DICER1* Variants in *DICER1*-Associated Tumors in MyCode Participants Without Germline *DICER1* Variants

Malignant tumor	Sex/age at diagnosis, y	Germline *DICER1* variant	Germline VAF,^a^ %	Somatic *DICER1* variant	Somatic VAF,^a^ %
Papillary thyroid carcinoma	F/20s	c.4050 + 1G>A	47	DNA failed quality control	NA
Papillary thyroid carcinoma	M/30s	c.5003_5004delinsT (Asn1668fs)	50	p.Asp1810Val and p.Asp1810Tyr	33 And 2
Pineoblastoma	F/Teens	c.5467_5475delinsG (Ser1823fs)	39	DNA concentration insufficient	NA
Renal cell carcinoma	M/40s	c.2T>C (Met1)	51	DNA failed quality control	NA
Ovarian Sertoli-Leydig cell tumor	F/40s	None	NA	p.Arg624Ter and p.Asp1703Asn	44 And 59
Rhabdomyosarcoma, endometrial	F/Teens	None	NA	p.Glu1600fs and p.Gly1809Arg	16 And 29

Abbreviations: NA, not applicable; VAF, variant allele frequency.

^a^ Variant allele frequency was calculated as (alternate reads/total reads) × 100.

### *DICER1*-Associated Malignant Tumors in Nongermline Carriers of *DICER1* Coding and Splicing Variants

We searched the EHR for known rare *DICER1*-associated tumors (ovarian SLCT, endometrial rhabdomyosarcoma, nasal chondromesenchymal hamartoma, and PPB). Of 8574 female participants with tumor-biopsy records, there was 1 poorly differentiated ovarian SLCT in a woman in her 40s and an endometrial rhabdomyosarcoma in a female patient in her teens (Table 3). In both tumors, somatic *DICER1* pLOF and hotspot variants were identified (Table 3). Neither person carried a germline exonic *DICER1* sequence or copy number variant.

### Malignant Tumors Observed in Individuals With Predicted Deleterious *DICER1* Variants and VUS

To investigate the frequency of malignant tumors in carriers of a germline *DICER1* predicted deleterious variant (metaSVM score, deleterious), we examined the number and type of malignant tumors (excluding nonmelanoma skin cancer) recorded in the Geisinger Cancer Registry (eTable 6 in the Supplement). There were 40 *DICER1* predicted deleterious variants in 84 individuals. No malignant tumors were observed in individuals with 32 (80%) of these variants. Twelve carriers (20%) of 8 predicted deleterious variants had at least 1 malignant tumor; DNA of suitable quality was obtainable from a few tumors. One germline variant (p.Gly1364Ala) was observed in 2 individuals: a woman in her 30s with thyroid cancer and a man in his 40s with seminoma. Somatic sequencing of the seminoma identified a *DICER1* p.Gly1809Arg hotspot variant (as previously reported^25^) but with a very low variant allele frequency (1.5%). One germline variant (p.Thr806Met) was observed in 4 individuals, each with 1 cancer. Of the 4 tumors in germline *DICER1* p.Thr806Met carriers, high-quality DNA was obtainable from 1 (sigmoid colon adenocarcinoma), which harbored no additional *DICER1* somatic variation. In 27 VUS carriers, there was one woman in her 20s (germline p.Asn1393_Thr1394insAsn) with a follicular thyroid carcinoma; on somatic sequencing, no *DICER1* hotspot variant was detected.

### Risk of Thyroid Phenotypes in Individuals With *DICER1* Variation

Figure 1 and eTable 5 in the Supplement give the ORs of sex-, race-, and smoking-matched carriers and noncarriers for development of thyroid disease or thyroidectomy for the 4 categories of *DICER1* variation. Overall, there was a significant increase in the risks of thyroidectomy and thyroid cancer in pLOF carriers; however, only the risk of thyroidectomy remained significant after Bonferonni correction for multiple testing. There was no significant increase the prevalence of goiters or thyrotoxicosis. In addition, we did not observe an increase in the prevalence of cancers in all pLOF carriers compared with noncarriers. eFigure 2 in the Supplement shows heatmaps of age at onset for the thyroid phenotypes stratified by different *DICER1* variation subclasses.

To further investigate the 2 individuals with a *DICER1* pLOF variant and a diagnosis of thyrotoxicosis or hyperthyroidism, an endocrinologist performed an EHR review. The first patient, a woman in her 50s (patient 20) (Table 2), had a thyroid cancer treated by thyroidectomy in her 20s and was prescribed levothyroxine. There was no EHR laboratory or pathology documentation of an organic cause underlying her hyperthyroidism. The second patient, a woman in her 40s (patient 9), had EHR documentation of Graves disease, including pathology review of her thyroid and an abnormal radioactive iodine scan.

### Use of the EHR to Inform *DICER1* Missense Variation Interpretation

**Figure 2.**
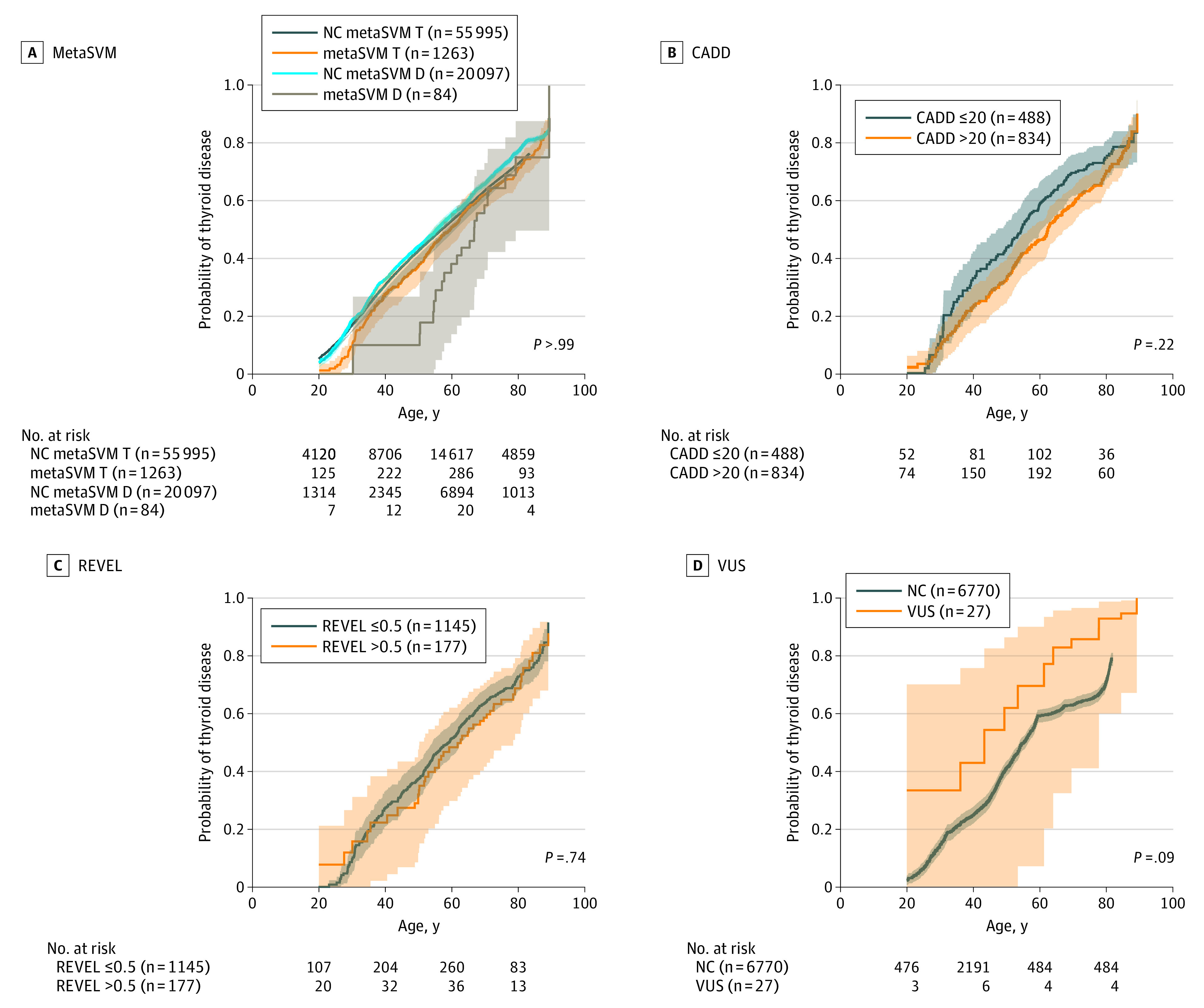
Probability of Thyroid Phenotypes (Goiters, Thyrotoxicosis, Hypothyroidism, Thyroidectomy, and Thyroid Cancer) Over Time Kaplan-Meier plots with left-truncation-bias correction in all individuals with bioinformatically predicted *DICER1* missense variation using in silico prediction tool scores, including metaSVM, CADD, and REVEL, as well as variant of uncertain significance (VUS) vs controls. The metaSVM data use separate sets of controls designed for T (tolerated) and D (deleterious). NC indicates noncarrier of *DICER1* variants.

To more thoroughly explore the consequence of germline *DICER1* missense variation, we investigated the probability of thyroid disease (defined as an incidence of goiter, hypothyroidism, cysts, benign thyroid tumors, or thyrotoxicosis) in carriers of *DICER1* missense variants predicted to be damaging by 3 bioinformatic tools (using the thresholds recommended by the developers of metaSVM, CADD, and REVEL), compared with sex-, race-, and age-matched noncarriers (Figure 2). No significant differences were observed in these analyses.

## Discussion

This cohort study is, to our knowledge, the first study to use EHR-linked exome sequencing in a large cohort to investigate the *DICER1* tumor-predisposition disorder and provides critical experience regarding the strengths and limitations for genome-first investigations of monogenic tumor-predisposition disorders in general. From 92 296 exomes, this study estimated germline *DICER1* pLOF variant prevalence to range from 1 in 3700 to 1 in 4600 people. This is more than twice as common (even after adjustment for relatedness) than the previous estimate of 1 in 10 600 people^12^ (non-Finnish European: 1 in 9058), which was based on frequency of *DICER1* variants in the noncancer cohort of the Exome Aggregation Consortium (n = 53 105) and was approximately 60% of the size of the cohort used in the current analysis. This refined estimate is comparable to the prevalence of germline pathogenic *DICER1* variants in The Cancer Genome Atlas (1 in 4600; n = 9173 exomes).^16^ It is also comparable to the prevalence of other common genetic disorders, such as fragile X,^26^ 22q11.2 deletion syndrome,^27^ and neurofibromatosis type 1.^28^ The discovery, through genome-first approaches, that pathogenic variant prevalence in *DICER1* (and in other important tumor-predisposition genes, such as *BRCA1* and *BRCA2*^4^) is more common than expected has important clinical implications. First, accurate estimates of variant prevalence (and penetrance) are needed to assess whether a variant is too common to be causative for a mendelian disorder of interest^29^; these determinations are crucial in the development of variant interpretation rules set by ClinGen based on the ACMG-AMP^15^ criteria. These standardized, peer-reviewed rule sets are important in the identification of at-risk individuals. Second, a higher prevalence suggests that many more people are at risk than previously recognized, which influences a priori risk estimates in genetic counseling. Third, higher prevalence highlights the importance in risk estimation of accurate penetrance estimates, which in *DICER1* carriers is known to depend on age and sex.^8^ However, estimates of penetrance in *DICER1* carriers may also need to be tiered based on family history. Family history is important in risk estimation in some monogenic tumor-predisposition disorders, such as those associated with pathogenic variants in *BRCA1/2* or *PALB2* (OMIM 610355).^30^

Among the 25 individuals with a germline *DICER1* pLOF variant, 4 of 25 (16%) had a history of malignant tumor, which is comparable (by 50 years of age) to the frequency of neoplasms in the largest registry- and clinic-based (phenotype-first) *DICER1* studies published to date.^19,31,32^ However, the penetrance estimate in the current analysis is conservative and limited by the small number of observations and multiple causes of underascertainment. For example, pediatric and other early-onset aggressive cancers would be undercounted, especially if they lead to death or otherwise make it less likely for an individual to enroll in MyCode. In addition, individuals with goiter and thyroidectomy, especially at an early age, would have abrogated risk of developing thyroid disease.

This study was able to identify less common (pineoblastoma) and more common (thyroid carcinomas) *DICER1*-associated neoplasms as well as a potentially novel *DICER1*-associated neoplasm (renal cell carcinoma) that merits additional follow-up. In addition, this study identified 2 known *DICER1*-associated neoplasms (SLCT and rhabdomyosarcoma) in patients without any germline *DICER1* variation, consistent with low-level mosaicism or tumor-only *DICER1* variation. Accurate estimates of the prevalence of *DICER1* mosaicism and tumor-confined variation are also crucial to providing useful clinical risk estimates and genetic counseling; this study found that the genome-first approach is effective in providing data toward determining those estimates. The data indicate how genome-first ascertainment informs phenotype. For example, multiple publications indicate that germline mosaicism for *DICER1* hotspot codon variation is linked to a severe overgrowth phenotype.^19,31,32^ Surprisingly, this study found an apparently healthy woman in her 80s who harbored a previously reported, Sanger-confirmed hotspot variant (variant allele frequency, 23%). It was not possible to perform clinical phenotyping in this participant and the variant could have arisen from clonal hematopoiesis, although this has not been reported in *DICER1*. If germline, this finding suggests that the phenotype arising from *DICER1* hotspot variation may not be as severe as previously reported and illustrates an advantage of genome-first ascertainment.

It is notable that on manual review of the EHR, the term *DICER1* did not appear in any of the records of the 25 participants with a pLOF *DICER1* variant, even in the records of patients with a known *DICER1*-associated tumor or thyroid phenotype. This finding is perhaps not surprising given the older age of the cohort and the lack of specificity of the phenotypes. However, these participants and their relatives (especially children and female individuals) are at risk for a set of well-characterized *DICER1*-associated tumors that can be prospectively identified and managed^33^ with established *DICER1*-specific surveillance guidelines.^34^ Such surveillance is particularly appealing (and, with subsequent interventions, potentially curative) for highly morbid *DICER1*-associated tumors, such as PPB and ovarian sex-cord stromal tumors. However, given the reduced penetrance of *DICER1* pathogenic variants, many at-risk individuals will never develop problems and will thus not benefit; rather, they will be harmed by false-positive results, follow-up biopsy, and costs. These issues will become only more magnified and urgent for *DICER1* and other genes, with widespread population-scale sequencing, obligatory return of secondary findings,^35^ and germline results in tumor sequencing.^36^ The solution is to optimize the risk-benefit ratios in an age-, sex-, and race-specific way through the development of accurate penetrance estimates using additional biomarkers, better imaging, and surveillance modalities. The influence (if any) of family history, modifier genes, *DICER1* genotype and phenotype correlates, polygenic risk, and environmental exposures needs to be identified. To do this, both genome-first and phenotype-first approaches are likely needed.

### Limitations

This study has limitations. The cohort, though large and clinically unselected, is 98% White and required enrollment in a health care system. Even in a cohort of 92 296 individuals, the number of *DICER1* pLOF variants was small. The study focused on germline and somatic sequence–based alterations in *DICER1* and did not evaluate methylation or copy-number variation (except small [<10 base pairs] deletions) at this locus. The phenotyping was limited to the accuracy of the EHR. Cancer or thyroid diagnoses made outside of the Geisinger registry may have been missed, although this is less likely given the EHR review performed for pLOF carriers. Pediatric cancer diagnoses may be incompletely ascertained, especially for participants enrolling in the health care system as adults. Last, individuals with the most damaging germline *DICER1* variation may have died before having an opportunity to enroll in the MyCode cohort.

## Conclusions

To our knowledge, this is the first study to ascertain individuals with *DICER1* variation (as an archetypal monogenic tumor-predisposition disorder) based on a genome-first approach rather than through a previously established *DICER1*-related phenotype. Use of the genome-first approach not only complements more traditional approaches to syndrome delineation but also may be an efficient approach for risk estimation in monogenic disorders.

## Notes

Supplement. **eTable 1.** The International Classification of Diseases, Ninth and Tenth Revisions (ICD9/10) Billing Codes to Query the Geisinger EHR for Diagnoses Related to Thyroid Disease (Including Thyroidectomy) and Malignancy (A) and the Current Procedural Terminology (CPT) Codes to Query the Geisinger EHR for Diagnoses Related to Thyroid Disease (Including Thyroidectomy) and Malignancy (B) **eTable 2.** Demographic Characteristics of the Geisinger DiscovEHR Cohort **eTable 3.** List of All *DICER1* Variants (P, LP, VUS, LB), MAF in Reference Databases (Exac_nonTCGA_NFE, Gnomad_Exome_NFE), MetaSVM, CADD, REVEL, HGMD, and Clinvar Classification **eTable 4.** Demographic Characteristics of 25 Subjects With Putative Loss-of-Function or Hotspot *DICER1* Variation in the Geisinger DiscovEHR Cohort **eTable 5.** Association of Germline *DICER1* Variants and Thyroid Phenotypes Stratified by Pathogenicity **eTable 6.** Germline and Somatic *DICER1* Variants in Cancers of Individuals With Germline *DICER1* Predicted Deleterious Variants **eFigure 1.** Inferred Pedigrees of Carriers of *DICER1* Putative Loss-of-Function Variation **eFigure 2.** Age of Diagnosis and Frequency of Diagnosis for Thyroid Conditions in Carriers and Non-Carriers (NC) of *DICER1* Variants
