# Supplementary material for: A Genome-First Approach to Characterize DICER1 Pathogenic Variant Prevalence, Penetrance, and Phenotype
Source: JAMA Netw Open. 2021 Feb 25;4(2):e210112. doi: 10.1001/jamanetworkopen.2021.0112 (PMC7907958; doi:10.1001/jamanetworkopen.2021.0112)
Supplement: Supplement. — eTable 1. The International Classification of Diseases, Ninth and Tenth Revisions (ICD9/10) Billing Codes to Query the Geisinger EHR for Diagnoses Related to Thyroid Disease (Including Thyroidectomy) and Malignancy (A) and the Current Procedural Terminology (CPT) Codes to Query the Geisinger EHR for Diagnoses Related to Thyroid Disease (Including Thyroidectomy) and Malignancy (B) eTable 2. Demographic Characteristics of the Geisinger DiscovEHR Cohort eTable 3. List of All DICER1 Variants (P, LP, VUS, LB), MAF in Reference Databases (Exac_nonTCGA_NFE, Gnomad_Exome_NFE), MetaSVM, CADD, REVEL, HGMD, and Clinvar Classification eTable 4. Demographic Characteristics of 25 Subjects With Putative Loss-of-Function or Hotspot DICER1 Variation in the Geisinger DiscovEHR Cohort eTable 5. Association of Germline DICER1 Variants and Thyroid Phenotypes Stratified by Pathogenicity eTable 6. Germline and Somatic DICER1 Variants in Cancers of Individuals With Germline DICER1 Predicted Deleterious Variants eFigure 1. Inferred Pedigrees of Carriers of DICER1 Putative Loss-of-Function Variation eFigure 2. Age of Diagnosis and Frequency of Diagnosis for Thyroid Conditions in Carriers and Non-Carriers (NC) of DICER1 Variants [file jamanetwopen-e210112-s001.pdf]

Supplementary Online Content

Mirshahi UL, Kim J, Best AF, et al. A genome-first approach to characterize *DICER1* pathogenic variant prevalence, penetrance, and phenotype. *JAMA Netw Open*. 2021;4(2):e210112. doi:10.1001/jamanetworkopen.2021.0112

eTable 1. The International Classification of Diseases, Ninth and Tenth Revisions (ICD9/10) Billing Codes to Query the Geisinger EHR for Diagnoses Related to Thyroid Disease (Including Thyroidectomy) and Malignancy (A) and the Current Procedural Terminology (CPT) Codes to Query the Geisinger EHR for Diagnoses Related to Thyroid Disease (Including Thyroidectomy) and Malignancy (B)

eTable 2. Demographic Characteristics of the Geisinger DiscovEHR cohort

eTable 3. List of All *DICER1* Variants (P, LP, VUS, LB), MAF in Reference Databases (Exac_Nontcga_NFE, Gnomad_Exome_NFE), MetaSVM, CADD, REVEL, HGMD, and Clinvar Classification

eTable 4. Demographic Characteristics of 25 Subjects with Putative Loss-of-Function or Hotspot *DICER1* Variation in the Geisinger DiscovEHR Cohort

eTable 5. Association of Germline *DICER1* Variants and Thyroid Phenotypes Stratified by Pathogenicity

eTable 6. Germline and Somatic *DICER1* Variants in Cancers of Individuals With Germline *DICER1* Predicted Deleterious Variants

eFigure 1. Inferred Pedigrees of Carriers of *DICER1* Putative Loss-of-Function Variation

eFigure 2. Age of Diagnosis and Frequency of Diagnosis for Thyroid Conditions in Carriers and Non-Carriers (NC) of *DICER1* Variants

This supplementary material has been provided by the authors to give readers additional information about their work.

© 2021 Mirshahi UL et al. *JAMA Network Open.*

eTable 1A. The International Classification of Diseases, ninth and tenth revisions (ICD9/10) billing codes to query the Geisinger EHR for diagnoses related to thyroid disease (including thyroidectomy) and malignancy

THYROID CONDITIONS	ICD9 CODE	ICD9 CODE DESCRIPTION	ICD10 CODE
BENIGN THYROID CANCER	226	Benign neoplasm of thyroid glands	D34
DISORDERS OF THYROID	246.8	Other specified disorders of thyroid	E07.89, E89.0, E03.4
DISORDERS OF THYROID	246.9	Unspecified disorder of thyroid	E07.9, R94.6
GOITER	242.01	Toxic diffuse goiter with mention of thyrotoxic crisis or storm	E05.01
GOITER	242.01	Toxic diffuse goiter with mention of thyrotoxic crisis or storm	E05.01
GOITER	242.3	Toxic nodular goiter, unspecified type, without mention of thyrotoxic crisis or storm	E05.20
GOITER	240.9	Goiter, unspecified	E04.0, E04.9, E01.0, E01.2, E04.8
GOITER	241.9	Unspecified nontoxic nodular goiter	E04.9, E04.0, E04.2
GOITER	242.0	Toxic diffuse goiter without mention of thyrotoxic crisis or storm	E05.00
GOITER	242.0	Toxic diffuse goiter	E05.00C E05.01
GOITER	242.00	Toxic diffuse goiter without mention of thyrotoxic crisis or storm	E05.00
HYPERTHYROIDISM	242.01	Toxic diffuse goiter with mention of thyrotoxic crisis or storm	E05.01
HYPERTHYROIDISM	242.21	Toxic multinodular goiter with mention of thyrotoxic crisis or storm	E05.21
HYPERTHYROIDISM	242.4	Thyrotoxicosis from ectopic thyroid nodule without mention of thyrotoxic crisis or storm	E05.30
HYPERTHYROIDISM	242.81	Thyrotoxicosis of other specified origin with mention of thyrotoxic crisis or storm	E05.41
HYPERTHYROIDISM	242.91	Thyrotoxicosis without mention of goiter or other cause, with mention of thyrotoxic crisis or storm	E05.91
HYPOTHYROIDISM	244.8	Other specified acquired hypothyroidism	E03.8, E03.4, E03.9, E0.32, E02
HYPOTHYROIDISM	244.9	Unspecified acquired hypothyroidism	E03.9, E03.8C IMO0001, E03.3
IATROGENIC THYROID CONDITIONS	244.3	Other iatrogenic hypothyroidism	E03.2
IATROGENIC THYROID CONDITIONS	245.4	Iatrogenic thyroiditis	E06.4
IATROGENIC THYROID CONDITIONS	245.4	Iatrogenic thyroiditis	E06.4
MALIGNANCY	193	Malignant neoplasm of thyroid gland	C73
MALIGNANCY	V10.87	Personal history of malignant neoplasm of thyroid	Z85.850
MULTINODULAR GOITER	241.1	Nontoxic multinodular goiter	E04.2
MULTINODULAR GOITER	242.21	Toxic multinodular goiter with mention of thyrotoxic crisis or storm	E05.21
MULTINODULAR GOITER	242.21	Toxic multinodular goiter without mention of thyrotoxic crisis or storm	E05.21
OTHER THYROID CONDITIONS	244.2	Iodine hypothyroidism	E01.8
OTHER THYROID CONDITIONS	246.8	Other specified disorders of thyroid	E07.89
OTHER THYROID CONDITIONS	246.9	Unspecified disorder of thyroid	E07.9
SIMPLE GOITER/GOITER	240.0	Goiter, specified as simple	E04.0, E04.9, E01.0, E01.2, E04.8
THYROIDITIS	154	Thyroiditis, unspecified	E06.9
THYROIDITIS	222.3	Acute thyroiditis	E06.0
THYROIDITIS	245.2	Chronic lymphocytic thyroiditis	E06.5
THYROIDITIS	245.8	Other and unspecified chronic thyroiditis	E06.5
UNINODULAR GOITER	242.1	Toxic uninodular goiter without mention of thyrotoxic crisis or storm	E05.10

© 2021 Mirshahi UL et al. *JAMA Network Open.*

UNINODULAR GOITER	241.0	Nontoxic uninodular goiter	E04.1

© 2021 Mirshahi UL et al. *JAMA Network Open.*

eTable 1B. The Current Procedural Terminology (CPT) codes to query the Geisinger EHR for diagnoses related to thyroid disease (including thyroidectomy) and malignancy

THYROID CONDITIONS	ICD9 CODE DESCRIPTION	PCT CODE
THYROID SURGERY	Removal of thyroid gland	60240
THYROID SURGERY	Partial removal of thyroid lobe	60210
THYROID SURGERY	Removal of thyroid for tumor	60252
THYROID SURGERY	Removal of thyroid lobe, total	60220
THYROID SURGERY	Radical thyroid surgery for tumor	60254
THYROID SURGERY	Removal of thyroid lobe, total	60225
THYROID SURGERY	Removal of thyroid gland	60271
THYROID SURGERY	Removal of thyroid gland	60270
THYROID SURGERY	Partial removal of thyroid lobe	60212

© 2021 Mirshahi UL et al. *JAMA Network Open.*

eTable 2. Demographic Characteristics of the Geisinger DiscovEHR cohort

Variable	Values
Number of individuals	92,296
Race, %	
White	98
African American/Black	1.7
Others	0.3
Ethnicity, %	
Non-Hispanic	96
Hispanic or Latino	1.7
Unknown	2
Sex, % female	60
Current age, years	
Mean	59
Median	59
Range	2 – 89
BMI, kg/m^2	
Median	31
Range	14 – 64
Number of clinical encounters	
Mean	73
Median	57
Range	1 - 1245
Years of clinical encounters	
Mean	12
Median	14
Range	0-21
Smoking status, %	
Current smoker, %	40

© 2021 Mirshahi UL et al. *JAMA Network Open.*

eTable 3 Dictionary

Column Identifier	Definition
Chromosome	Chromosome number
Position	Genome coordinate (hg38)
Reference	Reference allele
Alternate	Alternate allele
Minor Allele Frequency	Minor allele frequency
Gene Names	Gene name
HGVS cDNA	Variant in Human Gene Variation Society (HGVS) DNA notation
HGVS protein	Variant in Human Gene Variation Society (HGVS) (protein) notation
Sequence Ontology	Variant type
Classification	Classifcation (pLOF: predicted loss of function, pDeleterious: predicted deleterious, VUS: variant of unknown significance, LB: likely beingn)
DiscovEHR MAF	Geisinger DiscovEHR minor allele frequency
ExAC nontcga NFE MAF	Exome Aggregation Consortium (ExAC), excluding The Cancer Genome Atlas (TCGA), non-Finnish European (NFE) minor allele frequency
gnomAD exome NFE MAF	Genome Aggregation Database (gnomAD) non-Finnish European minor allele frequency
MetaSVM pred	Meta-analytic support vector machine (MetaSVM) prediction score. D: deleterious; T: tolerated
CADD phred	Combined Annotation Dependent Depletion (CADD) phred-like score. This is phred-like rank score based on whole genome CADD raw scores.
REVEL	Rare Exome Variant Ensemble Learner (REVEL) score, an ensemble method for predicting the pathogenicity of rare missense variants
ClinVar Clinical significance (Last reviewed)	Clinical significance from ClinVar (last reviewed date)
ClinVar Review status	Review status from ClinVar
ClinVar Condition(s)	Clinvar clinical significance condition reported

© 2021 Mirshahi UL et al. *JAMA Network Open.*

eTable 3. List of all *DICER1* variants (P, LP, VUS, LB) and MAF in reference databases (ExAC_nonTCGA_NFE, gnomAD_exome_NFE), metaSVM, CADD, REVEL, HGMDand ClinVar

Chromosome	Position	Reference	Alternate	Gene	HGVS cDNA	HGVS protein	Sequence Ontology	Classification	DiscovEHR MAF	ExAC nontcga NFE MAF	gnomAD exome NFE MAF	MetaSVM pred	CADD phred	REVEL	ClinVar Clinical significance (Last reviewed)	ClinVar Allele ID	ClinVar Condition(s)	ClinVar Review status
14	95090516	A	G	DICER1	NM_177438.2:c.5751T>C	NP_803187.1:p.Pro1917=	synonymous	LB	5.42E-06	.	.	.	.	.	.	.	.	.
14	95090519	T	C	DICER1	NM_177438.2:c.5748A>G	NP_803187.1:p.Gln1916=	synonymous	LB	1.08E-05	1.84E-05	2.64E-05	.	.	.	Likely_benign (Last reviewed: Dec 31, 2019)	241898	Hereditary_cancer-predisposing_syndrome|not_provided	criteria_provided, multiple_submitters, no_conflicts
14	95090529	T	C	DICER1	NM_177438.2:c.5738A>G	NP_803187.1:p.Lys1913Arg	missense	LB	0.0001192	1.84E-05	2.64E-05	T	24.5	0.197	Uncertain_significance (Last reviewed: Jan 7, 2020)	331230	Pleuropulmonary_blastoma|Goiter,_multinodular_1,_with_or_without_Sertoli-Leydig_cell_tumors|Hereditary_cancer-predisposing_syndrome|Rhabdomyosarcoma,_embryonal,_2|DICER1-related_pleuropulmonary_blastoma_cancer_predisposition_syndrome	criteria_provided, multiple_submitters, no_conflicts
14	95090534	G	A	DICER1	NM_177438.2:c.5733C>T	NP_803187.1:p.Ser1911=	synonymous	LB	1.08E-05	.	8.79E-06	.	.	.	Likely_benign (Last reviewed: Oct 18, 2018)	784821	not_provided	criteria_provided, single_submitter
14	95090543	G	C	DICER1	NM_177438.2:c.5724C>G	NP_803187.1:p.Ala1908=	synonymous	LB	5.42E-06	.	.	.	.	.	.	.	.	.
14	95090589	A	G	DICER1	NM_177438.2:c.5678T>C	NP_803187.1:p.Val1893Ala	missense	LB	1.08E-05	.	.	T	27	0.266	Uncertain_significance (Last reviewed: Sep 25, 2019)	477295	Hereditary_cancer-predisposing_syndrome|DICER1-related_pleuropulmonary_blastoma_cancer_predisposition_syndrome	criteria_provided, multiple_submitters, no_conflicts
14	95090594	T	G	DICER1	NM_177438.2:c.5673A>C	NP_803187.1:p.Lys1891Asn	missense	LB	5.42E-06	.	.	T	24.7	0.126	.	.	.	.
14	95090620	C	T	DICER1	NM_177438.2:c.5647G>A	NP_803187.1:p.Glu1883Lys	missense	LB	5.42E-06	.	.	T	34	0.403	.	.	.	.
14	95090624	A	G	DICER1	NM_177438.2:c.5643T>C	NP_803187.1:p.Thr1881=	synonymous	LB	0.0002438	7.36E-05	0.0002	T	0.098	0.121	Likely_benign (Last reviewed: Dec 31, 2019)	222392	Hereditary_cancer-predisposing_syndrome|not_provided	criteria_provided, multiple_submitters, no_conflicts
14	95090645	G	T	DICER1	NM_177438.2:c.5622C>A	NP_803187.1:p.Tyr1874Ter	stop_gained	pLOF	5.42E-06	.	.	T	36	0.22	Uncertain_significance (Last reviewed: Nov 29, 2018)	642794	DICER1-related_pleuropulmonary_blastoma_cancer_predisposition_syndrome	criteria_provided, single_submitter
14	95090660	C	T	DICER1	NM_177438.2:c.5607G>A	NP_803187.1:p.Pro1869=	synonymous	LB	1.63E-05	7.36E-05	3.52E-05	T	16.81	0.03	Likely_benign (Last reviewed: Dec 31, 2019)	528861	Hereditary_cancer-predisposing_syndrome|not_provided	criteria_provided, multiple_submitters, no_conflicts
14	95090662	G	A	DICER1	NM_177438.2:c.5605C>T	NP_803187.1:p.Pro1869Ser	missense	pDeleterious	5.42E-06	.	.	D	24.8	0.516	.	.	.	.
14	95091081	G	T	DICER1	NM_177438.2:c.5556C>A	NP_803187.1:p.Ser1852=	synonymous	LB	5.42E-06	.	.	T	9.692	0.048	Likely_benign (Last reviewed: Dec 31, 2019)	399765	Hereditary_cancer-predisposing_syndrome|not_provided	criteria_provided, multiple_submitters, no_conflicts
14	95091085	C	T	DICER1	NM_177438.2:c.5552G>A	NP_803187.1:p.Arg1851His	missense	LB	3.25E-05	.	8.80E-06	T	35	0.161	Uncertain_significance (Last reviewed: Nov 25, 2019)	811919	Hereditary_cancer-predisposing_syndrome	criteria_provided, single_submitter
14	95091086	G	A	DICER1	NM_177438.2:c.5551C>T	NP_803187.1:p.Arg1851Cys	missense	LB	1.63E-05	.	.	T	35	0.416	Uncertain_significance (Last reviewed: Nov 26, 2019)	811920	Hereditary_cancer-predisposing_syndrome	criteria_provided, single_submitter
14	95091108	T	C	DICER1	NM_177438.2:c.5529A>G	NP_803187.1:p.Glu1843=	synonymous	LB	0.0001463	.	2.64E-05	T	5.748	0.056	Benign/Likely_benign (Last reviewed: Dec 31, 2019)	463601	Hereditary_cancer-predisposing_syndrome|not_provided	criteria_provided, multiple_submitters, no_conflicts
14	95091206	T	C	DICER1	NM_177438.2:c.5524A>G	NP_803187.1:p.Ile1842Val	missense	pDeleterious	2.71E-05	.	3.52E-05	D	23.8	0.448	Uncertain_significance (Last reviewed: Dec 15, 2019)	241901	Hereditary_cancer-predisposing_syndrome|DICER1-related_pleuropulmonary_blastoma_cancer_predisposition_syndrome	criteria_provided, multiple_submitters, no_conflicts
14	95091209	G	A	DICER1	NM_177438.2:c.5521C>T	NP_803187.1:p.Leu1841=	synonymous	LB	5.42E-06	.	.	.	.	.	Likely_benign (Last reviewed: Dec 31, 2019)	464199	Hereditary_cancer-predisposing_syndrome|not_provided	criteria_provided, multiple_submitters, no_conflicts
14	95091210	T	C	DICER1	NM_177438.2:c.5520A>G	NP_803187.1:p.Pro1840=	synonymous	LB	5.42E-06	.	.	.	.	.	Likely_benign (Last reviewed: May 22, 2016)	241902	not_provided	criteria_provided, single_submitter
14	95091214	C	T	DICER1	NM_177438.2:c.5516G>A	NP_803187.1:p.Arg1839Gln	missense	LB	3.79E-05	3.68E-05	7.04E-05	T	23	0.234	Uncertain_significance (Last reviewed: Dec 31, 2019)	137715	Hereditary_cancer-predisposing_syndrome|DICER1-related_pleuropulmonary_blastoma_cancer_predisposition_syndrome|not_specified	criteria_provided, multiple_submitters, no_conflicts
14	95091215	G	A	DICER1	NM_177438.2:c.5515C>T	NP_803187.1:p.Arg1839Trp	missense	LB	1.08E-05	.	8.80E-06	T	26.2	0.398	Uncertain_significance (Last reviewed: Dec 31, 2019)	477140	Hereditary_cancer-predisposing_syndrome|DICER1-related_pleuropulmonary_blastoma_cancer_predisposition_syndrome	criteria_provided, multiple_submitters, no_conflicts
14	95091240	G	C	DICER1	NM_177438.2:c.5490C>G	NP_803187.1:p.Val1830=	synonymous	LB	2.71E-05	.	.	.	.	.	.	.	.	.
14	95091255	TCCC	C	DICER1	NM_177438.2:c.5467_5475delinsG	NP_803187.1:p.Ser1823Valfs	frameshift	pLOF	5.42E-06	.	.	.	.	.	.	.	.	.
14	95091308	T	C	DICER1	NM_177438.2:c.5422A>G	NP_803187.1:p.Met1808Val	missense	LB	1.08E-05	.	8.79E-06	T	25.6	0.656	Uncertain_significance (Last reviewed: Feb 11, 2019)	464454	Hereditary_cancer-predisposing_syndrome|DICER1-related_pleuropulmonary_blastoma_cancer_predisposition_syndrome	criteria_provided, multiple_submitters, no_conflicts
14	95091361	C	T	DICER1	NM_177438.2:c.5369G>A	NP_803187.1:p.Arg1790Lys	missense	LB	5.42E-06	.	.	T	24.6	0.376	Uncertain_significance (Last reviewed: Nov 25, 2019)	568364	DICER1-related_pleuropulmonary_blastoma_cancer_predisposition_syndrome	criteria_provided, single_submitter
14	95091369	T	C	DICER1	NM_177438.2:c.5365-4A>G		splice_region	VUS	5.42E-06	.	.	.	.	.	.	.	.	.
14	95093909	T	C	DICER1	NM_177438.2:c.5343A>G	NP_803187.1:p.Glu1781=	synonymous	LB	5.42E-06	.	.	.	.	.	Likely_benign (Last reviewed: Dec 18, 2018)	811934	Hereditary_cancer-predisposing_syndrome	criteria_provided, single_submitter
14	95093915	C	T	DICER1	NM_177438.2:c.5337G>A	NP_803187.1:p.Lys1779=	synonymous	LB	2.71E-05	.	0	.	.	.	Benign/Likely_benign (Last reviewed: Dec 31, 2019)	241904	Hereditary_cancer-predisposing_syndrome|not_provided	criteria_provided, multiple_submitters, no_conflicts
14	95093922	A	T	DICER1	NM_177438.2:c.5330T>A	NP_803187.1:p.Leu1777His	missense	LB	5.42E-06	.	8.80E-06	T	20.9	0.383	Uncertain_significance (Last reviewed: Oct 9, 2019)	569087	Hereditary_cancer-predisposing_syndrome|DICER1-related_pleuropulmonary_blastoma_cancer_predisposition_syndrome	criteria_provided, multiple_submitters, no_conflicts

© 2021 Mirshahi UL et al. *JAMA Network Open.*

14	95093970	A	G	DICER1	NM_177438.2:c.5282T>C	NP_803187.1:p.Val1761Ala	missense	LB	5.42E-06	.	.	T	23.3	0.365	Uncertain_significance (Last reviewed: May 25, 2017)	477046	Hereditary_cancer-predisposing_syndrome	criteria_provided, single_submitter

© 2021 Mirshahi UL et al. *JAMA Network Open.*

14	95093976	T	C	DICER1	NM_177438.2:c.5276A>G	NP_803187.1:p.Lys1759Arg	missense	LB	9.21E-05	9.20E-05	9.67E-05	T	23.4	0.444	Uncertain_significance (Last reviewed: Jan 8, 2020)	137713	Hereditary_cancer-predisposing_syndrome|DICER1-related_pleuropulmonary_blastoma_cancer_predisposition_syndrome|not_specified	criteria_provided, multiple_submitters, no_conflicts
14	95093987	G	A	DICER1	NM_177438.2:c.5265C>T	NP_803187.1:p.His1755=	synonymous	LB	5.42E-06	.	.	.	.	.	.	.	.	.
14	95093995	C	T	DICER1	NM_177438.2:c.5257G>A	NP_803187.1:p.Asp1753Asn	missense	pDeleterious	1.08E-05	.	.	D	23.1	0.431	Uncertain_significance (Last reviewed: Dec 29, 2019)	464572	Hereditary_cancer-predisposing_syndrome|DICER1-related_pleuropulmonary_blastoma_cancer_predisposition_syndrome	criteria_provided, multiple_submitters, no_conflicts
14	95094049	G	A	DICER1	NM_177438.2:c.5203C>T	NP_803187.1:p.Leu1735=	synonymous	LB	5.42E-05	0.0007	0.0004	.	.	.	Benign/Likely_benign (Last reviewed: Dec 31, 2019)	241908	Hereditary_cancer-predisposing_syndrome|not_provided	criteria_provided, multiple_submitters, no_conflicts
14	95094062	C	T	DICER1	NM_177438.2:c.5190G>A	NP_803187.1:p.Gly1730=	synonymous	LB	5.42E-06	5.52E-05	2.64E-05	.	.	.	Likely_benign (Last reviewed: Dec 31, 2019)	464577	Hereditary_cancer-predisposing_syndrome|not_provided	criteria_provided, multiple_submitters, no_conflicts
14	95094065	C	T	DICER1	NM_177438.2:c.5187G>A	NP_803187.1:p.Pro1729=	synonymous	LB	1.63E-05	1.84E-05	8.80E-06	.	.	.	Likely_benign (Last reviewed: Dec 31, 2019)	400334	Hereditary_cancer-predisposing_syndrome|not_provided	criteria_provided, multiple_submitters, no_conflicts
14	95094079	G	A	DICER1	NM_177438.2:c.5173C>T	NP_803187.1:p.Arg1725Trp	missense	pDeleterious	1.08E-05	.	8.80E-06	D	34	0.861	Uncertain_significance (Last reviewed: Nov 19, 2018)	642806	DICER1-related_pleuropulmonary_blastoma_cancer_predisposition_syndrome	criteria_provided, single_submitter
14	95094080	C	T	DICER1	NM_177438.2:c.5172G>A	NP_803187.1:p.Pro1724=	synonymous	LB	5.42E-06	1.84E-05	8.80E-06	.	.	.	Likely_benign (Last reviewed: Mar 19, 2019)	739420	Hereditary_cancer-predisposing_syndrome|not_provided	criteria_provided, multiple_submitters, no_conflicts
14	95094089	A	G	DICER1	NM_177438.2:c.5163T>C	NP_803187.1:p.Tyr1721=	synonymous	LB	1.08E-05	.	0	.	.	.	Likely_benign (Last reviewed: Sep 3, 2018)	769970	not_provided	criteria_provided, single_submitter
14	95094095	G	A	DICER1	NM_177438.2:c.5157C>T	NP_803187.1:p.His1719=	synonymous	LB	5.42E-06	.	.	.	.	.	.	.	.	.
14	95094098	C	G	DICER1	NM_177438.2:c.5154G>C	NP_803187.1:p.Lys1718Asn	missense	LB	5.42E-06	.	.	T	26.7	0.395	Uncertain_significance (Last reviewed: Jun 11, 2019)	642807	Hereditary_cancer-predisposing_syndrome|DICER1-related_pleuropulmonary_blastoma_cancer_predisposition_syndrome	criteria_provided, multiple_submitters, no_conflicts
14	95094125	A	T	DICER1	NM_177438.2:c.5127T>A	NP_803187.1:p.Asp1709Glu	missense	pLOF	5.42E-06	.	.	D	25.5	0.888	.	.	.	.
14	95094142	A	C	DICER1	NM_177438.2:c.5110T>G	NP_803187.1:p.Leu1704Val	missense	pDeleterious	5.42E-06	.	.	D	21.6	0.826	.	.	.	.
14	95095829	G	A	DICER1	NM_177438.2:c.5091C>T	NP_803187.1:p.Ile1697=	synonymous	LB	5.42E-06	1.85E-05	3.52E-05	.	.	0.249	Likely_benign (Last reviewed: Dec 31, 2019)	477141	Hereditary_cancer-predisposing_syndrome|not_provided	criteria_provided, multiple_submitters, no_conflicts
14	95095831	T	C	DICER1	NM_177438.2:c.5089A>G	NP_803187.1:p.Ile1697Val	missense	LB	2.71E-05	.	8.81E-06	T	13.61	0.083	Uncertain_significance (Last reviewed: Feb 11, 2019)	464581	Hereditary_cancer-predisposing_syndrome|DICER1-related_pleuropulmonary_blastoma_cancer_predisposition_syndrome	criteria_provided, multiple_submitters, no_conflicts
14	95095878	G	A	DICER1	NM_177438.2:c.5042C>T	NP_803187.1:p.Ala1681Val	missense	pDeleterious	5.42E-06	.	.	D	29.3	0.692	.	.	.	.
14	95095907	C	A	DICER1	NM_177438.2:c.5013G>T	NP_803187.1:p.Lys1671Asn	missense	LB	5.42E-06	.	.	T	22.5	0.207	.	.	.	.
14	95095910	T	C	DICER1	NM_177438.2:c.5010A>G	NP_803187.1:p.Glu1670=	synonymous	LB	2.17E-05	.	.	.	.	0.139	Uncertain_significance (Last reviewed: Jun 14, 2016)	321923	Pleuropulmonary_blastoma	criteria_provided, single_submitter
14	95095916	AT	A	DICER1	NM_177438.2:c.5003_5004delinsT	NP_803187.1:p.Asn1668Ilefs	frameshift	pLOF	1.08E-05	.	.	.	.	.	.	.	.	.
14	95095920	T	C	DICER1	NM_177438.2:c.5000A>G	NP_803187.1:p.Glu1667Gly	missense	LB	5.42E-06	.	.	T	23.2	0.641	.	.	.	.
14	95095934	A	G	DICER1	NM_177438.2:c.4986T>C	NP_803187.1:p.Leu1662=	synonymous	LB	1.08E-05	3.68E-05	1.76E-05	.	.	0.059	Likely_benign (Last reviewed: Dec 31, 2019)	464216	Hereditary_cancer-predisposing_syndrome|not_provided	criteria_provided, multiple_submitters, no_conflicts
14	95095987	G	T	DICER1	NM_177438.2:c.4933C>A	NP_803187.1:p.Pro1645Thr	missense	LB	1.63E-05	.	.	T	24.3	0.139	.	.	.	.
14	95096009	C	T	DICER1	NM_177438.2:c.4911G>A	NP_803187.1:p.Ser1637=	synonymous	LB	7.04E-05	.	.	.	.	0.072	Likely_benign (Last reviewed: Dec 31, 2019)	688379	Hereditary_cancer-predisposing_syndrome|not_provided	criteria_provided, multiple_submitters, no_conflicts
14	95096010	G	A	DICER1	NM_177438.2:c.4910C>T	NP_803187.1:p.Ser1637Leu	missense	LB	0.0001517	1.84E-05	8.80E-06	T	12.28	0.065	Likely_benign (Last reviewed: Dec 31, 2019)	241913	Pleuropulmonary_blastoma|Hereditary_cancer-predisposing_syndrome|not_provided	criteria_provided, multiple_submitters, no_conflicts
14	95096019	A	G	DICER1	NM_177438.2:c.4901T>C	NP_803187.1:p.Leu1634Ser	missense	LB	8.13E-05	0.0001	0.0001	T	0.082	0.073	Conflicting_interpretations_of_pathogenicity (Last reviewed: Dec 31, 2019)	241914	Hereditary_cancer-predisposing_syndrome|not_provided	criteria_provided, conflicting_interpretations
14	95096021	T	C	DICER1	NM_177438.2:c.4899A>G	NP_803187.1:p.Val1633=	synonymous	LB	5.96E-05	.	4.40E-05	.	.	0.015	Likely_benign (Last reviewed: Dec 31, 2019)	241915	Hereditary_cancer-predisposing_syndrome|not_provided	criteria_provided, multiple_submitters, no_conflicts
14	95096024	A	G	DICER1	NM_177438.2:c.4896T>C	NP_803187.1:p.Ser1632=	synonymous	LB	4.33E-05	7.36E-05	4.40E-05	.	.	0.012	Likely_benign (Last reviewed: Dec 31, 2019)	400611	Hereditary_cancer-predisposing_syndrome|not_provided	criteria_provided, multiple_submitters, no_conflicts
14	95096031	C	T	DICER1	NM_177438.2:c.4889G>A	NP_803187.1:p.Arg1630His	missense	LB	1.08E-05	1.84E-05	0	T	19.31	0.03	Uncertain_significance (Last reviewed: Nov 23, 2019)	464217	Hereditary_cancer-predisposing_syndrome|DICER1-related_pleuropulmonary_blastoma_cancer_predisposition_syndrome	criteria_provided, multiple_submitters, no_conflicts
14	95096032	G	A	DICER1	NM_177438.2:c.4888C>T	NP_803187.1:p.Arg1630Cys	missense	LB	6.50E-05	.	8.80E-06	T	22.5	0.064	Uncertain_significance (Last reviewed: Dec 23, 2019)	399782	Hereditary_cancer-predisposing_syndrome|DICER1-related_pleuropulmonary_blastoma_cancer_predisposition_syndrome	criteria_provided, multiple_submitters, no_conflicts
14	95096046	G	T	DICER1	NM_177438.2:c.4874C>A	NP_803187.1:p.Ser1625Tyr	missense	LB	5.42E-06	.	8.80E-06	T	16.51	0.121	Uncertain_significance (Last reviewed: Feb 28, 2019)	222395	Hereditary_cancer-predisposing_syndrome|DICER1-related_pleuropulmonary_blastoma_cancer_predisposition_syndrome	criteria_provided, multiple_submitters, no_conflicts
14	95096050	C	G	DICER1	NM_177438.2:c.4870G>C	NP_803187.1:p.Ala1624Pro	missense	LB	2.17E-05	.	0	T	10.38	0.03	Uncertain_significance (Last reviewed: Jan 6, 2020)	477317	Ganglioneuroblastoma|Hereditary_cancer-predisposing_syndrome|DICER1-related_pleuropulmonary_blastoma_cancer_predisposition_syndrome	criteria_provided, multiple_submitters, no_conflicts
14	95096057	A	C	DICER1	NM_177438.2:c.4863T>G	NP_803187.1:p.Cys1621Trp	missense	LB	1.63E-05	.	.	T	0.87	0.141	.	.	.	.
14	95096061	C	T	DICER1	NM_177438.2:c.4859G>A	NP_803187.1:p.Ser1620Asn	missense	LB	2.71E-05	.	.	T	13.73	0.064	Uncertain_significance (Last reviewed: May 31, 2019)	464227	Hereditary_cancer-predisposing_syndrome|DICER1-related_pleuropulmonary_blastoma_cancer_predisposition_syndrome	criteria_provided, multiple_submitters, no_conflicts
14	95096081	T	C	DICER1	NM_177438.2:c.4839A>G	NP_803187.1:p.Gln1613=	synonymous	LB	1.08E-05	.	.	.	.	0.009	.	.	.	.
14	95096082	T	A	DICER1	NM_177438.2:c.4838A>T	NP_803187.1:p.Gln1613Leu	missense	pDeleterious	5.42E-06	.	8.80E-06	D	16.71	0.142	Uncertain_significance (Last reviewed: Dec 16, 2019)	477321	Hereditary_cancer-predisposing_syndrome	criteria_provided, single_submitter
14	95096101	G	A	DICER1	NM_177438.2:c.4819C>T	NP_803187.1:p.Arg1607Trp	missense	LB	6.50E-05	0.0001	0.0002	T	23.2	0.037	Conflicting_interpretations_of_pathogenicity (Last reviewed: Dec 29, 2019)	331238	Pleuropulmonary_blastoma|Hereditary_cancer-predisposing_syndrome|DICER1-related_pleuropulmonary_blastoma_cancer_predisposition_syndrome	criteria_provided, conflicting_interpretations

© 2021 Mirshahi UL et al. *JAMA Network Open.*

14	95096115	G	C	DICER1	NM_177438.2:c.4805C>G	NP_803187.1:p.Ala1602Gly	missense	LB	5.42E-06	.	0	T	6.834	0.101	Uncertain_significance (Last reviewed: Dec 18, 2019)	477674	Hereditary_cancer-predisposing_syndrome|DICER1-related_pleuropulmonary_blastoma_cancer_predisposition_syndrome	criteria_provided, multiple_submitters, no_conflicts
14	95096116	C	T	DICER1	NM_177438.2:c.4804G>A	NP_803187.1:p.Ala1602Thr	missense	LB	9.75E-05	0.0001	0.0001	T	12.15	0.015	Uncertain_significance (Last reviewed: Dec 11, 2019)	399792	Hereditary_cancer-predisposing_syndrome|DICER1-related_pleuropulmonary_blastoma_cancer_predisposition_syndrome	criteria_provided, multiple_submitters, no_conflicts
14	95096118	T	A	DICER1	NM_177438.2:c.4802A>T	NP_803187.1:p.Lys1601Met	missense	LB	0.0002113	0.0001	0.0001	T	12.43	0.035	Conflicting_interpretations_of_pathogenicity (Last reviewed: Dec 22, 2019)	241918	Pleuropulmonary_blastoma|Hereditary_cancer-predisposing_syndrome|DICER1-related_pleuropulmonary_blastoma_cancer_predisposition_syndrome	criteria_provided, conflicting_interpretations
14	95096124	C	T	DICER1	NM_177438.2:c.4796G>A	NP_803187.1:p.Arg1599Gln	missense	LB	5.42E-06	.	0	T	20.8	0.046	Conflicting_interpretations_of_pathogenicity (Last reviewed: Dec 31, 2019)	400340	Hereditary_cancer-predisposing_syndrome|not_provided	criteria_provided, conflicting_interpretations
14	95096129	A	G	DICER1	NM_177438.2:c.4791T>C	NP_803187.1:p.Thr1597=	synonymous	LB	2.17E-05	.	.	.	.	.	Likely_benign (Last reviewed: Apr 24, 2017)	477060	Hereditary_cancer-predisposing_syndrome	criteria_provided, single_submitter
14	95096146	G	A	DICER1	NM_177438.2:c.4774C>T	NP_803187.1:p.Pro1592Ser	missense	pDeleterious	5.42E-06	.	.	D	23.6	0.549	.	.	.	.
14	95096147	G	A	DICER1	NM_177438.2:c.4773C>T	NP_803187.1:p.Leu1591=	synonymous	LB	5.42E-06	.	0	.	.	0.117	Likely_benign (Last reviewed: Dec 31, 2019)	222396	Hereditary_cancer-predisposing_syndrome|not_specified|not_provided	criteria_provided, multiple_submitters, no_conflicts
14	95096171	G	C	DICER1	NM_177438.2:c.4749C>G	NP_803187.1:p.Leu1583=	synonymous	LB	1.08E-05	.	.	.	.	0.557	Likely_benign (Last reviewed: Jun 27, 2019)	464243	Hereditary_cancer-predisposing_syndrome|DICER1-related_pleuropulmonary_blastoma_cancer_predisposition_syndrome	criteria_provided, multiple_submitters, no_conflicts
14	95096180	C	A	DICER1	NM_177438.2:c.4740G>T	NP_803187.1:p.Gln1580His	missense	VUS	4.78E-05	5.52E-05	3.52E-05	D	25.9	0.101	Uncertain_significance (Last reviewed: Dec 15, 2019)	400617	Hereditary_cancer-predisposing_syndrome|DICER1-related_pleuropulmonary_blastoma_cancer_predisposition_syndrome	criteria_provided, multiple_submitters, no_conflicts
14	95096201	G	A	DICER1	NM_177438.2:c.4719C>T	NP_803187.1:p.Ser1573=	synonymous	LB	1.08E-05	.	0	.	.	.	Likely_benign (Last reviewed: May 23, 2019)	811973	Hereditary_cancer-predisposing_syndrome	criteria_provided, single_submitter
14	95096222	C	G	DICER1	NM_177438.2:c.4698G>C	NP_803187.1:p.Leu1566=	synonymous	LB	5.42E-06	.	0	.	.	0.05	Likely_benign (Last reviewed: Dec 31, 2019)	725875	Hereditary_cancer-predisposing_syndrome|not_provided	criteria_provided, multiple_submitters, no_conflicts
14	95096225	G	C	DICER1	NM_177438.2:c.4695C>G	NP_803187.1:p.Ala1565=	synonymous	LB	6.50E-05	.	.	.	.	0.061	Likely_benign (Last reviewed: Dec 31, 2019)	464251	not_provided	criteria_provided, single_submitter
14	95096255	A	G	DICER1	NM_177438.2:c.4665T>C	NP_803187.1:p.Ala1555=	synonymous	LB	5.42E-06	.	0	.	.	.	Likely_benign (Last reviewed: Dec 31, 2019)	463653	Hereditary_cancer-predisposing_syndrome|DICER1-related_pleuropulmonary_blastoma_cancer_predisposition_syndrome	criteria_provided, multiple_submitters, no_conflicts
14	95096268	T	C	DICER1	NM_177438.2:c.4652A>G	NP_803187.1:p.Glu1551Gly	missense	LB	5.42E-06	.	.	T	26.7	0.704	.	.	.	.
14	95096273	G	A	DICER1	NM_177438.2:c.4647C>T	NP_803187.1:p.His1549=	synonymous	LB	0.0002275	3.68E-05	0.0001	.	.	0.282	Likely_benign (Last reviewed: Dec 31, 2019)	400352	Hereditary_cancer-predisposing_syndrome|not_provided	criteria_provided, multiple_submitters, no_conflicts
14	95096276	C	G	DICER1	NM_177438.2:c.4644G>C	NP_803187.1:p.Leu1548Phe	missense	LB	5.42E-06	.	.	T	25.9	0.146	Uncertain_significance (Last reviewed: Nov 28, 2016)	400622	DICER1-related_pleuropulmonary_blastoma_cancer_predisposition_syndrome	criteria_provided, single_submitter
14	95096282	G	A	DICER1	NM_177438.2:c.4638C>T	NP_803187.1:p.Tyr1546=	synonymous	LB	5.96E-05	3.68E-05	4.40E-05	.	.	.	Likely_benign (Last reviewed: Dec 31, 2019)	399987	Hereditary_cancer-predisposing_syndrome|not_provided	criteria_provided, multiple_submitters, no_conflicts
14	95096291	G	A	DICER1	NM_177438.2:c.4629C>T	NP_803187.1:p.Ser1543=	synonymous	LB	5.42E-06	.	.	.	.	0.029	.	.	.	.
14	95096303	C	T	DICER1	NM_177438.2:c.4617G>A	NP_803187.1:p.Thr1539=	synonymous	LB	5.42E-06	.	0	.	.	0.048	Uncertain_significance (Last reviewed: Jul 14, 2016)	399798	DICER1-related_pleuropulmonary_blastoma_cancer_predisposition_syndrome	criteria_provided, single_submitter
14	95096318	G	A	DICER1	NM_177438.2:c.4602C>T	NP_803187.1:p.Asn1534=	synonymous	LB	5.42E-06	.	.	.	.	.	Likely_benign (Last reviewed: Dec 31, 2019)	477681	Hereditary_cancer-predisposing_syndrome|not_provided	criteria_provided, multiple_submitters, no_conflicts
14	95096327	T	G	DICER1	NM_177438.2:c.4593A>C	NP_803187.1:p.Ser1531=	synonymous	LB	5.42E-06	.	0	.	.	.	Likely_benign (Last reviewed: May 15, 2017)	477324	Hereditary_cancer-predisposing_syndrome	criteria_provided, single_submitter
14	95096366	A	T	DICER1	NM_177438.2:c.4554T>A	NP_803187.1:p.Val1518=	synonymous	LB	3.79E-05	0.0003	0.0002	.	.	.	Likely_benign (Last reviewed: Dec 31, 2019)	241923	Hereditary_cancer-predisposing_syndrome|not_provided	criteria_provided, multiple_submitters, no_conflicts
14	95096384	C	T	DICER1	NM_177438.2:c.4536G>A	NP_803187.1:p.Leu1512=	synonymous	LB	5.42E-06	.	.	.	.	0.052	.	.	.	.
14	95096388	T	C	DICER1	NM_177438.2:c.4532A>G	NP_803187.1:p.Tyr1511Cys	missense	pDeleterious	5.42E-06	.	.	D	25.8	0.539	.	.	.	.
14	95096405	A	G	DICER1	NM_177438.2:c.4515T>C	NP_803187.1:p.Ser1505=	synonymous	LB	0.0001192	.	0	.	.	.	Benign/Likely_benign (Last reviewed: Dec 31, 2019)	241924	Pleuropulmonary_blastoma|Hereditary_cancer-predisposing_syndrome|not_specified|not_provided	criteria_provided, multiple_submitters, no_conflicts
14	95096411	G	A	DICER1	NM_177438.2:c.4509C>T	NP_803187.1:p.Tyr1503=	synonymous	LB	1.63E-05	.	1.76E-05	.	.	.	Likely_benign (Last reviewed: Dec 31, 2019)	399802	Hereditary_cancer-predisposing_syndrome|not_provided	criteria_provided, multiple_submitters, no_conflicts
14	95096414	G	A	DICER1	NM_177438.2:c.4506C>T	NP_803187.1:p.Asp1502=	synonymous	LB	2.71E-05	.	.	.	.	.	Likely_benign (Last reviewed: Jan 26, 2018)	811980	Hereditary_cancer-predisposing_syndrome	criteria_provided, single_submitter
14	95096430	T	A	DICER1	NM_177438.2:c.4490A>T	NP_803187.1:p.Asp1497Val	missense	LB	2.17E-05	.	.	T	23.2	0.101	Uncertain_significance (Last reviewed: Aug 9, 2019)	642824	Hereditary_cancer-predisposing_syndrome|DICER1-related_pleuropulmonary_blastoma_cancer_predisposition_syndrome	criteria_provided, multiple_submitters, no_conflicts
14	95096431	C	T	DICER1	NM_177438.2:c.4489G>A	NP_803187.1:p.Asp1497Asn	missense	LB	1.08E-05	.	8.80E-06	T	22.3	0.123	Uncertain_significance (Last reviewed: Dec 27, 2018)	241925	Hereditary_cancer-predisposing_syndrome|DICER1-related_pleuropulmonary_blastoma_cancer_predisposition_syndrome	criteria_provided, multiple_submitters, no_conflicts
14	95096436	G	A	DICER1	NM_177438.2:c.4484C>T	NP_803187.1:p.Ser1495Leu	missense	LB	5.42E-06	.	0	T	21.5	0.171	Conflicting_interpretations_of_pathogenicity (Last reviewed: Dec 31, 2019)	463664	Hereditary_cancer-predisposing_syndrome|not_provided	criteria_provided, conflicting_interpretations
14	95096479	A	G	DICER1	NM_177438.2:c.4441T>C	NP_803187.1:p.Trp1481Arg	missense	pDeleterious	1.08E-05	.	8.80E-06	D	26.7	0.264	Uncertain_significance (Last reviewed: Dec 27, 2019)	811982	Hereditary_cancer-predisposing_syndrome	criteria_provided, single_submitter
14	95096497	T	C	DICER1	NM_177438.2:c.4423A>G	NP_803187.1:p.Thr1475Ala	missense	LB	1.08E-05	.	8.80E-06	T	0.28	0.058	Uncertain_significance (Last reviewed: Dec 4, 2019)	464256	Hereditary_cancer-predisposing_syndrome|DICER1-related_pleuropulmonary_blastoma_cancer_predisposition_syndrome	criteria_provided, multiple_submitters, no_conflicts

© 2021 Mirshahi UL et al. *JAMA Network Open.*

14	95096508	G	A	DICER1	NM_177438.2:c.4412C>T	NP_803187.1:p.Pro1471Leu	missense	LB	2.17E-05	.	8.80E-06	T	22.6	0.04	Uncertain_significance (Last reviewed: Dec 15, 2019)	399807	Hereditary_cancer-predisposing_syndrome|DICER1-related_pleuropulmonary_blastoma_cancer_predisposition_syndrome	criteria_provided, multiple_submitters, no_conflicts
14	95096513	A	G	DICER1	NM_177438.2:c.4407T>C	NP_803187.1:p.Leu1469=	synonymous	LB	2.71E-05	.	0	.	.	0.093	Benign/Likely_benign (Last reviewed: Dec 31, 2019)	255118	Hereditary_cancer-predisposing_syndrome|not_specified|not_provided	criteria_provided, multiple_submitters, no_conflicts
14	95096514	A	G	DICER1	NM_177438.2:c.4406T>C	NP_803187.1:p.Leu1469Pro	missense	pDeleterious	2.71E-05	.	0	D	26.5	0.525	Uncertain_significance (Last reviewed: Oct 31, 2018)	464608	Hereditary_cancer-predisposing_syndrome|DICER1-related_pleuropulmonary_blastoma_cancer_predisposition_syndrome	criteria_provided, multiple_submitters, no_conflicts
14	95096515	G	T	DICER1	NM_177438.2:c.4405C>A	NP_803187.1:p.Leu1469Ile	missense	LB	3.25E-05	.	0	T	23.9	0.197	Uncertain_significance (Last reviewed: Apr 28, 2019)	400628	Hereditary_cancer-predisposing_syndrome|DICER1-related_pleuropulmonary_blastoma_cancer_predisposition_syndrome	criteria_provided, multiple_submitters, no_conflicts
14	95096517	G	C	DICER1	NM_177438.2:c.4403C>G	NP_803187.1:p.Ser1468Cys	missense	pDeleterious	5.42E-06	.	0	D	26.1	0.24	Uncertain_significance (Last reviewed: Nov 5, 2019)	477078	Hereditary_cancer-predisposing_syndrome|DICER1-related_pleuropulmonary_blastoma_cancer_predisposition_syndrome	criteria_provided, multiple_submitters, no_conflicts
14	95096525	C	G	DICER1	NM_177438.2:c.4395G>C	NP_803187.1:p.Lys1465Asn	missense	pDeleterious	1.63E-05	.	.	D	26.8	0.133	.	.	.	.
14	95096541	G	A	DICER1	NM_177438.2:c.4379C>T	NP_803187.1:p.Ser1460Leu	missense	pDeleterious	5.42E-06	.	.	D	24.2	0.512	Uncertain_significance (Last reviewed: Dec 27, 2018)	642827	DICER1-related_pleuropulmonary_blastoma_cancer_predisposition_syndrome	criteria_provided, single_submitter
14	95096554	T	C	DICER1	NM_177438.2:c.4366A>G	NP_803187.1:p.Met1456Val	missense	LB	5.42E-06	.	.	T	20.7	0.129	Uncertain_significance (Last reviewed: Jul 27, 2018)	642830	DICER1-related_pleuropulmonary_blastoma_cancer_predisposition_syndrome	criteria_provided, single_submitter
14	95096556	T	G	DICER1	NM_177438.2:c.4364A>C	NP_803187.1:p.Asn1455Thr	missense	LB	5.42E-06	.	.	T	20.6	0.073	Uncertain_significance (Last reviewed: Mar 7, 2019)	241928	DICER1-related_pleuropulmonary blastoma cancer predisposition_syndrome	criteria_provided, single_submitter
14	95096559	T	C	DICER1	NM_177438.2:c.4361A>G	NP_803187.1:p.Asp1454Gly	missense	pDeleterious	1.08E-05	.	.	D	25.9	0.587	.	.	.	.
14	95096563	T	C	DICER1	NM_177438.2:c.4357A>G	NP_803187.1:p.Ile1453Val	missense	LB	1.63E-05	.	0	T	24.8	0.316	.	.	.	.
14	95096572	T	C	DICER1	NM_177438.2:c.4348A>G	NP_803187.1:p.Ile1450Val	missense	LB	1.63E-05	.	.	T	24.7	0.296	.	.	.	.
14	95096584	C	T	DICER1	NM_177438.2:c.4336G>A	NP_803187.1:p.Asp1446Asn	missense	pDeleterious	5.42E-06	.	.	D	23.1	0.169	Uncertain_significance (Last reviewed: Aug 20, 2018)	642831	DICER1-related_pleuropulmonary_blastoma_cancer_predisposition_syndrome	criteria_provided, single_submitter
14	95096593	G	A	DICER1	NM_177438.2:c.4327C>T	NP_803187.1:p.Leu1443=	synonymous	LB	4.33E-05	.	.	.	.	0.227	Likely_benign (Last reviewed: Dec 31, 2019)	399994	DICER1-related_pleuropulmonary_blastoma_cancer_predisposition_syndrome	criteria_provided, single_submitter
14	95096608	A	T	DICER1	NM_177438.2:c.4312T>A	NP_803187.1:p.Tyr1438Asn	missense	LB	1.08E-05	.	.	T	0.115	0.111	Uncertain_significance (Last reviewed: Mar 31, 2019)	241929	DICER1-related_pleuropulmonary_blastoma_cancer_predisposition_syndrome	criteria_provided, single_submitter
14	95096624	C	T	DICER1	NM_177438.2:c.4296G>A	NP_803187.1:p.Pro1432=	synonymous	LB	5.42E-06	.	0	.	.	0.236	Likely_benign (Last reviewed: Dec 31, 2019)	82748	Hereditary_cancer-predisposing_syndrome|not_provided	criteria_provided, multiple_submitters, no_conflicts
14	95096625	G	A	DICER1	NM_177438.2:c.4295C>T	NP_803187.1:p.Pro1432Leu	missense	pDeleterious	5.42E-06	7.42E-05	2.71E-05	D	26.4	0.243	Uncertain_significance (Last reviewed: Dec 20, 2019)	464610	DICER1-related_pleuropulmonary_blastoma_cancer_predisposition_syndrome	criteria_provided, single_submitter
14	95096636	C	T	DICER1	NM_177438.2:c.4284G>A	NP_803187.1:p.Met1428Ile	missense	LB	5.42E-06	.	.	T	17.03	0.06	.	.	.	.
14	95096641	G	A	DICER1	NM_177438.2:c.4279C>T	NP_803187.1:p.Leu1427=	synonymous	LB	5.42E-06	.	.	.	.	0.023	.	.	.	.
14	95096654	C	A	DICER1	NM_177438.2:c.4266G>T	NP_803187.1:p.Glu1422Asp	missense	LB	5.42E-06	.	9.07E-06	T	6.807	0.067	Uncertain_significance (Last reviewed: Feb 14, 2018)	464261	Hereditary_cancer-predisposing_syndrome|DICER1-related_pleuropulmonary_blastoma_cancer_predisposition_syndrome	criteria_provided, multiple_submitters, no_conflicts
14	95096664	T	A	DICER1	NM_177438.2:c.4256A>T	NP_803187.1:p.Glu1419Val	missense	LB	5.42E-06	.	0	T	15.39	0.082	Uncertain_significance (Last reviewed: Oct 30, 2019)	573013	Hereditary_cancer-predisposing_syndrome|DICER1-related_pleuropulmonary_blastoma_cancer_predisposition_syndrome	criteria_provided, multiple_submitters, no_conflicts
14	95096669	G	A	DICER1	NM_177438.2:c.4251C>T	NP_803187.1:p.Tyr1417=	synonymous	LB	2.17E-05	.	9.20E-06	.	.	.	Likely_benign (Last reviewed: Dec 31, 2019)	528888	Hereditary_cancer-predisposing_syndrome|not_provided	criteria_provided, multiple_submitters, no_conflicts
14	95096675	C	T	DICER1	NM_177438.2:c.4245G>A	NP_803187.1:p.Glu1415=	synonymous	LB	5.42E-06	2.18E-05	1.84E-05	.	.	0.014	Likely_benign (Last reviewed: Dec 31, 2019)	464615	Hereditary_cancer-predisposing_syndrome|not_provided	criteria_provided, multiple_submitters, no_conflicts
14	95096690	A	G	DICER1	NM_177438.2:c.4230T>C	NP_803187.1:p.Asn1410=	synonymous	LB	5.42E-06	.	.	.	.	0.117	Likely_benign (Last reviewed: Dec 31, 2019)	400009	Hereditary_cancer-predisposing_syndrome|not_provided	criteria_provided, multiple_submitters, no_conflicts
14	95096694	G	A	DICER1	NM_177438.2:c.4226C>T	NP_803187.1:p.Ala1409Val	missense	LB	2.17E-05	.	.	T	23.1	0.05	.	.	.	.
14	95096695	C	A	DICER1	NM_177438.2:c.4225G>T	NP_803187.1:p.Ala1409Ser	missense	LB	5.42E-06	.	.	T	19.48	0.065	.	.	.	.
14	95096704	A	C	DICER1	NM_177438.2:c.4216T>G	NP_803187.1:p.Cys1406Gly	missense	LB	1.63E-05	.	.	T	6.783	0.035	Uncertain_significance (Last reviewed: Jul 30, 2019)	642840	Hereditary_cancer-predisposing_syndrome|DICER1-related_pleuropulmonary_blastoma_cancer_predisposition_syndrome	criteria_provided, multiple_submitters, no_conflicts
14	95096711	T	A	DICER1	NM_177438.2:c.4209A>T	NP_803187.1:p.Thr1403=	synonymous	LB	1.63E-05	.	.	.	.	.	Likely_benign (Last reviewed: Dec 31, 2019)	754230	not_provided	criteria_provided, single_submitter
14	95096718	A	C	DICER1	NM_177438.2:c.4207-5T>G		splice_region	VUS	5.42E-06	.	.	.	.	.	Uncertain_significance (Last reviewed: Jan 15, 2018)	815589	Hereditary_cancer-predisposing_syndrome	criteria_provided, single_submitter
14	95099787	T	C	DICER1	NM_177438.2:c.4199A>G	NP_803187.1:p.Asp1400Gly	missense	LB	9.21E-05	9.22E-05	4.40E-05	T	22.8	0.142	Uncertain_significance (Last reviewed: Dec 30, 2019)	400356	Hereditary_cancer-predisposing_syndrome|DICER1-related_pleuropulmonary_blastoma_cancer_predisposition_syndrome|not_provided	criteria_provided, multiple_submitters, no_conflicts
14	95099805	G	GTGT	DICER1	NM_177438.2:c.4181delinsACAC	NP_803187.1:p.Asn1393_Thr1394insAsn	inframe_insertion	VUS	5.96E-05	.	.	.	.	.	.	.	.	.
14	95099875	C	G	DICER1	NM_177438.2:c.4111G>C	NP_803187.1:p.Val1371Leu	missense	LB	5.42E-06	.	.	T	32	0.425	.	.	.	.
14	95099882	G	T	DICER1	NM_177438.2:c.4104C>A	NP_803187.1:p.Arg1368=	synonymous	LB	0.0001463	1.84E-05	8.80E-06	.	.	0.046	Likely_benign (Last reviewed: Dec 31, 2019)	477082	Hereditary_cancer-predisposing_syndrome|not_provided	criteria_provided, multiple_submitters, no_conflicts
14	95099884	G	A	DICER1	NM_177438.2:c.4102C>T	NP_803187.1:p.Arg1368Cys	missense	LB	1.63E-05	3.68E-05	4.40E-05	T	30	0.077	Uncertain_significance (Last reviewed: Dec 11, 2019)	241937	Hereditary_cancer-predisposing_syndrome|DICER1-related_pleuropulmonary_blastoma_cancer_predisposition_syndrome	criteria_provided, multiple_submitters, no_conflicts

© 2021 Mirshahi UL et al. *JAMA Network Open.*

14	95099885	G	A	DICER1	NM_177438.2:c.4101C>T	NP_803187.1:p.Ser1367=	synonymous	LB	5.42E-06	.	0	.	.	0.041	Likely_benign (Last reviewed: Dec 31, 2019)	399813	Hereditary_cancer-predisposing_syndrome|not_provided	criteria_provided, multiple_submitters, no_conflicts

© 2021 Mirshahi UL et al. *JAMA Network Open.*

14	95099895	C	G	DICER1	NM_177438.2:c.4091G>C	NP_803187.1:p.Gly1364Ala	missense	pDeleterious	2.17E-05	.	1.76E-05	D	20.1	0.684	Uncertain_significance (Last reviewed: Aug 18, 2019)	464634	Hereditary_cancer-predisposing_syndrome|DICER1-related_pleuropulmonary_blastoma_cancer_predisposition_syndrome	criteria_provided, multiple_submitters, no_conflicts
14	95099913	C	T	DICER1	NM_177438.2:c.4073G>A	NP_803187.1:p.Arg1358His	missense	LB	5.42E-06	1.84E-05	1.76E-05	T	34	0.649	Uncertain_significance (Last reviewed: Sep 14, 2019)	400361	Hereditary_cancer-predisposing_syndrome|DICER1-related_pleuropulmonary_blastoma_cancer_predisposition_syndrome	criteria_provided, multiple_submitters, no_conflicts
14	95099914	G	A	DICER1	NM_177438.2:c.4072C>T	NP_803187.1:p.Arg1358Cys	missense	LB	5.42E-06	.	0	T	27.9	0.161	Uncertain_significance (Last reviewed: Nov 13, 2019)	477084	Hereditary_cancer-predisposing_syndrome	criteria_provided, single_submitter
14	95099926	A	T	DICER1	NM_177438.2:c.4060T>A	NP_803187.1:p.Cys1354Ser	missense	LB	5.42E-06	.	2.64E-05	T	23	0.309	Uncertain_significance (Last reviewed: Sep 30, 2019)	464644	Hereditary_cancer-predisposing_syndrome|DICER1-related_pleuropulmonary_blastoma_cancer_predisposition_syndrome	criteria_provided, multiple_submitters, no_conflicts
14	95103345	C	T	DICER1	NM_177438.2:c.4050+1G>A		splice_donor	pLOF	5.42E-06	.	.	.	25.6	.	Pathogenic/Likely_pathogenic (Last reviewed: Jul 15, 2014)	227700	Pineoblastoma|Hereditary_cancer-predisposing_syndrome|not_provided	criteria_provided, multiple_submitters, no_conflicts
14	95103362	T	C	DICER1	NM_177438.2:c.4034A>G	NP_803187.1:p.Tyr1345Cys	missense	pDeleterious	1.08E-05	.	.	D	25.8	0.602	.	.	.	.
14	95103372	G	A	DICER1	NM_177438.2:c.4024C>T	NP_803187.1:p.Arg1342Cys	missense	pDeleterious	5.42E-06	.	.	D	34	0.103	Uncertain_significance (Last reviewed: Oct 23, 2019)	400367	Hereditary_cancer-predisposing_syndrome|DICER1-related_pleuropulmonary_blastoma_cancer_predisposition_syndrome	criteria_provided, multiple_submitters, no_conflicts
14	95103379	A	G	DICER1	NM_177438.2:c.4017T>C	NP_803187.1:p.His1339=	synonymous	LB	2.71E-05	.	0	.	.	.	Likely_benign (Last reviewed: Dec 31, 2019)	754231	Hereditary_cancer-predisposing_syndrome|not_provided	criteria_provided, multiple_submitters, no_conflicts
14	95103383	G	A	DICER1	NM_177438.2:c.4013C>T	NP_803187.1:p.Ala1338Val	missense	LB	5.42E-06	3.69E-05	1.76E-05	T	23.5	0.454	Uncertain_significance (Last reviewed: Jul 4, 2019)	463696	Hereditary_cancer-predisposing_syndrome|DICER1-related_pleuropulmonary_blastoma_cancer_predisposition_syndrome	criteria_provided, multiple_submitters, no_conflicts
14	95103398	C	A	DICER1	NM_177438.2:c.3998G>T	NP_803187.1:p.Cys1333Phe	missense	LB	5.42E-06	.	.	T	29.4	0.288	.	.	.	.
14	95103403	T	C	DICER1	NM_177438.2:c.3993A>G	NP_803187.1:p.Leu1331=	synonymous	LB	1.08E-05	3.68E-05	6.16E-05	.	.	0.142	Likely_benign (Last reviewed: Dec 31, 2019)	528858	Hereditary_cancer-predisposing_syndrome|not_provided	criteria_provided, multiple_submitters, no_conflicts
14	95103409	T	A	DICER1	NM_177438.2:c.3987A>T	NP_803187.1:p.Thr1329=	synonymous	LB	5.42E-06	.	.	.	.	0.031	.	.	.	.
14	95103412	G	T	DICER1	NM_177438.2:c.3984C>A	NP_803187.1:p.Thr1328=	synonymous	LB	5.42E-06	.	.	.	.	0.05	.	.	.	.
14	95103439	G	A	DICER1	NM_177438.2:c.3957C>T	NP_803187.1:p.Gly1319=	synonymous	LB	1.63E-05	7.36E-05	4.40E-05	.	.	.	Conflicting_interpretations_of_pathogenicity (Last reviewed: Nov 29, 2018)	241940	Hereditary_cancer-predisposing_syndrome|DICER1-related_pleuropulmonary_blastoma_cancer_predisposition_syndrome|not_provided	criteria_provided, conflicting_interpretations
14	95103455	C	T	DICER1	NM_177438.2:c.3941G>A	NP_803187.1:p.Arg1314Gln	missense	pDeleterious	5.42E-06	.	.	D	34	0.908	.	.	.	.
14	95103457	C	T	DICER1	NM_177438.2:c.3939G>A	NP_803187.1:p.Glu1313=	synonymous	LB	4.88E-05	.	0	.	.	.	Likely_benign (Last reviewed: Dec 31, 2019)	400372	Hereditary_cancer-predisposing_syndrome|not_provided	criteria_provided, multiple_submitters, no_conflicts
14	95103481	G	A	DICER1	NM_177438.2:c.3915C>T	NP_803187.1:p.Asn1305=	synonymous	LB	3.25E-05	.	1.76E-05	.	.	.	Likely_benign (Last reviewed: Dec 31, 2019)	241941	Hereditary_cancer-predisposing_syndrome|not_provided	criteria_provided, multiple_submitters, no_conflicts
14	95103510	G	C	DICER1	NM_177438.2:c.3886C>G	NP_803187.1:p.Leu1296Val	missense	pDeleterious	5.42E-06	.	.	D	22.6	0.574	.	.	.	.
14	95103518	T	C	DICER1	NM_177438.2:c.3878A>G	NP_803187.1:p.Asn1293Ser	missense	LB	1.08E-05	1.84E-05	8.80E-06	T	13.75	0.362	Uncertain_significance (Last reviewed: Dec 20, 2019)	464557	Hereditary_cancer-predisposing_syndrome|DICER1-related_pleuropulmonary_blastoma_cancer_predisposition_syndrome	criteria_provided, multiple_submitters, no_conflicts
14	95103540	A	C	DICER1	NM_177438.2:c.3856T>G	NP_803187.1:p.Ser1286Ala	missense	LB	5.42E-06	.	.	T	10.41	0.218	.	.	.	.
14	95103545	C	G	DICER1	NM_177438.2:c.3851G>C	NP_803187.1:p.Gly1284Ala	missense	LB	5.42E-06	.	0	T	11.01	0.024	Conflicting_interpretations_of_pathogenicity (Last reviewed: Dec 31, 2019)	477099	Hereditary_cancer-predisposing_syndrome|not_provided	criteria_provided, conflicting_interpretations
14	95103548	A	G	DICER1	NM_177438.2:c.3848T>C	NP_803187.1:p.Ile1283Thr	missense	LB	5.42E-06	.	0	T	0.062	0.041	Uncertain_significance (Last reviewed: Feb 7, 2018)	241943	Hereditary_cancer-predisposing_syndrome|DICER1-related_pleuropulmonary_blastoma_cancer_predisposition_syndrome	criteria_provided, multiple_submitters, no_conflicts
14	95103554	G	A	DICER1	NM_177438.2:c.3842C>T	NP_803187.1:p.Pro1281Leu	missense	LB	5.42E-06	.	0	T	18.74	0.062	Uncertain_significance (Last reviewed: Feb 2, 2017)	464314	DICER1-related_pleuropulmonary_blastoma_cancer_predisposition_syndrome	criteria_provided, single_submitter
14	95103573	T	C	DICER1	NM_177438.2:c.3823A>G	NP_803187.1:p.Met1275Val	missense	LB	2.17E-05	3.68E-05	4.40E-05	T	0.001	0.116	Uncertain_significance (Last reviewed: Dec 27, 2019)	241944	Hereditary_cancer-predisposing_syndrome|DICER1-related_pleuropulmonary_blastoma_cancer_predisposition_syndrome	criteria_provided, multiple_submitters, no_conflicts
14	95103576	T	G	DICER1	NM_177438.2:c.3820A>C	NP_803187.1:p.Arg1274=	synonymous	LB	2.71E-05	.	0	.	.	.	Likely_benign (Last reviewed: Dec 31, 2019)	400651	Hereditary_cancer-predisposing_syndrome|not_provided	criteria_provided, multiple_submitters, no_conflicts
14	95103581	T	C	DICER1	NM_177438.2:c.3815A>G	NP_803187.1:p.Lys1272Arg	missense	LB	1.63E-05	.	8.80E-06	T	0.091	0.059	Uncertain_significance (Last reviewed: Dec 3, 2019)	463697	Hereditary_cancer-predisposing_syndrome|DICER1-related_pleuropulmonary_blastoma_cancer_predisposition_syndrome	criteria_provided, multiple_submitters, no_conflicts
14	95103585	G	A	DICER1	NM_177438.2:c.3811C>T	NP_803187.1:p.Leu1271Phe	missense	LB	5.42E-06	.	0	T	7.681	0.057	Uncertain_significance (Last reviewed: Dec 16, 2019)	573023	Hereditary_cancer-predisposing_syndrome|DICER1-related_pleuropulmonary_blastoma_cancer_predisposition_syndrome	criteria_provided, multiple_submitters, no_conflicts
14	95103598	G	A	DICER1	NM_177438.2:c.3798C>T	NP_803187.1:p.Asp1266=	synonymous	LB	5.42E-06	.	.	.	.	.	.	.	.	.
14	95103604	C	T	DICER1	NM_177438.2:c.3792G>A	NP_803187.1:p.Thr1264=	synonymous	LB	9.75E-05	0.0002	0.0002	.	.	.	Likely_benign (Last reviewed: Dec 31, 2019)	241945	Hereditary_cancer-predisposing_syndrome|not_provided	criteria_provided, multiple_submitters, no_conflicts
14	95103605	G	A	DICER1	NM_177438.2:c.3791C>T	NP_803187.1:p.Thr1264Met	missense	LB	5.96E-05	1.84E-05	2.64E-05	T	20.4	0.039	Uncertain_significance (Last reviewed: Dec 31, 2019)	400377	Hereditary_cancer-predisposing_syndrome|DICER1-related_pleuropulmonary_blastoma_cancer_predisposition_syndrome	criteria_provided, multiple_submitters, no_conflicts
14	95103618	C	T	DICER1	NM_177438.2:c.3778G>A	NP_803187.1:p.Val1260Ile	missense	LB	5.42E-05	.	1.76E-05	T	0.1	0.023	Uncertain_significance (Last reviewed: Dec 19, 2019)	400378	Hereditary_cancer-predisposing_syndrome|DICER1-related_pleuropulmonary_blastoma_cancer_predisposition_syndrome	criteria_provided, multiple_submitters, no_conflicts

© 2021 Mirshahi UL et al. *JAMA Network Open.*

14	95103650	T	C	DICER1	NM_177438.2:c.3746A>G	NP_803187.1:p.Lys1249Arg	missense	LB	5.42E-06	.	8.80E-06	T	4.401	0.148	Uncertain_significance (Last reviewed: Jun 27, 2019)	399826	Hereditary_cancer-predisposing_syndrome|DICER1-related_pleuropulmonary_blastoma_cancer_predisposition_syndrome	criteria_provided, multiple_submitters, no_conflicts
14	95103659	T	C	DICER1	NM_177438.2:c.3737A>G	NP_803187.1:p.Asn1246Ser	missense	LB	5.42E-06	.	.	T	12.1	0.061	.	.	.	.
14	95103664	A	G	DICER1	NM_177438.2:c.3732T>C	NP_803187.1:p.Asp1244=	synonymous	LB	5.42E-06	1.84E-05	8.80E-06	.	.	.	Likely_benign (Last reviewed: Dec 31, 2019)	754232	not_provided	criteria_provided, single_submitter
14	95103674	T	C	DICER1	NM_177438.2:c.3722A>G	NP_803187.1:p.Lys1241Arg	missense	LB	2.71E-05	1.84E-05	3.52E-05	T	7.886	0.025	Uncertain_significance (Last reviewed: Dec 20, 2019)	464570	Goiter,_multinodular_1,_with_or_without_Sertoli-Leydig_cell_tumors|Hereditary_cancer-predisposing_syndrome|Rhabdomyosarcoma,_embryonal,_2|DICER1-related_pleuropulmonary_blastoma_cancer_predisposition_syndrome|not_provided	criteria_provided, multiple_submitters, no_conflicts
14	95103682	C	G	DICER1	NM_177438.2:c.3714G>C	NP_803187.1:p.Leu1238=	synonymous	LB	5.42E-06	.	.	.	.	.	.	.	.	.
14	95103683	A	G	DICER1	NM_177438.2:c.3713T>C	NP_803187.1:p.Leu1238Pro	missense	LB	1.08E-05	.	.	T	12.17	0.08	Uncertain_significance (Last reviewed: Oct 3, 2018)	400652	Hereditary_cancer-predisposing_syndrome|DICER1-related_pleuropulmonary_blastoma_cancer_predisposition_syndrome	criteria_provided, multiple_submitters, no_conflicts
14	95103695	T	G	DICER1	NM_177438.2:c.3701A>C	NP_803187.1:p.Glu1234Ala	missense	LB	5.42E-06	.	.	T	17.86	0.173	.	.	.	.
14	95103698	T	C	DICER1	NM_177438.2:c.3698A>G	NP_803187.1:p.Asp1233Gly	missense	LB	6.50E-05	.	.	T	17.33	0.09	Uncertain_significance (Last reviewed: Nov 8, 2018)	642849	DICER1-related_pleuropulmonary_blastoma_cancer_predisposition_syndrome	criteria_provided, single_submitter
14	95103719	T	G	DICER1	NM_177438.2:c.3677A>C	NP_803187.1:p.Glu1226Ala	missense	LB	3.25E-05	5.52E-05	2.64E-05	T	10.5	0.058	Uncertain_significance (Last reviewed: Nov 25, 2019)	477339	Hereditary_cancer-predisposing_syndrome|DICER1-related_pleuropulmonary_blastoma_cancer_predisposition_syndrome	criteria_provided, multiple_submitters, no_conflicts
14	95103722	T	C	DICER1	NM_177438.2:c.3674A>G	NP_803187.1:p.Tyr1225Cys	missense	LB	1.63E-05	5.52E-05	3.52E-05	T	1.792	0.022	Conflicting_interpretations_of_pathogenicity (Last reviewed: Dec 17, 2019)	241946	Pleuropulmonary_blastoma|Hereditary_cancer-predisposing_syndrome|DICER1-related_pleuropulmonary_blastoma_cancer_predisposition_syndrome	criteria_provided, conflicting_interpretations
14	95103742	G	C	DICER1	NM_177438.2:c.3654C>G	NP_803187.1:p.Ser1218=	synonymous	LB	5.42E-06	1.84E-05	8.79E-06	.	.	.	Likely_benign (Last reviewed: Dec 31, 2019)	725876	Hereditary_cancer-predisposing_syndrome|not_provided	criteria_provided, multiple_submitters, no_conflicts
14	95103756	T	C	DICER1	NM_177438.2:c.3640A>G	NP_803187.1:p.Thr1214Ala	missense	LB	1.08E-05	.	2.64E-05	T	0.887	0.059	Uncertain_significance (Last reviewed: Sep 27, 2019)	463702	Hereditary_cancer-predisposing_syndrome|DICER1-related_pleuropulmonary_blastoma_cancer_predisposition_syndrome	criteria_provided, multiple_submitters, no_conflicts
14	95103759	G	C	DICER1	NM_177438.2:c.3637C>G	NP_803187.1:p.Pro1213Ala	missense	LB	2.17E-05	.	2.64E-05	T	19.26	0.053	Uncertain_significance (Last reviewed: Jan 15, 2018)	464322	Hereditary_cancer-predisposing_syndrome|DICER1-related_pleuropulmonary_blastoma_cancer_predisposition_syndrome	criteria_provided, multiple_submitters, no_conflicts
14	95103780	TGTA	T	DICER1	NM_177438.2:c.3613_3616delinsA	NP_803187.1:p.Tyr1205del	inframe_deletion	VUS	1.08E-05	.	8.80E-06	.	.	.	.	.	.	.
14	95103797	T	C	DICER1	NM_177438.2:c.3599A>G	NP_803187.1:p.Asn1200Ser	missense	LB	5.42E-06	.	.	T	7.464	0.126	Uncertain_significance (Last reviewed: Sep 28, 2019)	812030	Hereditary_cancer-predisposing_syndrome	criteria_provided, single_submitter
14	95103829	A	G	DICER1	NM_177438.2:c.3567T>C	NP_803187.1:p.Tyr1189=	synonymous	LB	5.42E-06	.	0	.	.	.	Likely_benign (Last reviewed: Dec 31, 2019)	464660	Hereditary_cancer-predisposing_syndrome|not_provided	criteria_provided, multiple_submitters, no_conflicts
14	95103832	A	C	DICER1	NM_177438.2:c.3564T>G	NP_803187.1:p.Ser1188Arg	missense	LB	5.42E-06	.	.	T	10.37	0.051	.	.	.	.
14	95103839	T	C	DICER1	NM_177438.2:c.3557A>G	NP_803187.1:p.Asn1186Ser	missense	LB	1.08E-05	.	.	T	10.82	0.106	Uncertain_significance (Last reviewed: Dec 18, 2018)	463709	Hereditary_cancer-predisposing_syndrome|DICER1-related_pleuropulmonary_blastoma_cancer_predisposition_syndrome	criteria_provided, multiple_submitters, no_conflicts
14	95103843	C	T	DICER1	NM_177438.2:c.3553G>A	NP_803187.1:p.Ala1185Thr	missense	LB	2.71E-05	.	1.76E-05	T	20.1	0.048	Conflicting_interpretations_of_pathogenicity (Last reviewed: Dec 31, 2019)	137709	Acute_myeloid_leukemia|Hereditary_cancer-predisposing_syndrome|DICER1-related_pleuropulmonary_blastoma_cancer_predisposition_syndrome|not_specified|not_provided	criteria_provided, conflicting_interpretations
14	95103850	T	C	DICER1	NM_177438.2:c.3546A>G	NP_803187.1:p.Gln1182=	synonymous	LB	1.63E-05	.	.	.	.	.	Uncertain_significance (Last reviewed: Jan 15, 2018)	573027	DICER1-related_pleuropulmonary_blastoma_cancer_predisposition_syndrome	criteria_provided, single_submitter
14	95103860	G	C	DICER1	NM_177438.2:c.3536C>G	NP_803187.1:p.Ser1179Cys	missense	pDeleterious	2.71E-05	.	.	D	23.5	0.18	Uncertain_significance (Last reviewed: Apr 23, 2019)	400035	Hereditary_cancer-predisposing_syndrome|DICER1-related_pleuropulmonary_blastoma_cancer_predisposition_syndrome	criteria_provided, multiple_submitters, no_conflicts
14	95103867	C	A	DICER1	NM_177438.2:c.3529G>T	NP_803187.1:p.Gly1177Cys	missense	pDeleterious	5.42E-06	.	0	D	24.3	0.542	Uncertain_significance (Last reviewed: Jun 12, 2018)	812033	Hereditary_cancer-predisposing_syndrome	criteria_provided, single_submitter
14	95103874	T	A	DICER1	NM_177438.2:c.3522A>T	NP_803187.1:p.Ala1174=	synonymous	LB	1.08E-05	.	.	.	.	.	Likely_benign (Last reviewed: Dec 31, 2019)	464327	DICER1-related_pleuropulmonary_blastoma_cancer_predisposition_syndrome	criteria_provided, single_submitter
14	95103877	T	C	DICER1	NM_177438.2:c.3519A>G	NP_803187.1:p.Thr1173=	synonymous	LB	0.0001571	.	3.52E-05	.	.	.	Likely_benign (Last reviewed: Dec 31, 2019)	222403	Hereditary_cancer-predisposing_syndrome|not_provided	criteria_provided, multiple_submitters, no_conflicts
14	95103900	C	T	DICER1	NM_177438.2:c.3496G>A	NP_803187.1:p.Val1166Ile	missense	LB	1.63E-05	.	0	T	8.916	0.035	Uncertain_significance (Last reviewed: Nov 13, 2019)	241950	DICER1-related_pleuropulmonary_blastoma_cancer_predisposition_syndrome	criteria_provided, single_submitter
14	95103901	G	A	DICER1	NM_177438.2:c.3495C>T	NP_803187.1:p.His1165=	synonymous	LB	4.33E-05	1.84E-05	3.52E-05	.	.	.	Likely_benign (Last reviewed: Dec 31, 2019)	400385	Hereditary_cancer-predisposing_syndrome|not_provided	criteria_provided, multiple_submitters, no_conflicts
14	95103917	G	T	DICER1	NM_177438.2:c.3479C>A	NP_803187.1:p.Ser1160Tyr	missense	pDeleterious	1.63E-05	.	0	D	22.1	0.163	Uncertain_significance (Last reviewed: Dec 11, 2019)	241951	Hereditary_cancer-predisposing_syndrome|DICER1-related_pleuropulmonary_blastoma_cancer_predisposition_syndrome	criteria_provided, multiple_submitters, no_conflicts
14	95103922	G	A	DICER1	NM_177438.2:c.3474C>T	NP_803187.1:p.Ser1158=	synonymous	LB	5.42E-06	3.68E-05	2.64E-05	.	.	.	Likely_benign (Last reviewed: Dec 31, 2019)	477116	Hereditary_cancer-predisposing_syndrome|not_provided	criteria_provided, multiple_submitters, no_conflicts
14	95103931	C	T	DICER1	NM_177438.2:c.3465G>A	NP_803187.1:p.Thr1155=	synonymous	LB	5.42E-06	.	0	.	.	.	Likely_benign (Last reviewed: Dec 31, 2019)	464666	Hereditary_cancer-predisposing_syndrome|not_provided	criteria_provided, multiple_submitters, no_conflicts

© 2021 Mirshahi UL et al. *JAMA Network Open.*

14	95103937	G	A	DICER1	NM_177438.2:c.3459C>T	NP_803187.1:p.Cys1153=	synonymous	LB	1.08E-05	.	.	.	.	.	Likely_benign (Last reviewed: Dec 31, 2019)	528923	Hereditary_cancer-predisposing_syndrome|DICER1-related_pleuropulmonary_blastoma_cancer_predisposition_syndrome	criteria_provided, multiple_submitters, no_conflicts

© 2021 Mirshahi UL et al. *JAMA Network Open.*

14	95103943	C	T	DICER1	NM_177438.2:c.3453G>A	NP_803187.1:p.Val1151=	synonymous	LB	5.42E-06	.	.	.	.	.	.	.	.	.
14	95103949	C	T	DICER1	NM_177438.2:c.3447G>A	NP_803187.1:p.Met1149Ile	missense	LB	5.42E-06	.	8.81E-06	T	17.8	0.097	Uncertain_significance (Last reviewed: Dec 16, 2019)	812041	Hereditary_cancer-predisposing_syndrome	criteria_provided, single_submitter
14	95103958	A	G	DICER1	NM_177438.2:c.3438T>C	NP_803187.1:p.His1146=	synonymous	LB	5.42E-06	.	.	.	.	.	Likely_benign (Last reviewed: Nov 6, 2018)	784831	not_provided	criteria_provided, single_submitter
14	95103968	A	G	DICER1	NM_177438.2:c.3428T>C	NP_803187.1:p.Leu1143Pro	missense	LB	4.33E-05	.	0	T	12.53	0.102	Conflicting_interpretations_of_pathogenicity (Last reviewed: Dec 31, 2019)	222405	Hereditary_cancer-predisposing_syndrome|not_provided	criteria_provided, conflicting_interpretations
14	95103974	G	A	DICER1	NM_177438.2:c.3422C>T	NP_803187.1:p.Ser1141Phe	missense	LB	5.42E-06	1.84E-05	2.65E-05	T	23.3	0.126	Uncertain_significance (Last reviewed: Dec 31, 2019)	400041	Hereditary_cancer-predisposing_syndrome|DICER1-related_pleuropulmonary_blastoma_cancer_predisposition_syndrome	criteria_provided, multiple_submitters, no_conflicts
14	95103977	GTTC	G	DICER1	NM_177438.2:c.3416_3419delinsC	NP_803187.1:p.Arg1139del	inframe_deletion	VUS	1.08E-05	.	.	.	.	.	.	.	.	.
14	95103991	T	C	DICER1	NM_177438.2:c.3405A>G	NP_803187.1:p.Gln1135=	synonymous	LB	5.42E-06	.	.	.	.	.	Likely_benign (Last reviewed: Apr 19, 2017)	477345	Hereditary_cancer-predisposing_syndrome	criteria_provided, single_submitter
14	95104004	T	C	DICER1	NM_177438.2:c.3392A>G	NP_803187.1:p.Asn1131Ser	missense	LB	4.33E-05	.	8.83E-06	T	0.339	0.058	Conflicting_interpretations_of_pathogenicity (Last reviewed: Dec 31, 2019)	400059	Hereditary_cancer-predisposing_syndrome|not_provided	criteria_provided, conflicting_interpretations
14	95104016	A	C	DICER1	NM_177438.2:c.3380T>G	NP_803187.1:p.Ile1127Ser	missense	LB	5.42E-06	.	0	T	10.9	0.117	Uncertain_significance (Last reviewed: Dec 23, 2019)	399845	Hereditary_cancer-predisposing_syndrome|DICER1-related_pleuropulmonary_blastoma_cancer_predisposition_syndrome	criteria_provided, multiple_submitters, no_conflicts
14	95104036	A	G	DICER1	NM_177438.2:c.3360T>C	NP_803187.1:p.Asn1120=	synonymous	LB	5.42E-06	.	.	.	.	.	Likely_benign (Last reviewed: Dec 31, 2019)	693629	Hereditary_cancer-predisposing_syndrome|not_provided	criteria_provided, multiple_submitters, no_conflicts
14	95104043	T	C	DICER1	NM_177438.2:c.3353A>G	NP_803187.1:p.Asn1118Ser	missense	LB	5.42E-06	.	1.76E-05	T	0.002	0.037	Uncertain_significance (Last reviewed: Dec 29, 2018)	399849	DICER1-related_pleuropulmonary_blastoma_cancer_predisposition_syndrome	criteria_provided, single_submitter
14	95104048	A	T	DICER1	NM_177438.2:c.3348T>A	NP_803187.1:p.Ala1116=	synonymous	LB	5.42E-06	.	8.82E-06	.	.	.	Uncertain_significance (Last reviewed: Oct 14, 2018)	642866	DICER1-related_pleuropulmonary_blastoma_cancer_predisposition_syndrome	criteria_provided, single_submitter
14	95104058	G	T	DICER1	NM_177438.2:c.3338C>A	NP_803187.1:p.Ser1113Tyr	missense	LB	5.42E-06	.	0	T	11.13	0.088	Uncertain_significance (Last reviewed: Jan 6, 2020)	400060	Hereditary_cancer-predisposing_syndrome|DICER1-related_pleuropulmonary_blastoma_cancer_predisposition_syndrome	criteria_provided, multiple_submitters, no_conflicts
14	95104062	T	C	DICER1	NM_177438.2:c.3334A>G	NP_803187.1:p.Asn1112Asp	missense	LB	2.17E-05	.	0	T	0.06	0.109	Conflicting_interpretations_of_pathogenicity (Last reviewed: Dec 31, 2019)	137708	Pleuropulmonary_blastoma|Hereditary_cancer-predisposing_syndrome|not_specified|not_provided	criteria_provided, conflicting_interpretations
14	95104067	A	C	DICER1	NM_177438.2:c.3329T>G	NP_803187.1:p.Ile1110Ser	missense	LB	1.08E-05	.	8.82E-06	T	0.001	0.066	Uncertain_significance (Last reviewed: Dec 30, 2019)	400663	Hereditary_cancer-predisposing_syndrome|DICER1-related_pleuropulmonary_blastoma_cancer_predisposition_syndrome	criteria_provided, multiple_submitters, no_conflicts
14	95104090	A	T	DICER1	NM_177438.2:c.3306T>A	NP_803187.1:p.Ile1102=	synonymous	LB	1.08E-05	.	.	.	.	.	Likely_benign (Last reviewed: Dec 31, 2019)	784832	not_provided	criteria_provided, single_submitter
14	95104093	A	G	DICER1	NM_177438.2:c.3303T>C	NP_803187.1:p.Ser1101=	synonymous	LB	1.08E-05	.	0	.	.	.	Likely_benign (Last reviewed: Dec 7, 2017)	528392	DICER1-related_pleuropulmonary_blastoma_cancer_predisposition_syndrome	criteria_provided, single_submitter
14	95104100	T	C	DICER1	NM_177438.2:c.3296A>G	NP_803187.1:p.Lys1099Arg	missense	LB	7.58E-05	.	.	T	15.71	0.169	Uncertain_significance (Last reviewed: May 25, 2018)	566726	Hereditary_cancer-predisposing_syndrome|DICER1-related_pleuropulmonary_blastoma_cancer_predisposition_syndrome	criteria_provided, multiple_submitters, no_conflicts
14	95104108	G	A	DICER1	NM_177438.2:c.3288C>T	NP_803187.1:p.Phe1096=	synonymous	LB	1.08E-05	.	1.77E-05	.	.	.	Likely_benign (Last reviewed: Dec 31, 2019)	464341	Hereditary_cancer-predisposing_syndrome|not_provided	criteria_provided, multiple_submitters, no_conflicts
14	95104111	G	A	DICER1	NM_177438.2:c.3285C>T	NP_803187.1:p.Asp1095=	synonymous	LB	1.08E-05	1.94E-05	2.65E-05	.	.	.	Likely_benign (Last reviewed: Dec 31, 2019)	400666	not_provided	criteria_provided, single_submitter
14	95104120	A	T	DICER1	NM_177438.2:c.3276T>A	NP_803187.1:p.Pro1092=	synonymous	LB	1.08E-05	.	.	.	.	.	Likely_benign (Last reviewed: Dec 31, 2019)	477122	Hereditary_cancer-predisposing_syndrome|not_provided	criteria_provided, multiple_submitters, no_conflicts
14	95104123	G	A	DICER1	NM_177438.2:c.3273C>T	NP_803187.1:p.Tyr1091=	synonymous	LB	1.63E-05	.	.	.	.	.	Likely_benign (Last reviewed: Dec 31, 2019)	463719	not_provided	criteria_provided, single_submitter
14	95105079	C	T	DICER1	NM_177438.2:c.3261G>A	NP_803187.1:p.Ala1087=	synonymous	LB	0.0005851	0.0003	0.0003	T	13.33	0.178	Likely_benign (Last reviewed: Dec 31, 2019)	241954	Hereditary_cancer-predisposing_syndrome|not_specified|not_provided	criteria_provided, multiple_submitters, no_conflicts
14	95105080	G	A	DICER1	NM_177438.2:c.3260C>T	NP_803187.1:p.Ala1087Val	missense	LB	3.25E-05	1.84E-05	3.52E-05	T	16.41	0.158	Uncertain_significance (Last reviewed: Dec 26, 2019)	400675	Hereditary_cancer-predisposing_syndrome|DICER1-related_pleuropulmonary_blastoma_cancer_predisposition_syndrome|not_provided	criteria_provided, multiple_submitters, no_conflicts
14	95105106	A	G	DICER1	NM_177438.2:c.3234T>C	NP_803187.1:p.Ala1078=	synonymous	LB	5.42E-06	1.84E-05	1.76E-05	T	8.301	0.184	Likely_benign (Last reviewed: Dec 31, 2019)	463721	Hereditary_cancer-predisposing_syndrome|not_provided	criteria_provided, multiple_submitters, no_conflicts
14	95105127	T	C	DICER1	NM_177438.2:c.3213A>G	NP_803187.1:p.Arg1071=	synonymous	LB	0.0001083	3.68E-05	8.80E-06	.	.	.	Benign/Likely_benign (Last reviewed: Dec 31, 2019)	241955	Hereditary_cancer-predisposing_syndrome|not_provided	criteria_provided, multiple_submitters, no_conflicts
14	95105143	G	A	DICER1	NM_177438.2:c.3197C>T	NP_803187.1:p.Thr1066Ile	missense	LB	5.42E-06	.	.	T	24.1	0.335	Uncertain_significance (Last reviewed: Nov 18, 2019)	812062	Hereditary_cancer-predisposing_syndrome	criteria_provided, single_submitter
14	95105154	G	A	DICER1	NM_177438.2:c.3186C>T	NP_803187.1:p.His1062=	synonymous	LB	1.63E-05	.	.	.	.	.	Likely_benign (Last reviewed: Dec 26, 2017)	528456	DICER1-related_pleuropulmonary_blastoma_cancer_predisposition_syndrome	criteria_provided, single_submitter
14	95105160	G	A	DICER1	NM_177438.2:c.3180C>T	NP_803187.1:p.Arg1060=	synonymous	LB	5.42E-06	.	.	.	.	.	.	.	.	.
14	95105166	A	C	DICER1	NM_177438.2:c.3174T>G	NP_803187.1:p.Leu1058=	synonymous	LB	5.42E-06	.	.	.	.	.	.	.	.	.
14	95105178	G	A	DICER1	NM_177438.2:c.3162C>T	NP_803187.1:p.Leu1054=	synonymous	LB	3.25E-05	.	.	.	.	.	Likely_benign (Last reviewed: Dec 31, 2019)	399869	Hereditary_cancer-predisposing_syndrome|not_provided	criteria_provided, multiple_submitters, no_conflicts
14	95105190	T	C	DICER1	NM_177438.2:c.3150A>G	NP_803187.1:p.Lys1050=	synonymous	LB	5.42E-06	.	.	.	.	.	Likely_benign (Last reviewed: Sep 10, 2018)	812069	Hereditary_cancer-predisposing_syndrome	criteria_provided, single_submitter
14	95105205	T	C	DICER1	NM_177438.2:c.3135A>G	NP_803187.1:p.Ala1045=	synonymous	LB	1.08E-05	.	.	.	.	.	.	.	.	.

© 2021 Mirshahi UL et al. *JAMA Network Open.*

<table>
<tr><td>14</td><td>95105213</td><td>T</td><td>C</td><td>DICER1</td><td>NM_177438.2:c.3127A>G</td><td>NP_803187.1:p.Ile1043Val</td><td>missense</td><td>LB</td><td>1.08E-05</td><td>.</td><td>1.76E-05</td><td>T</td><td>12.05</td><td>0.097</td><td>Uncertain_significance (Last reviewed: Dec 11, 2019)</td><td>399871</td><td>Hereditary_cancer-predisposing_syndrome|DICER1-related_pleuropulmonary_blastoma_cancer_predisposition_syndrome</td><td>criteria_provided, multiple_submitters, no_conflicts</td></tr>
</table>

© 2021 Mirshahi UL et al. *JAMA Network Open.*

14	95105241	C	A	DICER1	NM_177438.2:c.3099G>T	NP_803187.1:p.Leu1033=	synonymous	LB	1.08E-05	7.36E-05	3.52E-05	.	.	.	Likely_benign (Last reviewed: Dec 31, 2019)	400685	not_provided	criteria_provided, single_submitter
14	95105697	T	C	DICER1	NM_177438.2:c.3074A>G	NP_803187.1:p.Glu1025Gly	missense	LB	5.42E-06	.	8.80E-06	T	26.8	0.533	Uncertain_significance (Last reviewed: Nov 20, 2019)	463727	Hereditary_cancer-predisposing_syndrome|DICER1-related_pleuropulmonary_blastoma_cancer_predisposition_syndrome	criteria_provided, multiple_submitters, no_conflicts
14	95105739	G	A	DICER1	NM_177438.2:c.3032C>T	NP_803187.1:p.Ala1011Val	missense	LB	1.08E-05	.	3.52E-05	T	28.9	0.254	Uncertain_significance (Last reviewed: Dec 5, 2019)	528932	Hereditary_cancer-predisposing_syndrome|DICER1-related_pleuropulmonary_blastoma_cancer_predisposition_syndrome	criteria_provided, multiple_submitters, no_conflicts
14	95105768	T	C	DICER1	NM_177438.2:c.3003A>G	NP_803187.1:p.Thr1001=	synonymous	LB	3.25E-05	.	.	.	.	.	Likely_benign (Last reviewed: Dec 31, 2019)	784834	not_provided	criteria_provided, single_submitter
14	95106077	T	C	DICER1	NM_177438.2:c.2951A>G	NP_803187.1:p.Asn984Ser	missense	LB	1.08E-05	1.87E-05	8.82E-06	T	8.572	0.136	Uncertain_significance (Last reviewed: Dec 11, 2019)	400713	Hereditary_cancer-predisposing_syndrome|DICER1-related_pleuropulmonary_blastoma_cancer_predisposition_syndrome	criteria_provided, multiple_submitters, no_conflicts
14	95106081	G	T	DICER1	NM_177438.2:c.2947C>A	NP_803187.1:p.Leu983Ile	missense	LB	5.42E-06	.	.	T	22.8	0.078	.	.	.	.
14	95106124	A	G	DICER1	NM_177438.2:c.2904T>C	NP_803187.1:p.Phe968=	synonymous	LB	5.42E-06	.	2.64E-05	.	.	.	Likely_benign (Last reviewed: Dec 31, 2019)	399881	Hereditary_cancer-predisposing_syndrome|not_provided	criteria_provided, multiple_submitters, no_conflicts
14	95106130	T	C	DICER1	NM_177438.2:c.2898A>G	NP_803187.1:p.Glu966=	synonymous	LB	5.42E-06	.	0	.	.	.	Likely_benign (Last reviewed: Dec 31, 2019)	241960	Hereditary_cancer-predisposing_syndrome|not_provided	criteria_provided, multiple_submitters, no_conflicts
14	95106136	C	T	DICER1	NM_177438.2:c.2892G>A	NP_803187.1:p.Glu964=	synonymous	LB	5.42E-06	.	.	.	.	.	.	.	.	.
14	95106156	T	C	DICER1	NM_177438.2:c.2872A>G	NP_803187.1:p.Ser958Gly	missense	LB	1.08E-05	.	2.64E-05	T	26.8	0.528	Uncertain_significance (Last reviewed: Dec 24, 2019)	241961	Hereditary_cancer-predisposing_syndrome|DICER1-related_pleuropulmonary_blastoma_cancer_predisposition_syndrome	criteria_provided, multiple_submitters, no_conflicts
14	95106223	T	C	DICER1	NM_177438.2:c.2805A>G	NP_803187.1:p.Arg935=	synonymous	LB	2.17E-05	.	.	.	.	.	Likely_benign (Last reviewed: Feb 6, 2019)	812081	Hereditary_cancer-predisposing_syndrome	criteria_provided, single_submitter
14	95106223	T	TA	DICER1	NM_177438.2:c.2805-2delinsTA		splice_acceptor	pLOF	0.0001433	.	.	.	.	.	.	.	.	.
14	95107613	A	C	DICER1	NM_177438.2:c.2799T>G	NP_803187.1:p.Ile933Met	missense	LB	5.42E-06	.	.	T	15.63	0.2	.	.	.	.
14	95107631	G	A	DICER1	NM_177438.2:c.2781C>T	NP_803187.1:p.Tyr927=	synonymous	LB	1.08E-05	.	.	.	.	.	Likely_benign (Last reviewed: Dec 31, 2019)	400388	Hereditary_cancer-predisposing_syndrome|DICER1-related_pleuropulmonary_blastoma_cancer_predisposition_syndrome	criteria_provided, multiple_submitters, no_conflicts
14	95107650	A	G	DICER1	NM_177438.2:c.2762T>C	NP_803187.1:p.Val921Ala	missense	LB	5.42E-06	.	.	T	11.77	0.055	.	.	.	.
14	95107652	A	G	DICER1	NM_177438.2:c.2760T>C	NP_803187.1:p.Phe920=	synonymous	LB	1.08E-05	.	.	.	.	.	.	.	.	.
14	95107657	G	T	DICER1	NM_177438.2:c.2755C>A	NP_803187.1:p.Pro919Thr	missense	LB	1.08E-05	.	.	T	23.4	0.407	.	.	.	.
14	95107675	T	C	DICER1	NM_177438.2:c.2737A>G	NP_803187.1:p.Lys913Glu	missense	LB	5.42E-06	.	.	T	10.04	0.057	Uncertain_significance (Last reviewed: Jul 8, 2018)	642877	DICER1-related_pleuropulmonary_blastoma_cancer_predisposition_syndrome	criteria_provided, single_submitter
14	95107692	A	G	DICER1	NM_177438.2:c.2720T>C	NP_803187.1:p.Ile907Thr	missense	LB	7.58E-05	5.53E-05	6.16E-05	T	10.9	0.073	Conflicting interpretations of _pathogenicity (Last reviewed: Dec 20, 2019)	400092	Acute_megakaryoblastic_leukemia|Hereditary_canc er-predisposing_syndrome|not_provided	criteria_provided, conflicting_interpretations
14	95107693	T	C	DICER1	NM_177438.2:c.2719A>G	NP_803187.1:p.Ile907Val	missense	LB	5.42E-06	.	0	T	9.399	0.062	.	.	.	.
14	95107694	G	A	DICER1	NM_177438.2:c.2718C>T	NP_803187.1:p.Arg906=	synonymous	LB	5.42E-06	0.0002	7.04E-05	.	.	.	Likely_benign (Last reviewed: Dec 31, 2019)	463737	Hereditary_cancer-predisposing_syndrome|not_provided	criteria_provided, multiple_submitters, no_conflicts
14	95107695	C	T	DICER1	NM_177438.2:c.2717G>A	NP_803187.1:p.Arg906His	missense	LB	1.08E-05	.	8.80E-06	T	28.3	0.312	Uncertain_significance (Last reviewed: Dec 27, 2019)	241964	Hereditary_cancer-predisposing_syndrome|DICER1-related_pleuropulmonary_blastoma_cancer_predisposition_syndrome	criteria_provided, multiple_submitters, no_conflicts
14	95107723	T	C	DICER1	NM_177438.2:c.2689A>G	NP_803187.1:p.Met897Val	missense	LB	1.08E-05	.	8.80E-06	T	9.652	0.154	Uncertain_significance (Last reviewed: May 2, 2019)	400094	Hereditary_cancer-predisposing_syndrome|DICER1-related_pleuropulmonary_blastoma_cancer_predisposition_syndrome	criteria_provided, multiple_submitters, no_conflicts
14	95107731	A	G	DICER1	NM_177438.2:c.2681T>C	NP_803187.1:p.Phe894Ser	missense	LB	5.42E-06	.	.	T	29.3	0.518	.	.	.	.
14	95107738	T	C	DICER1	NM_177438.2:c.2674A>G	NP_803187.1:p.Ile892Val	missense	LB	5.42E-06	.	.	T	8.853	0.097	Uncertain_significance (Last reviewed: Aug 13, 2019)	812093	Hereditary_cancer-predisposing_syndrome	criteria_provided, single_submitter
14	95107753	A	G	DICER1	NM_177438.2:c.2659T>C	NP_803187.1:p.Ser887Pro	missense	LB	5.42E-06	.	.	T	24.9	0.237	Uncertain_significance (Last reviewed: Nov 19, 2019)	241965	Hereditary_cancer-predisposing_syndrome|DICER1-related_pleuropulmonary_blastoma_cancer_predisposition_syndrome	criteria_provided, multiple_submitters, no_conflicts
14	95107754	G	A	DICER1	NM_177438.2:c.2658C>T	NP_803187.1:p.Asp886=	synonymous	LB	9.75E-05	7.42E-05	0.0001	.	.	.	Likely_benign (Last reviewed: Dec 31, 2019)	399893	Hereditary_cancer-predisposing_syndrome|not_provided	criteria_provided, multiple_submitters, no_conflicts
14	95107758	T	G	DICER1	NM_177438.2:c.2654A>C	NP_803187.1:p.Asn885Thr	missense	LB	1.63E-05	.	.	T	14.03	0.03	.	.	.	.
14	95107882	A	G	DICER1	NM_177438.2:c.2648T>C	NP_803187.1:p.Val883Ala	missense	LB	1.08E-05	.	8.82E-06	T	13.07	0.192	.	.	.	.
14	95107883	C	T	DICER1	NM_177438.2:c.2647G>A	NP_803187.1:p.Val883Ile	missense	LB	3.25E-05	.	.	T	17.84	0.042	Uncertain_significance (Last reviewed: Mar 15, 2019)	528475	Hereditary_cancer-predisposing_syndrome|DICER1-related_pleuropulmonary_blastoma_cancer_predisposition_syndrome	criteria_provided, multiple_submitters, no_conflicts
14	95107889	G	C	DICER1	NM_177438.2:c.2641C>G	NP_803187.1:p.Leu881Val	missense	pDeleterious	4.33E-05	.	.	D	24.1	0.493	Uncertain_significance (Last reviewed: Dec 23, 2019)	642880	Hereditary_cancer-predisposing_syndrome|DICER1-related_pleuropulmonary_blastoma_cancer_predisposition_syndrome	criteria_provided, multiple_submitters, no_conflicts
14	95107907	C	A	DICER1	NM_177438.2:c.2623G>T	NP_803187.1:p.Ala875Ser	missense	LB	5.42E-06	.	.	T	23.7	0.488	Uncertain_significance (Last reviewed: May 22, 2017)	463758	DICER1-related_pleuropulmonary_blastoma_cancer_predisposition_syndrome	criteria_provided, single_submitter
14	95107908	T	C	DICER1	NM_177438.2:c.2622A>G	NP_803187.1:p.Ser874=	synonymous	LB	5.42E-06	.	.	.	.	.	.	.	.	.
14	95107917	G	A	DICER1	NM_177438.2:c.2613C>T	NP_803187.1:p.Asp871=	synonymous	LB	1.63E-05	5.53E-05	5.29E-05	.	.	.	Likely_benign (Last reviewed: Dec 31, 2019)	400400	Hereditary_cancer-predisposing_syndrome|not_provided	criteria_provided, multiple_submitters, no_conflicts
14	95107950	A	C	DICER1	NM_177438.2:c.2580T>G	NP_803187.1:p.Leu860=	synonymous	LB	1.08E-05	.	.	.	.	.	.	.	.	.
14	95107955	G	A	DICER1	NM_177438.2:c.2575C>T	NP_803187.1:p.Arg859Trp	missense	pDeleterious	5.42E-06	.	0	D	35	0.766	Uncertain_significance (Last reviewed: Sep 20, 2019)	241966	Hereditary_cancer-predisposing_syndrome|DICER1-related_pleuropulmonary_blastoma_cancer_predisposition_syndrome	criteria_provided, multiple_submitters, no_conflicts

© 2021 Mirshahi UL et al. *JAMA Network Open.*

14	95107958	G	A	DICER1	NM_177438.2:c.2572C>T	NP_803187.1:p.Leu858Phe	missense	pDeleterious	5.42E-06	.	.	D	29	0.664	.	.	.	.	

© 2021 Mirshahi UL et al. *JAMA Network Open.*

14	95107965	T	C	DICER1	NM_177438.2:c.2565A>G	NP_803187.1:p.Ser855=	synonymous	LB	2.17E-05	5.52E-05	4.40E-05	.	.	.	Likely_benign (Last reviewed: Dec 31, 2019)	241967	Hereditary_cancer-predisposing_syndrome|not_provided	criteria_provided, multiple_submitters, no_conflicts
14	95107973	T	C	DICER1	NM_177438.2:c.2557A>G	NP_803187.1:p.Ile853Val	missense	LB	0.00013	.	0	T	6.68	0.286	Benign/Likely_benign (Last reviewed: Dec 31, 2019)	137705	Hereditary_cancer-predisposing_syndrome|not_specified|not_provided	criteria_provided, multiple_submitters, no_conflicts
14	95107977	C	T	DICER1	NM_177438.2:c.2553G>A	NP_803187.1:p.Gln851=	synonymous	LB	9.21E-05	9.21E-05	0.0001	.	.	.	Benign/Likely_benign (Last reviewed: Dec 31, 2019)	399905	Hereditary_cancer-predisposing_syndrome|not_provided	criteria_provided, multiple_submitters, no_conflicts
14	95107990	G	A	DICER1	NM_177438.2:c.2540C>T	NP_803187.1:p.Thr847Ile	missense	LB	5.42E-06	.	.	T	25	0.583	Uncertain_significance (Last reviewed: Jul 6, 2018)	812103	Hereditary_cancer-predisposing_syndrome	criteria_provided, single_submitter
14	95107994	T	C	DICER1	NM_177438.2:c.2536A>G	NP_803187.1:p.Ile846Val	missense	LB	7.04E-05	9.21E-05	6.17E-05	T	11.01	0.191	Uncertain_significance (Last reviewed: Dec 27, 2019)	464635	Goiter,_multinodular_1,_with_or_without_Sertoli-Leydig_cell_tumors|Hereditary_cancer-predisposing_syndrome|Rhabdomyosarcoma,_embryonal,_2|DICER1-related_pleuropulmonary_blastoma_cancer_predisposition_syndrome	criteria_provided, multiple_submitters, no_conflicts
14	95108019	C	T	DICER1	NM_177438.2:c.2511G>A	NP_803187.1:p.Met837Ile	missense	LB	5.42E-06	.	.	T	3.129	0.106	.	.	.	.
14	95108021	T	C	DICER1	NM_177438.2:c.2509A>G	NP_803187.1:p.Met837Val	missense	LB	1.08E-05	.	.	T	7.166	0.072	.	.	.	.
14	95108024	A	C	DICER1	NM_177438.2:c.2506T>G	NP_803187.1:p.Phe836Val	missense	LB	1.63E-05	.	.	T	12.16	0.419	Uncertain_significance (Last reviewed: Dec 30, 2019)	573048	Hereditary_cancer-predisposing_syndrome|DICER1-related_pleuropulmonary_blastoma_cancer_predisposition_syndrome	criteria_provided, multiple_submitters, no_conflicts
14	95108030	A	G	DICER1	NM_177438.2:c.2500T>C	NP_803187.1:p.Ser834Pro	missense	pDeleterious	5.42E-06	.	.	D	28	0.713	.	.	.	.
14	95108034	C	G	DICER1	NM_177438.2:c.2496G>C	NP_803187.1:p.Lys832Asn	missense	LB	1.08E-05	3.68E-05	1.76E-05	T	21.9	0.406	Uncertain_significance (Last reviewed: Apr 26, 2019)	339978	Pleuropulmonary_blastoma|Hereditary_cancer-predisposing_syndrome	criteria_provided, multiple_submitters, no_conflicts
14	95108036	T	C	DICER1	NM_177438.2:c.2494A>G	NP_803187.1:p.Lys832Glu	missense	LB	3.79E-05	.	.	T	10.23	0.344	Uncertain_significance (Last reviewed: Dec 22, 2019)	399906	Goiter,_multinodular_1,_with_or_without_Sertoli-Leydig_cell_tumors|Hereditary_cancer-predisposing_syndrome|Rhabdomyosarcoma,_embryonal,_2|DICER1-related_pleuropulmonary_blastoma_cancer_predisposition_syndrome	criteria_provided, multiple_submitters, no_conflicts
14	95108057	C	G	DICER1	NM_177438.2:c.2473G>C	NP_803187.1:p.Val825Leu	missense	LB	5.42E-06	.	.	T	25.2	0.484	.	.	.	.
14	95108067	G	T	DICER1	NM_177438.2:c.2463C>A	NP_803187.1:p.Arg821=	synonymous	LB	1.63E-05	.	.	.	.	.	Likely_benign (Last reviewed: Dec 31, 2019)	754239	Hereditary_cancer-predisposing_syndrome|not_provided	criteria_provided, multiple_submitters, no_conflicts
14	95108075	A	G	DICER1	NM_177438.2:c.2455T>C	NP_803187.1:p.Tyr819His	missense	pDeleterious	5.42E-06	.	8.83E-06	D	27.8	0.876	Uncertain_significance (Last reviewed: Dec 19, 2019)	477726	Hereditary_cancer-predisposing_syndrome	criteria_provided, single_submitter
14	95108091	A	G	DICER1	NM_177438.2:c.2439T>C	NP_803187.1:p.Ile813=	synonymous	LB	1.63E-05	.	.	.	.	.	.	.	.	.
14	95108342	C	T	DICER1	NM_177438.2:c.2418G>A	NP_803187.1:p.Thr806=	synonymous	LB	5.42E-06	.	0	.	.	.	.	.	.	.
14	95108343	G	A	DICER1	NM_177438.2:c.2417C>T	NP_803187.1:p.Thr806Met	missense	pDeleterious	2.17E-05	.	8.80E-06	D	33	0.746	Uncertain_significance (Last reviewed: Dec 19, 2019)	399916	DICER1-related_pleuropulmonary_blastoma_cancer_predisposition_syndrome	criteria_provided, single_submitter
14	95108367	G	A	DICER1	NM_177438.2:c.2393C>T	NP_803187.1:p.Thr798Ile	missense	pDeleterious	5.42E-06	.	.	D	29.7	0.534	.	.	.	.
14	95108382	T	C	DICER1	NM_177438.2:c.2378A>G	NP_803187.1:p.Tyr793Cys	missense	pDeleterious	3.79E-05	1.84E-05	8.80E-06	D	24	0.762	Uncertain_significance (Last reviewed: Nov 18, 2019)	400404	Hereditary_cancer-predisposing_syndrome|DICER1-related_pleuropulmonary_blastoma_cancer_predisposition_syndrome	criteria_provided, multiple_submitters, no_conflicts
14	95108411	A	G	DICER1	NM_177438.2:c.2349T>C	NP_803187.1:p.Asp783=	synonymous	LB	1.63E-05	3.68E-05	2.64E-05	.	.	.	Likely_benign (Last reviewed: Dec 31, 2019)	464687	Hereditary_cancer-predisposing_syndrome|not_provided	criteria_provided, multiple_submitters, no_conflicts
14	95108423	T	C	DICER1	NM_177438.2:c.2337A>G	NP_803187.1:p.Thr779=	synonymous	LB	7.04E-05	7.36E-05	8.80E-05	.	.	.	Likely_benign (Last reviewed: Dec 31, 2019)	400102	Hereditary_cancer-predisposing_syndrome|not_specified|not_provided	criteria_provided, multiple_submitters, no_conflicts
14	95108433	A	C	DICER1	NM_177438.2:c.2327T>G	NP_803187.1:p.Val776Gly	missense	LB	5.42E-06	.	.	T	22.9	0.368	Uncertain_significance (Last reviewed: Mar 1, 2019)	400413	Hereditary_cancer-predisposing_syndrome|DICER1-related_pleuropulmonary_blastoma_cancer_predisposition_syndrome	criteria_provided, multiple_submitters, no_conflicts
14	95108444	C	T	DICER1	NM_177438.2:c.2316G>A	NP_803187.1:p.Val772=	synonymous	LB	1.08E-05	.	.	.	.	.	Likely_benign (Last reviewed: Dec 31, 2019)	241969	Hereditary_cancer-predisposing_syndrome|not_provided	criteria_provided, multiple_submitters, no_conflicts
14	95108448	T	A	DICER1	NM_177438.2:c.2312A>T	NP_803187.1:p.Tyr771Phe	missense	pDeleterious	5.42E-06	.	.	D	29	0.666	.	.	.	.
14	95108482	T	C	DICER1	NM_177438.2:c.2278A>G	NP_803187.1:p.Ser760Gly	missense	LB	5.42E-06	.	.	T	23.6	0.138	.	.	.	.
14	95111332	C	T	DICER1	NM_177438.2:c.2241G>A	NP_803187.1:p.Gln747=	synonymous	LB	5.42E-06	.	0	.	.	.	Likely_benign (Last reviewed: Dec 31, 2019)	770005	not_provided	criteria_provided, single_submitter
14	95111344	C	T	DICER1	NM_177438.2:c.2229G>A	NP_803187.1:p.Thr743=	synonymous	LB	5.96E-05	.	0	.	.	.	Likely_benign (Last reviewed: Dec 31, 2019)	400722	Hereditary_cancer-predisposing_syndrome|not_provided	criteria_provided, multiple_submitters, no_conflicts
14	95111374	C	T	DICER1	NM_177438.2:c.2199G>A	NP_803187.1:p.Glu733=	synonymous	LB	1.08E-05	.	0	.	.	.	Likely_benign (Last reviewed: Dec 31, 2019)	464392	Hereditary_cancer-predisposing_syndrome|not_provided	criteria_provided, multiple_submitters, no_conflicts
14	95111382	C	T	DICER1	NM_177438.2:c.2191G>A	NP_803187.1:p.Glu731Lys	missense	LB	1.08E-05	.	.	T	25.6	0.141	Conflicting_interpretations_of_pathogenicity (Last reviewed: Aug 21, 2019)	223646	Hereditary_cancer-predisposing_syndrome|DICER1-related_pleuropulmonary_blastoma_cancer_predisposition_syndrome|Anophthalmia_-_microphthalmia	criteria_provided, conflicting_interpretations
14	95111440	A	G	DICER1	NM_177438.2:c.2133T>C	NP_803187.1:p.His711=	synonymous	LB	4.88E-05	.	8.80E-06	.	.	.	Likely_benign (Last reviewed: Dec 31, 2019)	241971	Hereditary_cancer-predisposing_syndrome|not_provided	criteria_provided, multiple_submitters, no_conflicts
14	95111451	G	A	DICER1	NM_177438.2:c.2122C>T	NP_803187.1:p.Leu708=	synonymous	LB	5.42E-06	.	.	.	.	.	.	.	.	.
14	95111455	G	A	DICER1	NM_177438.2:c.2118C>T	NP_803187.1:p.Gly706=	synonymous	LB	2.17E-05	.	3.52E-05	.	.	.	Conflicting_interpretations_of_pathogenicity (Last reviewed: Dec 22, 2019)	241972	Hereditary_cancer-predisposing_syndrome|DICER1-related_pleuropulmonary_blastoma_cancer_predisposition_syndrome	criteria_provided, conflicting_interpretations
14	95112173	A	G	DICER1	NM_177438.2:c.2115T>C	NP_803187.1:p.Ile705=	synonymous	LB	9.21E-05	.	0	.	.	.	Benign/Likely_benign (Last reviewed: Dec 31, 2019)	241974	Hereditary_cancer-predisposing_syndrome|not_specified|not_provided	criteria_provided, multiple_submitters, no_conflicts
14	95112177	T	C	DICER1	NM_177438.2:c.2111A>G	NP_803187.1:p.Lys704Arg	missense	LB	5.42E-06	3.68E-05	2.64E-05	T	23.7	0.089	Uncertain_significance (Last reviewed: Dec 31, 2019)	477730	Hereditary_cancer-predisposing_syndrome|DICER1-related_pleuropulmonary_blastoma_cancer_predisposition_syndrome	criteria_provided, multiple_submitters, no_conflicts

© 2021 Mirshahi UL et al. *JAMA Network Open.*

14	95112202	G	C	DICER1	NM_177438.2:c.2086C>G	NP_803187.1:p.Leu696Val	missense	LB	5.42E-06	.	.	T	24.4	0.428	Uncertain_significance (Last reviewed: Dec 23, 2018)	642897	DICER1-related_pleuropulmonary_blastoma_cancer_predisposition_syndrome	criteria_provided, single_submitter
14	95112212	T	C	DICER1	NM_177438.2:c.2076A>G	NP_803187.1:p.Arg692=	synonymous	LB	2.17E-05	.	8.80E-06	.	.	.	Likely_benign (Last reviewed: May 22, 2019)	739426	Hereditary_cancer-predisposing_syndrome|not_provided	criteria_provided, multiple_submitters, no_conflicts
14	95112217	C	G	DICER1	NM_177438.2:c.2071G>C	NP_803187.1:p.Glu691Gln	missense	LB	5.42E-06	.	.	T	21	0.172	.	.	.	.
14	95112225	C	T	DICER1	NM_177438.2:c.2063G>A	NP_803187.1:p.Arg688Gln	missense	LB	2.17E-05	1.84E-05	1.76E-05	T	25.1	0.227	Uncertain_significance (Last reviewed: Dec 11, 2019)	400726	Hereditary_cancer-predisposing_syndrome|DICER1-related_pleuropulmonary_blastoma_cancer_predisposition_syndrome	criteria_provided, multiple_submitters, no_conflicts
14	95112231	C	T	DICER1	NM_177438.2:c.2057G>A	NP_803187.1:p.Cys686Tyr	missense	LB	2.17E-05	1.84E-05	8.80E-06	T	25.7	0.211	Uncertain_significance (Last reviewed: Nov 20, 2019)	642898	Hereditary_cancer-predisposing_syndrome|DICER1-related_pleuropulmonary_blastoma_cancer_predisposition_syndrome	criteria_provided, multiple_submitters, no_conflicts
14	95112239	T	G	DICER1	NM_177438.2:c.2049A>C	NP_803187.1:p.Pro683=	synonymous	LB	5.42E-06	1.84E-05	8.80E-06	.	.	.	Likely_benign (Last reviewed: May 22, 2018)	770008	not_provided	criteria_provided, single_submitter
14	95113094	C	T	DICER1	NM_177438.2:c.2038G>A	NP_803187.1:p.Val680Ile	missense	LB	2.71E-05	.	1.76E-05	T	21.6	0.089	Uncertain_significance (Last reviewed: Dec 20, 2019)	400103	Hereditary_cancer-predisposing_syndrome|DICER1-related_pleuropulmonary_blastoma_cancer_predisposition_syndrome|not_provided	criteria_provided, multiple_submitters, no_conflicts
14	95113097	T	C	DICER1	NM_177438.2:c.2035A>G	NP_803187.1:p.Ile679Val	missense	LB	1.08E-05	.	8.80E-06	T	11.83	0.092	Uncertain_significance (Last reviewed: Feb 23, 2019)	642899	Hereditary_cancer-predisposing_syndrome|DICER1-related_pleuropulmonary_blastoma_cancer_predisposition_syndrome	criteria_provided, multiple_submitters, no_conflicts
14	95113103	C	G	DICER1	NM_177438.2:c.2029G>C	NP_803187.1:p.Ala677Pro	missense	LB	1.08E-05	.	.	T	25.2	0.155	.	.	.	.
14	95113105	C	G	DICER1	NM_177438.2:c.2027G>C	NP_803187.1:p.Arg676Pro	missense	LB	5.42E-06	.	.	T	34	0.471	Uncertain_significance (Last reviewed: Dec 5, 2019)	464698	Hereditary_cancer-predisposing_syndrome|DICER1-related_pleuropulmonary_blastoma_cancer_predisposition_syndrome	criteria_provided, multiple_submitters, no_conflicts
14	95113106	G	C	DICER1	NM_177438.2:c.2026C>G	NP_803187.1:p.Arg676Gly	missense	LB	5.42E-06	.	.	T	28.9	0.526	.	.	.	.
14	95113109	G	C	DICER1	NM_177438.2:c.2023C>G	NP_803187.1:p.Leu675Val	missense	LB	5.42E-06	.	.	T	24.2	0.272	Uncertain_significance (Last reviewed: Jun 29, 2018)	569129	Hereditary_cancer-predisposing_syndrome|DICER1-related_pleuropulmonary_blastoma_cancer_predisposition_syndrome	criteria_provided, multiple_submitters, no_conflicts
14	95113129	T	G	DICER1	NM_177438.2:c.2003A>C	NP_803187.1:p.Tyr668Ser	missense	LB	5.42E-06	.	.	T	24	0.223	.	.	.	.
14	95113146	T	A	DICER1	NM_177438.2:c.1986A>T	NP_803187.1:p.Thr662=	synonymous	LB	5.42E-06	.	0	.	.	.	Likely_benign (Last reviewed: Dec 31, 2019)	463819	Hereditary_cancer-predisposing_syndrome|not_provided	criteria_provided, multiple_submitters, no_conflicts
14	95113153	T	C	DICER1	NM_177438.2:c.1979A>G	NP_803187.1:p.Asp660Gly	missense	LB	5.42E-06	.	0	T	19.47	0.143	Uncertain_significance (Last reviewed: Dec 30, 2019)	477143	Hereditary_cancer-predisposing_syndrome|DICER1-related_pleuropulmonary_blastoma_cancer_predisposition_syndrome	criteria_provided, multiple_submitters, no_conflicts
14	95113173	G	A	DICER1	NM_177438.2:c.1959C>T	NP_803187.1:p.Cys653=	synonymous	LB	1.08E-05	.	.	.	.	.	.	.	.	.
14	95113179	A	G	DICER1	NM_177438.2:c.1953T>C	NP_803187.1:p.Pro651=	synonymous	LB	1.08E-05	.	0	.	.	.	Benign/Likely_benign (Last reviewed: Dec 31, 2019)	400442	Hereditary_cancer-predisposing_syndrome|not_provided	criteria_provided, multiple_submitters, no_conflicts
14	95115664	T	A	DICER1	NM_177438.2:c.1907+3A>T		splice_region	VUS	1.63E-05	.	.	.	.	.	Likely_benign (Last reviewed: Jan 7, 2020)	815597	Hereditary_cancer-predisposing_syndrome	criteria_provided, single_submitter
14	95115670	T	C	DICER1	NM_177438.2:c.1904A>G	NP_803187.1:p.Asn635Ser	missense	LB	1.63E-05	.	1.76E-05	T	24.6	0.398	Uncertain_significance (Last reviewed: Dec 22, 2018)	477748	Hereditary_cancer-predisposing_syndrome|DICER1-related_pleuropulmonary_blastoma_cancer_predisposition_syndrome	criteria_provided, multiple_submitters, no_conflicts
14	95115677	G	A	DICER1	NM_177438.2:c.1897C>T	NP_803187.1:p.His633Tyr	missense	LB	5.42E-06	.	.	T	25.4	0.324	Uncertain_significance (Last reviewed: Jun 16, 2019)	812139	Hereditary_cancer-predisposing_syndrome	criteria_provided, single_submitter
14	95115684	G	A	DICER1	NM_177438.2:c.1890C>T	NP_803187.1:p.Ala630=	synonymous	LB	5.42E-06	.	0	.	.	.	Likely_benign (Last reviewed: Dec 31, 2019)	528506	Hereditary_cancer-predisposing_syndrome|not_provided	criteria_provided, multiple_submitters, no_conflicts
14	95115687	C	T	DICER1	NM_177438.2:c.1887G>A	NP_803187.1:p.Thr629=	synonymous	LB	0.0004821	5.52E-05	5.28E-05	.	.	.	Benign/Likely_benign (Last reviewed: Dec 31, 2019)	241979	Pleuropulmonary_blastoma|Hereditary_cancer-predisposing_syndrome|not_specified|not_provided	criteria_provided, multiple_submitters, no_conflicts
14	95115688	G	A	DICER1	NM_177438.2:c.1886C>T	NP_803187.1:p.Thr629Met	missense	LB	1.63E-05	1.84E-05	2.64E-05	T	33	0.393	Uncertain_significance (Last reviewed: Oct 19, 2019)	464411	Hereditary_cancer-predisposing_syndrome|DICER1-related_pleuropulmonary_blastoma_cancer_predisposition_syndrome	criteria_provided, multiple_submitters, no_conflicts
14	95115691	T	C	DICER1	NM_177438.2:c.1883A>G	NP_803187.1:p.Asn628Ser	missense	LB	5.42E-06	3.68E-05	8.79E-06	T	22.1	0.131	Uncertain_significance (Last reviewed: Dec 15, 2019)	400105	Hereditary_cancer-predisposing_syndrome|DICER1-related_pleuropulmonary_blastoma_cancer_predisposition_syndrome	criteria_provided, multiple_submitters, no_conflicts
14	95115717	G	A	DICER1	NM_177438.2:c.1857C>T	NP_803187.1:p.Asp619=	synonymous	LB	1.08E-05	.	2.64E-05	.	.	.	Likely_benign (Last reviewed: Dec 31, 2019)	400730	Hereditary_cancer-predisposing_syndrome|not_provided	criteria_provided, multiple_submitters, no_conflicts
14	95115721	G	A	DICER1	NM_177438.2:c.1853C>T	NP_803187.1:p.Pro618Leu	missense	LB	5.42E-06	.	.	T	21.1	0.065	Uncertain_significance (Last reviewed: Aug 20, 2018)	642907	DICER1-related_pleuropulmonary_blastoma_cancer_predisposition_syndrome	criteria_provided, single_submitter
14	95115731	C	A	DICER1	NM_177438.2:c.1843G>T	NP_803187.1:p.Val615Leu	missense	LB	2.17E-05	.	8.79E-06	T	23.7	0.149	.	.	.	.
14	95115735	T	C	DICER1	NM_177438.2:c.1839A>G	NP_803187.1:p.Pro613=	synonymous	LB	1.08E-05	.	0	.	.	.	Likely_benign (Last reviewed: Dec 31, 2019)	463827	Hereditary_cancer-predisposing_syndrome|not_provided	criteria_provided, multiple_submitters, no_conflicts
14	95115747	G	A	DICER1	NM_177438.2:c.1827C>T	NP_803187.1:p.Asp609=	synonymous	LB	2.71E-05	3.68E-05	2.64E-05	.	.	.	Likely_benign (Last reviewed: Dec 31, 2019)	400452	Hereditary_cancer-predisposing_syndrome|not_provided	criteria_provided, multiple_submitters, no_conflicts
14	95115749	C	A	DICER1	NM_177438.2:c.1825G>T	NP_803187.1:p.Asp609Tyr	missense	LB	0.0001246	5.52E-05	2.64E-05	T	23.4	0.219	Conflicting_interpretations_of_pathogenicity (Last reviewed: Dec 31, 2019)	137704	Pleuropulmonary_blastoma|Hereditary_cancer-predisposing_syndrome|not_specified|not_provided	criteria_provided, conflicting_interpretations
14	95115751	T	C	DICER1	NM_177438.2:c.1823A>G	NP_803187.1:p.Asp608Gly	missense	LB	5.42E-06	.	8.79E-06	T	24.2	0.171	Uncertain_significance (Last reviewed: Sep 18, 2018)	464662	Hereditary_cancer-predisposing_syndrome|DICER1-related_pleuropulmonary_blastoma_cancer_predisposition_syndrome	criteria_provided, multiple_submitters, no_conflicts

© 2021 Mirshahi UL et al. *JAMA Network Open.*

14	95115761	T	C	DICER1	NM_177438.2:c.1813A>G	NP_803187.1:p.Met605Val	missense	LB	3.79E-05	5.52E-05	2.64E-05	T	4.319	0.06	Conflicting_interpretations_of_pathogenicity (Last reviewed: Dec 28, 2018)	464674	Hereditary_cancer-predisposing_syndrome|DICER1-related_pleuropulmonary_blastoma_cancer_predisposition_syndrome	criteria_provided, conflicting_interpretations

© 2021 Mirshahi UL et al. *JAMA Network Open.*

14	95115772	A	G	DICER1	NM_177438.2:c.1802T>C	NP_803187.1:p.Ile601Thr	missense	LB	1.08E-05	.	8.79E-06	T	1.795	0.033	Uncertain_significance (Last reviewed: Nov 23, 2019)	528889	Hereditary_cancer-predisposing_syndrome|DICER1-related_pleuropulmonary_blastoma_cancer_predisposition_syndrome	criteria_provided, multiple_submitters, no_conflicts
14	95115774	G	A	DICER1	NM_177438.2:c.1800C>T	NP_803187.1:p.Asp600=	synonymous	LB	2.17E-05	3.68E-05	4.40E-05	.	.	.	Likely_benign (Last reviewed: Dec 31, 2019)	463835	Hereditary_cancer-predisposing_syndrome|not_provided	criteria_provided, multiple_submitters, no_conflicts
14	95115782	C	T	DICER1	NM_177438.2:c.1792G>A	NP_803187.1:p.Glu598Lys	missense	LB	1.08E-05	0.0001	6.15E-05	T	23.4	0.126	Uncertain_significance (Last reviewed: Dec 6, 2019)	642909	Hereditary_cancer-predisposing_syndrome|DICER1-related_pleuropulmonary_blastoma_cancer_predisposition_syndrome	criteria_provided, multiple_submitters, no_conflicts
14	95115804	A	G	DICER1	NM_177438.2:c.1770T>C	NP_803187.1:p.Cys590=	synonymous	LB	5.42E-06	.	8.79E-06	.	.	.	Likely_benign (Last reviewed: Dec 31, 2019)	400117	Hereditary_cancer-predisposing_syndrome|not_provided	criteria_provided, multiple_submitters, no_conflicts
14	95115805	C	A	DICER1	NM_177438.2:c.1769G>T	NP_803187.1:p.Cys590Phe	missense	LB	3.79E-05	.	.	T	26.4	0.349	Uncertain_significance (Last reviewed: Nov 19, 2019)	477362	Hereditary_cancer-predisposing_syndrome|DICER1-related_pleuropulmonary_blastoma_cancer_predisposition_syndrome	criteria_provided, multiple_submitters, no_conflicts
14	95115807	C	T	DICER1	NM_177438.2:c.1767G>A	NP_803187.1:p.Lys589=	synonymous	LB	5.42E-06	.	.	.	.	.	Likely_benign (Last reviewed: Dec 31, 2019)	400124	Hereditary_cancer-predisposing_syndrome|not_provided	criteria_provided, multiple_submitters, no_conflicts
14	95115816	C	T	DICER1	NM_177438.2:c.1758G>A	NP_803187.1:p.Leu586=	synonymous	LB	2.71E-05	.	2.64E-05	.	.	.	Likely_benign (Last reviewed: Dec 31, 2019)	400455	Hereditary_cancer-predisposing_syndrome|not_provided	criteria_provided, multiple_submitters, no_conflicts
14	95116469	T	C	DICER1	NM_177438.2:c.1736A>G	NP_803187.1:p.Tyr579Cys	missense	LB	5.42E-06	1.85E-05	8.82E-06	T	26.3	0.615	Uncertain_significance (Last reviewed: Mar 28, 2019)	463862	Hereditary_cancer-predisposing_syndrome|DICER1-related_pleuropulmonary_blastoma_cancer_predisposition_syndrome	criteria_provided, multiple_submitters, no_conflicts
14	95116480	GTCT	G	DICER1	NM_177438.2:c.1722_1725delinsC	NP_803187.1:p.Glu574del	inframe_deletion	VUS	2.71E-05	.	.	.	.	.	.	.	.	.
14	95116497	T	C	DICER1	NM_177438.2:c.1708A>G	NP_803187.1:p.Lys570Glu	missense	LB	5.42E-06	.	.	T	12.17	0.154	.	.	.	.
14	95116498	T	A	DICER1	NM_177438.2:c.1707A>T	NP_803187.1:p.Ile569=	synonymous	LB	5.42E-06	.	1.76E-05	.	.	.	Likely_benign (Last reviewed: Dec 31, 2019)	241981	Hereditary_cancer-predisposing_syndrome|not_provided	criteria_provided, multiple_submitters, no_conflicts
14	95116504	G	C	DICER1	NM_177438.2:c.1701C>G	NP_803187.1:p.Asp567Glu	missense	LB	5.42E-06	.	.	T	7.841	0.049	.	.	.	.
14	95116513	C	T	DICER1	NM_177438.2:c.1692G>A	NP_803187.1:p.Ala564=	synonymous	LB	9.21E-05	5.53E-05	3.52E-05	.	.	.	Benign/Likely_benign (Last reviewed: Dec 31, 2019)	241982	Hereditary_cancer-predisposing_syndrome|DICER1-related_pleuropulmonary_blastoma_cancer_predisposition_syndrome|not_specified|not_provided	criteria_provided, multiple_submitters, no_conflicts
14	95116514	G	A	DICER1	NM_177438.2:c.1691C>T	NP_803187.1:p.Ala564Val	missense	LB	3.79E-05	9.21E-05	6.16E-05	T	23.6	0.344	Uncertain_significance (Last reviewed: Dec 23, 2019)	399947	Hereditary_cancer-predisposing_syndrome|DICER1-related_pleuropulmonary_blastoma_cancer_predisposition_syndrome	criteria_provided, multiple_submitters, no_conflicts
14	95116523	A	G	DICER1	NM_177438.2:c.1682T>C	NP_803187.1:p.Ile561Thr	missense	LB	5.42E-06	.	1.76E-05	T	23.6	0.407	Uncertain_significance (Last reviewed: Mar 13, 2017)	463867	DICER1-related_pleuropulmonary_blastoma_cancer_predisposition_syndrome	criteria_provided, single_submitter
14	95116524	T	C	DICER1	NM_177438.2:c.1681A>G	NP_803187.1:p.Ile561Val	missense	LB	5.42E-06	.	8.80E-06	T	4.528	0.051	Conflicting_interpretations_of_pathogenicity (Last reviewed: Oct 6, 2018)	241983	Pleuropulmonary_blastoma|Hereditary_cancer-predisposing_syndrome|DICER1-related_pleuropulmonary_blastoma_cancer_predisposition_syndrome	criteria_provided, conflicting_interpretations
14	95116540	T	C	DICER1	NM_177438.2:c.1665A>G	NP_803187.1:p.Ala555=	synonymous	LB	1.63E-05	.	.	.	.	.	Likely_benign (Last reviewed: Dec 31, 2019)	528911	not_provided	criteria_provided, single_submitter
14	95116559	G	T	DICER1	NM_177438.2:c.1646C>A	NP_803187.1:p.Ser549Tyr	missense	pDeleterious	5.42E-06	.	.	D	28.6	0.821	.	.	.	.
14	95116598	C	T	DICER1	NM_177438.2:c.1607G>A	NP_803187.1:p.Arg536His	missense	LB	5.42E-06	.	.	T	34	0.729	.	.	.	.
14	95116599	G	A	DICER1	NM_177438.2:c.1606C>T	NP_803187.1:p.Arg536Cys	missense	LB	5.42E-06	.	8.80E-06	T	34	0.659	Uncertain_significance (Last reviewed: Nov 4, 2019)	477218	Hereditary_cancer-predisposing_syndrome	criteria_provided, single_submitter
14	95116618	T	C	DICER1	NM_177438.2:c.1587A>G	NP_803187.1:p.Pro529=	synonymous	LB	5.42E-06	.	.	.	.	.	.	.	.	.
14	95116629	C	T	DICER1	NM_177438.2:c.1576G>A	NP_803187.1:p.Val526Ile	missense	LB	5.42E-06	.	8.80E-06	T	18.77	0.335	Uncertain_significance (Last reviewed: Dec 11, 2019)	399949	Hereditary_cancer-predisposing_syndrome|DICER1-related_pleuropulmonary_blastoma_cancer_predisposition_syndrome	criteria_provided, multiple_submitters, no_conflicts
14	95116662	G	A	DICER1	NM_177438.2:c.1543C>T	NP_803187.1:p.Leu515=	synonymous	LB	0.0001679	0.0002	0.0002	.	.	.	Likely_benign (Last reviewed: Dec 31, 2019)	241985	Hereditary_cancer-predisposing_syndrome|not_provided	criteria_provided, multiple_submitters, no_conflicts
14	95116665	T	C	DICER1	NM_177438.2:c.1540A>G	NP_803187.1:p.Asn514Asp	missense	LB	5.42E-06	.	.	T	24.2	0.654	.	.	.	.
14	95116680	G	A	DICER1	NM_177438.2:c.1525C>T	NP_803187.1:p.Arg509Ter	stop_gained	pLOF	1.08E-05	.	.	.	36	.	Pathogenic (Last reviewed: Sep 20, 2019)	248876	Hereditary_cancer-predisposing_syndrome|DICER1-related_pleuropulmonary_blastoma_cancer_predisposition_syndrome|not_provided	criteria_provided, multiple_submitters, no_conflicts
14	95117640	T	C	DICER1	NM_177438.2:c.1491A>G	NP_803187.1:p.Glu497=	synonymous	LB	5.42E-06	3.68E-05	1.76E-05	.	.	.	Likely_benign (Last reviewed: Sep 21, 2017)	477225	Hereditary_cancer-predisposing_syndrome	criteria_provided, single_submitter
14	95117645	C	T	DICER1	NM_177438.2:c.1486G>A	NP_803187.1:p.Ala496Thr	missense	LB	1.08E-05	3.68E-05	1.76E-05	T	22.9	0.111	.	.	.	.
14	95117646	T	C	DICER1	NM_177438.2:c.1485A>G	NP_803187.1:p.Glu495=	synonymous	LB	1.08E-05	.	.	.	.	.	.	.	.	.
14	95117662	C	T	DICER1	NM_177438.2:c.1469G>A	NP_803187.1:p.Arg490His	missense	LB	7.04E-05	.	0	T	25.7	0.247	Uncertain_significance (Last reviewed: Nov 29, 2018)	464712	Hereditary_cancer-predisposing_syndrome|DICER1-related_pleuropulmonary_blastoma_cancer_predisposition_syndrome	criteria_provided, multiple_submitters, no_conflicts
14	95117663	G	A	DICER1	NM_177438.2:c.1468C>T	NP_803187.1:p.Arg490Cys	missense	LB	0.0001463	1.84E-05	1.76E-05	T	28.7	0.502	Uncertain_significance (Last reviewed: Nov 26, 2019)	241991	Hereditary_cancer-predisposing_syndrome|DICER1-related_pleuropulmonary_blastoma_cancer_predisposition_syndrome	criteria_provided, multiple_submitters, no_conflicts
14	95117667	C	A	DICER1	NM_177438.2:c.1464G>T	NP_803187.1:p.Gln488His	missense	LB	5.42E-06	.	0	T	24.7	0.356	.	.	.	.
14	95117686	T	C	DICER1	NM_177438.2:c.1445A>G	NP_803187.1:p.His482Arg	missense	LB	1.63E-05	1.84E-05	8.80E-06	T	22.4	0.516	Uncertain_significance (Last reviewed: Nov 8, 2019)	528977	Hereditary_cancer-predisposing_syndrome|DICER1-related_pleuropulmonary_blastoma_cancer_predisposition_syndrome	criteria_provided, multiple_submitters, no_conflicts
14	95117691	A	G	DICER1	NM_177438.2:c.1440T>C	NP_803187.1:p.Thr480=	synonymous	LB	5.42E-05	.	.	.	.	.	Likely_benign (Last reviewed: Dec 31, 2019)	477751	Hereditary_cancer-predisposing_syndrome|not_provided	criteria_provided, multiple_submitters, no_conflicts

© 2021 Mirshahi UL et al. *JAMA Network Open.*

14	95117701	T	C	DICER1	NM_177438.2:c.1430A>G	NP_803187.1:p.Asn477Ser	missense	LB	1.08E-05	.	8.80E-06	T	22.5	0.293	Uncertain_significance (Last reviewed: Dec 17, 2019)	399957	Hereditary_cancer-predisposing_syndrome|DICER1-related_pleuropulmonary_blastoma_cancer_predisposition_syndrome	criteria_provided, multiple_submitters, no_conflicts
14	95117726	G	C	DICER1	NM_177438.2:c.1405C>G	NP_803187.1:p.Pro469Ala	missense	LB	5.42E-06	.	.	T	18.67	0.251	.	.	.	.

© 2021 Mirshahi UL et al. *JAMA Network Open.*

14	95117750	T	C	DICER1	NM_177438.2:c.1381A>G	NP_803187.1:p.Ile461Val	missense	LB	0.0003196	0.0001	0.0002	T	23.3	0.286	Conflicting_interpretations_of_pathogenicity (Last reviewed: Dec 31, 2019)	241992	Pleuropulmonary_blastoma|Hereditary_cancer-predisposing_syndrome|not_provided	criteria_provided, conflicting_interpretations
14	95124255	G	C	DICER1	NM_177438.2:c.1317C>G	NP_803187.1:p.Thr439=	synonymous	LB	2.71E-05	5.52E-05	7.07E-05	.	.	.	Likely_benign (Last reviewed: Dec 31, 2019)	241993	Hereditary_cancer-predisposing_syndrome|not_provided	criteria_provided, multiple_submitters, no_conflicts
14	95124275	T	G	DICER1	NM_177438.2:c.1297A>C	NP_803187.1:p.Asn433His	missense	LB	5.42E-06	.	.	T	24	0.213	Uncertain_significance (Last reviewed: Dec 2, 2019)	812167	Hereditary_cancer-predisposing_syndrome	criteria_provided, single_submitter
14	95124309	A	G	DICER1	NM_177438.2:c.1263T>C	NP_803187.1:p.Asp421=	synonymous	LB	1.63E-05	.	.	.	.	.	.	.	.	.
14	95124318	A	G	DICER1	NM_177438.2:c.1254T>C	NP_803187.1:p.Asp418=	synonymous	LB	1.63E-05	.	8.80E-06	.	.	.	Likely_benign (Last reviewed: Dec 31, 2019)	400477	Hereditary_cancer-predisposing_syndrome|not_provided	criteria_provided, multiple_submitters, no_conflicts
14	95124321	A	C	DICER1	NM_177438.2:c.1251T>G	NP_803187.1:p.Asp417Glu	missense	LB	5.42E-06	.	.	T	22.5	0.144	Uncertain_significance (Last reviewed: Aug 15, 2017)	464724	DICER1-related_pleuropulmonary_blastoma_cancer_predisposition_syndrome	criteria_provided, single_submitter
14	95124339	T	C	DICER1	NM_177438.2:c.1233A>G	NP_803187.1:p.Ser411=	synonymous	LB	3.25E-05	.	0	.	.	.	Benign/Likely_benign (Last reviewed: Dec 31, 2019)	241995	Hereditary_cancer-predisposing_syndrome|not_provided	criteria_provided, multiple_submitters, no_conflicts
14	95124359	T	G	DICER1	NM_177438.2:c.1213A>C	NP_803187.1:p.Asn405His	missense	LB	5.42E-06	1.84E-05	1.76E-05	T	25.2	0.159	Uncertain_significance (Last reviewed: Dec 3, 2019)	464447	Hereditary_cancer-predisposing_syndrome|DICER1-related_pleuropulmonary_blastoma_cancer_predisposition_syndrome	criteria_provided, multiple_submitters, no_conflicts
14	95124377	C	G	DICER1	NM_177438.2:c.1195G>C	NP_803187.1:p.Glu399Gln	missense	LB	5.42E-06	.	.	T	23.6	0.175	Uncertain_significance (Last reviewed: Oct 13, 2018)	642928	DICER1-related_pleuropulmonary_blastoma_cancer_predisposition_syndrome	criteria_provided, single_submitter
14	95124380	C	T	DICER1	NM_177438.2:c.1192G>A	NP_803187.1:p.Val398Ile	missense	LB	1.63E-05	.	8.80E-06	T	25.1	0.156	Uncertain_significance (Last reviewed: Jun 28, 2018)	569139	DICER1-related_pleuropulmonary_blastoma_cancer_predisposition_syndrome	criteria_provided, single_submitter
14	95124381	G	A	DICER1	NM_177438.2:c.1191C>T	NP_803187.1:p.Ser397=	synonymous	LB	1.08E-05	1.84E-05	1.76E-05	.	.	.	Likely_benign (Last reviewed: Dec 31, 2019)	222411	Hereditary_cancer-predisposing_syndrome|not_provided	criteria_provided, multiple_submitters, no_conflicts
14	95124397	C	T	DICER1	NM_177438.2:c.1175G>A	NP_803187.1:p.Arg392Gln	missense	LB	5.42E-06	1.84E-05	1.76E-05	T	26.3	0.182	Uncertain_significance (Last reviewed: Apr 19, 2019)	573082	Hereditary_cancer-predisposing_syndrome|DICER1-related_pleuropulmonary_blastoma_cancer_predisposition_syndrome	criteria_provided, multiple_submitters, no_conflicts
14	95124399	C	A	DICER1	NM_177438.2:c.1173G>T	NP_803187.1:p.Glu391Asp	missense	LB	5.42E-06	.	8.80E-06	T	22.9	0.302	.	.	.	.
14	95124402	A	G	DICER1	NM_177438.2:c.1170T>C	NP_803187.1:p.Tyr390=	synonymous	LB	5.42E-06	.	0	.	.	.	Likely_benign (Last reviewed: Dec 31, 2019)	477373	Hereditary_cancer-predisposing_syndrome|not_provided	criteria_provided, multiple_submitters, no_conflicts
14	95124404	A	G	DICER1	NM_177438.2:c.1168T>C	NP_803187.1:p.Tyr390His	missense	LB	1.08E-05	.	.	T	14.6	0.162	Conflicting_interpretations_of_pathogenicity (Last reviewed: Dec 31, 2019)	241996	Hereditary_cancer-predisposing_syndrome|not_provided	criteria_provided, conflicting_interpretations
14	95124428	C	T	DICER1	NM_177438.2:c.1144G>A	NP_803187.1:p.Glu382Lys	missense	LB	5.42E-06	.	0	T	29.9	0.304	.	.	.	.
14	95124448	G	C	DICER1	NM_177438.2:c.1124C>G	NP_803187.1:p.Pro375Arg	missense	LB	0.0004388	0.0004	0.0005	T	25.6	0.578	Conflicting_interpretations_of_pathogenicity (Last reviewed: Jan 8, 2020)	241997	Neuroblastoma|Pleuropulmonary_blastoma|Hereditary_cancer-predisposing_syndrome|DICER1-related_pleuropulmonary_blastoma_cancer_predisposition_syndrome	criteria_provided, conflicting_interpretations
14	95124453	T	G	DICER1	NM_177438.2:c.1119A>C	NP_803187.1:p.Val373=	synonymous	LB	5.42E-06	.	.	.	.	.	Likely_benign (Last reviewed: Dec 31, 2019)	688382	Hereditary_cancer-predisposing_syndrome|not_provided	criteria_provided, multiple_submitters, no_conflicts
14	95124468	A	G	DICER1	NM_177438.2:c.1104T>C	NP_803187.1:p.Leu368=	synonymous	LB	5.42E-06	.	.	.	.	.	.	.	.	.
14	95124481	G	A	DICER1	NM_177438.2:c.1091C>T	NP_803187.1:p.Ser364Leu	missense	LB	5.42E-06	.	.	T	25.7	0.295	Uncertain_significance (Last reviewed: Mar 3, 2019)	528996	DICER1-related_pleuropulmonary_blastoma_cancer_predisposition_syndrome	criteria_provided, single_submitter
14	95124491	C	T	DICER1	NM_177438.2:c.1081G>A	NP_803187.1:p.Glu361Lys	missense	LB	9.75E-05	.	8.80E-06	T	25.4	0.215	Uncertain_significance (Last reviewed: Nov 26, 2019)	242000	Hereditary_cancer-predisposing_syndrome|DICER1-related_pleuropulmonary_blastoma_cancer_predisposition_syndrome	criteria_provided, multiple_submitters, no_conflicts
14	95124503	C	T	DICER1	NM_177438.2:c.1069G>A	NP_803187.1:p.Ala357Thr	missense	LB	2.17E-05	.	8.81E-06	T	22.8	0.182	Uncertain_significance (Last reviewed: Dec 1, 2018)	566779	Hereditary_cancer-predisposing_syndrome|DICER1-related_pleuropulmonary_blastoma_cancer_predisposition_syndrome	criteria_provided, multiple_submitters, no_conflicts
14	95124505	T	C	DICER1	NM_177438.2:c.1067A>G	NP_803187.1:p.His356Arg	missense	LB	4.88E-05	.	.	T	15.06	0.2	Uncertain_significance (Last reviewed: Oct 24, 2019)	477184	Hereditary_cancer-predisposing_syndrome	criteria_provided, single_submitter
14	95124540	A	C	DICER1	NM_177438.2:c.1032T>G	NP_803187.1:p.Phe344Leu	missense	LB	5.42E-06	.	8.88E-06	T	5.168	0.316	Uncertain_significance (Last reviewed: May 31, 2017)	464725	DICER1-related_pleuropulmonary_blastoma_cancer_predisposition_syndrome	criteria_provided, single_submitter
14	95124542	A	AT	DICER1	NM_177438.2:c.1030delinsAT	NP_803187.1:p.Phe344Ilefs	frameshift	pLOF	1.08E-05	.	.	.	.	.	.	.	.	.
14	95124548	TG	T	DICER1	NM_177438.2:c.1023_1024delinsA	NP_803187.1:p.His341Glnfs	frameshift	pLOF	5.42E-06	.	.	.	.	.	.	.	.	.
14	95124576	G	A	DICER1	NM_177438.2:c.996C>T	NP_803187.1:p.Tyr332=	synonymous	LB	1.08E-05	.	8.99E-06	.	.	.	Benign/Likely_benign (Last reviewed: Dec 31, 2019)	400166	Hereditary_cancer-predisposing_syndrome|not_provided	criteria_provided, multiple_submitters, no_conflicts
14	95124599	T	C	DICER1	NM_177438.2:c.973A>G	NP_803187.1:p.Met325Val	missense	LB	1.08E-05	.	.	T	14.59	0.181	Uncertain_significance (Last reviewed: Aug 28, 2017)	528552	DICER1-related_pleuropulmonary_blastoma_cancer_predisposition_syndrome	criteria_provided, single_submitter
14	95124600	C	T	DICER1	NM_177438.2:c.972G>A	NP_803187.1:p.Met324Ile	missense	LB	5.42E-06	.	.	T	14.83	0.098	Uncertain_significance (Last reviewed: Apr 4, 2018)	528496	Hereditary_cancer-predisposing_syndrome|DICER1-related_pleuropulmonary_blastoma_cancer_predisposition_syndrome	criteria_provided, multiple_submitters, no_conflicts
14	95124613	T	C	DICER1	NM_177438.2:c.959A>G	NP_803187.1:p.Lys320Arg	missense	LB	1.08E-05	.	9.06E-06	T	21.9	0.177	Uncertain_significance (Last reviewed: Jun 12, 2016)	400483	DICER1-related_pleuropulmonary_blastoma_cancer_predisposition_syndrome	criteria_provided, single_submitter
14	95124622	C	T	DICER1	NM_177438.2:c.950G>A	NP_803187.1:p.Cys317Tyr	missense	LB	5.42E-06	.	.	T	28.6	0.584	.	.	.	.
14	95124647	C	T	DICER1	NM_177438.2:c.925G>A	NP_803187.1:p.Val309Ile	missense	LB	2.71E-05	.	0	T	25	0.189	Uncertain_significance (Last reviewed: Apr 19, 2019)	464703	Hereditary_cancer-predisposing_syndrome|DICER1-related_pleuropulmonary_blastoma_cancer_predisposition_syndrome	criteria_provided, multiple_submitters, no_conflicts
14	95124648	G	A	DICER1	NM_177438.2:c.924C>T	NP_803187.1:p.Ala308=	synonymous	LB	5.42E-05	.	9.13E-06	.	.	.	Benign/Likely_benign (Last reviewed: Dec 31, 2019)	222412	Hereditary_cancer-predisposing_syndrome|not_provided	criteria_provided, multiple_submitters, no_conflicts

© 2021 Mirshahi UL et al. *JAMA Network Open.*

14	95124652	C	T	DICER1	NM_177438.2:c.920G>A	NP_803187.1:p.Arg307His	missense	LB	1.63E-05	1.85E-05	3.66E-05	T	25.9	0.112	Uncertain_significance (Last reviewed: Dec 22, 2019)	242001	Hereditary_cancer-predisposing_syndrome|DICER1-related_pleuropulmonary_blastoma_cancer_predisposition_syndrome	criteria_provided, multiple_submitters, no_conflicts
14	95124654	A	G	DICER1	NM_177438.2:c.918T>C	NP_803187.1:p.Cys306=	synonymous	LB	0.0001246	.	.	.	.	.	Likely_benign (Last reviewed: Dec 31, 2019)	399969	Hereditary_cancer-predisposing_syndrome|not_provided	criteria_provided, multiple_submitters, no_conflicts
14	95124667	A	G	DICER1	NM_177438.2:c.905T>C	NP_803187.1:p.Ile302Thr	missense	LB	1.08E-05	.	.	T	19.37	0.237	.	.	.	.
14	95126586	C	T	DICER1	NM_177438.2:c.897G>A	NP_803187.1:p.Ser299=	synonymous	LB	8.67E-05	5.57E-05	0.0001	.	.	.	Likely_benign (Last reviewed: Dec 31, 2019)	321940	Pleuropulmonary_blastoma|Hereditary_cancer-predisposing_syndrome|not_provided	criteria_provided, multiple_submitters, no_conflicts
14	95126587	G	A	DICER1	NM_177438.2:c.896C>T	NP_803187.1:p.Ser299Leu	missense	LB	0.0003089	5.56E-05	4.41E-05	T	26.3	0.233	Uncertain_significance (Last reviewed: Dec 24, 2019)	242002	Hereditary_cancer-predisposing_syndrome|DICER1-related_pleuropulmonary_blastoma_cancer_predisposition_syndrome	criteria_provided, multiple_submitters, no_conflicts
14	95126599	G	C	DICER1	NM_177438.2:c.884C>G	NP_803187.1:p.Ser295Cys	missense	LB	1.08E-05	1.85E-05	0	T	27.4	0.159	Conflicting_interpretations_of_pathogenicity (Last reviewed: Dec 31, 2019)	242003	Hereditary_cancer-predisposing_syndrome|not_specified|not_provided	criteria_provided, conflicting_interpretations
14	95126606	T	C	DICER1	NM_177438.2:c.877A>G	NP_803187.1:p.Arg293Gly	missense	LB	1.08E-05	.	8.81E-06	T	14.27	0.077	Uncertain_significance (Last reviewed: Feb 18, 2019)	242004	Hereditary_cancer-predisposing_syndrome|DICER1-related_pleuropulmonary_blastoma_cancer_predisposition_syndrome	criteria_provided, multiple_submitters, no_conflicts
14	95126627	T	C	DICER1	NM_177438.2:c.856A>G	NP_803187.1:p.Ile286Val	missense	LB	5.42E-06	.	.	T	4.578	0.034	.	.	.	.
14	95126629	T	C	DICER1	NM_177438.2:c.854A>G	NP_803187.1:p.Asn285Ser	missense	LB	1.08E-05	5.54E-05	2.64E-05	T	23.1	0.119	Uncertain_significance (Last reviewed: Dec 13, 2018)	477781	Hereditary_cancer-predisposing_syndrome	criteria_provided, single_submitter
14	95126637	A	G	DICER1	NM_177438.2:c.846T>C	NP_803187.1:p.Asn282=	synonymous	LB	5.42E-06	.	8.80E-06	.	.	.	Likely_benign (Last reviewed: Jan 10, 2018)	812185	Hereditary_cancer-predisposing_syndrome	criteria_provided, single_submitter
14	95126673	C	T	DICER1	NM_177438.2:c.810G>A	NP_803187.1:p.Leu270=	synonymous	LB	1.08E-05	.	.	.	.	.	Likely_benign (Last reviewed: Dec 31, 2019)	739432	not_provided	criteria_provided, single_submitter
14	95126675	G	A	DICER1	NM_177438.2:c.808C>T	NP_803187.1:p.Leu270=	synonymous	LB	1.08E-05	.	0	.	.	.	.	.	.	.
14	95126706	T	C	DICER1	NM_177438.2:c.777A>G	NP_803187.1:p.Pro259=	synonymous	LB	1.63E-05	.	.	.	.	.	.	.	.	.
14	95126707	G	A	DICER1	NM_177438.2:c.776C>T	NP_803187.1:p.Pro259Leu	missense	LB	2.17E-05	1.84E-05	8.80E-06	T	21.2	0.197	Uncertain_significance (Last reviewed: Nov 14, 2019)	463914	Hereditary_cancer-predisposing_syndrome|DICER1-related_pleuropulmonary_blastoma_cancer_predisposition_syndrome	criteria_provided, multiple_submitters, no_conflicts
14	95126710	C	A	DICER1	NM_177438.2:c.773G>T	NP_803187.1:p.Gly258Val	missense	LB	5.96E-05	0.0003	0.0002	T	25.2	0.321	Conflicting_interpretations_of_pathogenicity (Last reviewed: Dec 4, 2018)	321947	Pleuropulmonary_blastoma|Hereditary_cancer-predisposing_syndrome|DICER1-related_pleuropulmonary_blastoma_cancer_predisposition_syndrome	criteria_provided, conflicting_interpretations
14	95126711	C	A	DICER1	NM_177438.2:c.772G>T	NP_803187.1:p.Gly258Ter	stop_gained	LB	5.42E-06	.	.	.	38	.	.	.	.	.
14	95126718	C	T	DICER1	NM_177438.2:c.765G>A	NP_803187.1:p.Val255=	synonymous	LB	5.42E-06	.	.	.	.	.	.	.	.	.
14	95126731	C	T	DICER1	NM_177438.2:c.752G>A	NP_803187.1:p.Cys251Tyr	missense	LB	5.42E-06	.	0	T	22.5	0.074	.	.	.	.
14	95126736	C	T	DICER1	NM_177438.2:c.747G>A	NP_803187.1:p.Gln249=	synonymous	LB	0.00013	3.69E-05	6.16E-05	.	.	.	Likely_benign (Last reviewed: Dec 31, 2019)	242005	Hereditary_cancer-predisposing_syndrome|not_provided	criteria_provided, multiple_submitters, no_conflicts
14	95126744	T	C	DICER1	NM_177438.2:c.739A>G	NP_803187.1:p.Thr247Ala	missense	LB	6.50E-05	3.69E-05	1.76E-05	T	7.765	0.122	Uncertain_significance (Last reviewed: Dec 16, 2019)	464472	Hereditary_cancer-predisposing_syndrome|DICER1-related_pleuropulmonary_blastoma_cancer_predisposition_syndrome	criteria_provided, multiple_submitters, no_conflicts
14	95129477	T	C	DICER1	NM_177438.2:c.729A>G	NP_803187.1:p.Leu243=	synonymous	LB	5.42E-06	.	8.80E-06	.	.	.	Likely_benign (Last reviewed: Dec 31, 2019)	463920	Hereditary_cancer-predisposing_syndrome|not_provided	criteria_provided, multiple_submitters, no_conflicts
14	95129479	A	G	DICER1	NM_177438.2:c.727T>C	NP_803187.1:p.Leu243=	synonymous	LB	5.42E-06	.	.	.	.	.	.	.	.	.
14	95129508	T	C	DICER1	NM_177438.2:c.698A>G	NP_803187.1:p.Asn233Ser	missense	LB	5.42E-06	.	8.80E-06	T	0.789	0.077	Uncertain_significance (Last reviewed: Mar 30, 2019)	812197	Hereditary_cancer-predisposing_syndrome	criteria_provided, single_submitter
14	95129536	G	C	DICER1	NM_177438.2:c.670C>G	NP_803187.1:p.Gln224Glu	missense	LB	5.42E-06	.	.	T	10.74	0.095	Uncertain_significance (Last reviewed: Oct 22, 2018)	642946	DICER1-related_pleuropulmonary_blastoma_cancer_predisposition_syndrome	criteria_provided, single_submitter
14	95129546	T	G	DICER1	NM_177438.2:c.660A>C	NP_803187.1:p.Glu220Asp	missense	LB	1.08E-05	.	.	T	23.2	0.151	Uncertain_significance (Last reviewed: Sep 6, 2018)	463923	Hereditary_cancer-predisposing_syndrome|DICER1-related_pleuropulmonary_blastoma_cancer_predisposition_syndrome	criteria_provided, multiple_submitters, no_conflicts
14	95129551	A	G	DICER1	NM_177438.2:c.655T>C	NP_803187.1:p.Leu219=	synonymous	LB	1.08E-05	1.84E-05	2.64E-05	.	.	.	Likely_benign (Last reviewed: Dec 31, 2019)	464741	Hereditary_cancer-predisposing_syndrome|not_provided	criteria_provided, multiple_submitters, no_conflicts
14	95129560	G	T	DICER1	NM_177438.2:c.646C>A	NP_803187.1:p.Pro216Thr	missense	LB	1.08E-05	1.84E-05	2.65E-05	T	23.4	0.283	Uncertain_significance (Last reviewed: Nov 18, 2019)	812202	Hereditary_cancer-predisposing_syndrome	criteria_provided, single_submitter
14	95129567	T	C	DICER1	NM_177438.2:c.639A>G	NP_803187.1:p.Lys213=	synonymous	LB	4.88E-05	.	8.81E-06	.	.	.	Likely_benign (Last reviewed: Dec 31, 2019)	242008	Hereditary_cancer-predisposing_syndrome|not_provided	criteria_provided, multiple_submitters, no_conflicts
14	95129612	T	C	DICER1	NM_177438.2:c.594A>G	NP_803187.1:p.Ser198=	synonymous	LB	5.42E-06	.	0	.	.	.	Likely_benign (Last reviewed: Nov 23, 2016)	242009	not_provided	criteria_provided, single_submitter
14	95129617	G	A	DICER1	NM_177438.2:c.589C>T	NP_803187.1:p.Pro197Ser	missense	LB	2.71E-05	.	8.86E-06	T	12.35	0.066	Uncertain_significance (Last reviewed: Oct 2, 2019)	464735	Hereditary_cancer-predisposing_syndrome|DICER1-related_pleuropulmonary_blastoma_cancer_predisposition_syndrome	criteria_provided, multiple_submitters, no_conflicts
14	95129623	T	G	DICER1	NM_177438.2:c.583A>C	NP_803187.1:p.Asn195His	missense	LB	1.63E-05	.	0	T	17.82	0.022	Uncertain_significance (Last reviewed: Dec 2, 2018)	400818	DICER1-related_pleuropulmonary_blastoma_cancer_predisposition_syndrome	criteria_provided, single_submitter
14	95130065	A	G	DICER1	NM_177438.2:c.566T>C	NP_803187.1:p.Ile189Thr	missense	LB	5.42E-06	.	.	T	24	0.708	.	.	.	.
14	95130072	G	T	DICER1	NM_177438.2:c.559C>A	NP_803187.1:p.Arg187=	synonymous	LB	5.42E-06	3.83E-05	3.53E-05	.	.	.	Likely_benign (Last reviewed: Dec 31, 2019)	242010	Hereditary_cancer-predisposing_syndrome|not_provided	criteria_provided, multiple_submitters, no_conflicts
14	95130077	G	C	DICER1	NM_177438.2:c.554C>G	NP_803187.1:p.Pro185Arg	missense	LB	5.42E-06	.	0	T	24.1	0.51	.	.	.	.
14	95130081	G	T	DICER1	NM_177438.2:c.550C>A	NP_803187.1:p.His184Asn	missense	LB	1.63E-05	.	.	T	19.66	0.275	.	.	.	.
14	95130082	G	C	DICER1	NM_177438.2:c.549C>G	NP_803187.1:p.Asp183Glu	missense	LB	5.42E-06	.	.	T	8.507	0.312	Uncertain_significance (Last reviewed: Oct 19, 2019)	399976	DICER1-related_pleuropulmonary_blastoma_cancer_predisposition_syndrome	criteria_provided, single_submitter

© 2021 Mirshahi UL et al. *JAMA Network Open.*

14	95130098	T	A	DICER1	NM_177438.2:c.533A>T	NP_803187.1:p.His178Leu	missense	pDeleterious	5.42E-06	1.89E-05	8.81E-06	D	23.3	0.829	Uncertain_significance (Last reviewed: Oct 15, 2019)	812213	Hereditary_cancer-predisposing_syndrome	criteria_provided, single_submitter

© 2021 Mirshahi UL et al. *JAMA Network Open.*

14	95130104	T	A	DICER1	NM_177438.2:c.527A>T	NP_803187.1:p.Glu176Val	missense	pDeleterious	5.42E-06	1.89E-05	8.81E-06	D	23.2	0.835	Uncertain_significance (Last reviewed: Aug 31, 2018)	528529	DICER1-related_pleuropulmonary_blastoma_cancer_predisposition_syndrome	criteria_provided, single_submitter
14	95130117	A	G	DICER1	NM_177438.2:c.514T>C	NP_803187.1:p.Leu172=	synonymous	LB	5.42E-06	.	.	.	.	.	Likely_benign (Last reviewed: Feb 9, 2018)	400184	Hereditary_cancer-predisposing_syndrome|not_provided	criteria_provided, multiple_submitters, no_conflicts
14	95130122	T	C	DICER1	NM_177438.2:c.509A>G	NP_803187.1:p.Asn170Ser	missense	LB	5.42E-06	.	.	T	16.59	0.164	.	.	.	.
14	95130134	A	G	DICER1	NM_177438.2:c.497T>C	NP_803187.1:p.Leu166Pro	missense	LB	2.71E-05	.	.	T	24.7	0.546	.	.	.	.
14	95130138	A	G	DICER1	NM_177438.2:c.493T>C	NP_803187.1:p.Ser165Pro	missense	LB	1.63E-05	.	.	T	17.76	0.153	Uncertain_significance (Last reviewed: Dec 15, 2019)	400819	Hereditary_cancer-predisposing_syndrome|DICER1-related_pleuropulmonary_blastoma_cancer_predisposition_syndrome	criteria_provided, multiple_submitters, no_conflicts
14	95130146	C	T	DICER1	NM_177438.2:c.485G>A	NP_803187.1:p.Gly162Asp	missense	LB	0.0003522	9.48E-05	0.0001	T	17.49	0.153	Benign/Likely_benign (Last reviewed: Dec 31, 2019)	242011	Hereditary_cancer-predisposing_syndrome|not_specified|not_provided	criteria_provided, multiple_submitters, no_conflicts
14	95131564	T	G	DICER1	NM_177438.2:c.383A>C	NP_803187.1:p.Glu128Ala	missense	LB	3.25E-05	.	.	T	17.89	0.224	Uncertain_significance (Last reviewed: May 4, 2019)	566802	Hereditary_cancer-predisposing_syndrome|DICER1-related_pleuropulmonary_blastoma_cancer_predisposition_syndrome	criteria_provided, multiple_submitters, no_conflicts
14	95131566	T	TA	DICER1	NM_177438.2:c.381delinsTA	NP_803187.1:p.Glu128Argfs	frameshift	pLOF	1.08E-05	.	.	.	.	.	.	.	.	.
14	95131568	G	C	DICER1	NM_177438.2:c.379C>G	NP_803187.1:p.Leu127Val	missense	LB	5.42E-06	0.0002	0.0001	T	11.28	0.056	Uncertain_significance (Last reviewed: Dec 12, 2019)	464738	Hereditary_cancer-predisposing_syndrome|DICER1-related_pleuropulmonary_blastoma_cancer_predisposition_syndrome	criteria_provided, multiple_submitters, no_conflicts
14	95131581	C	T	DICER1	NM_177438.2:c.366G>A	NP_803187.1:p.Gly122=	synonymous	LB	0.0002438	0.0003	0.0003	.	.	.	Likely_benign (Last reviewed: Dec 31, 2019)	222415	Hereditary_cancer-predisposing_syndrome|not_specified|not_provided	criteria_provided, multiple_submitters, no_conflicts
14	95131588	T	A	DICER1	NM_177438.2:c.359A>T	NP_803187.1:p.Lys120Met	missense	LB	2.71E-05	.	.	T	28.7	0.405	Uncertain_significance (Last reviewed: Apr 10, 2019)	400191	Goiter,_multinodular_1,_with_or_without_Sertoli-Leydig_cell_tumors|Hereditary_cancer-predisposing_syndrome|Rhabdomyosarcoma,_embryonal,_2|DICER1-related_pleuropulmonary_blastoma_cancer_predisposition_syndrome	criteria_provided, multiple_submitters, no_conflicts
14	95131627	G	A	DICER1	NM_177438.2:c.320C>T	NP_803187.1:p.Ala107Val	missense	LB	5.42E-06	.	.	T	21.7	0.153	Uncertain_significance (Last reviewed: Feb 28, 2019)	400504	Hereditary_cancer-predisposing_syndrome|DICER1-related_pleuropulmonary_blastoma_cancer_predisposition_syndrome	criteria_provided, multiple_submitters, no_conflicts
14	95132523	A	C	DICER1	NM_177438.2:c.299T>G	NP_803187.1:p.Val100Gly	missense	pDeleterious	1.08E-05	.	.	D	26.4	0.723	Uncertain_significance (Last reviewed: Aug 6, 2018)	642960	DICER1-related_pleuropulmonary_blastoma_cancer_predisposition_syndrome|not_provided	criteria_provided, multiple_submitters, no_conflicts
14	95132534	C	T	DICER1	NM_177438.2:c.288G>A	NP_803187.1:p.Thr96=	synonymous	LB	5.42E-06	.	0	.	.	.	Likely_benign (Last reviewed: Dec 20, 2018)	754251	not_provided	criteria_provided, single_submitter
14	95132535	G	A	DICER1	NM_177438.2:c.287C>T	NP_803187.1:p.Thr96Met	missense	LB	5.42E-06	.	0	T	29.9	0.275	.	.	.	.
14	95132544	C	T	DICER1	NM_177438.2:c.278G>A	NP_803187.1:p.Gly93Glu	missense	LB	3.25E-05	7.36E-05	7.03E-05	T	23.7	0.391	Uncertain_significance (Last reviewed: Nov 25, 2019)	400198	Hereditary_cancer-predisposing_syndrome|DICER1-related_pleuropulmonary_blastoma_cancer_predisposition_syndrome	criteria_provided, multiple_submitters, no_conflicts
14	95132546	A	G	DICER1	NM_177438.2:c.276T>C	NP_803187.1:p.Asn92=	synonymous	LB	2.71E-05	.	0	.	.	.	Likely_benign (Last reviewed: Dec 31, 2019)	400826	Hereditary_cancer-predisposing_syndrome|not_provided	criteria_provided, multiple_submitters, no_conflicts
14	95132550	C	T	DICER1	NM_177438.2:c.272G>A	NP_803187.1:p.Arg91Lys	missense	LB	7.58E-05	.	8.79E-06	T	4.02	0.033	Uncertain_significance (Last reviewed: Sep 20, 2019)	242016	Hereditary_cancer-predisposing_syndrome|DICER1-related_pleuropulmonary_blastoma_cancer_predisposition_syndrome	criteria_provided, multiple_submitters, no_conflicts
14	95132552	G	A	DICER1	NM_177438.2:c.270C>T	NP_803187.1:p.Ser90=	synonymous	LB	1.08E-05	1.84E-05	3.52E-05	.	.	.	.	.	.	.
14	95132566	T	C	DICER1	NM_177438.2:c.256A>G	NP_803187.1:p.Arg86Gly	missense	LB	5.42E-06	.	.	T	18.59	0.299	Uncertain_significance (Last reviewed: jul 19, 2017)	477205	Hereditary_cancer-predisposing_syndrome	criteria_provided, single_submitter
14	95132567	G	C	DICER1	NM_177438.2:c.255C>G	NP_803187.1:p.Ile85Met	missense	LB	5.42E-06	.	0	T	18.84	0.656	Conflicting_interpretations_of_pathogenicity (Last reviewed: Dec 31, 2019)	242017	Hereditary_cancer-predisposing_syndrome|not_provided	criteria_provided, conflicting_interpretations
14	95132574	T	C	DICER1	NM_177438.2:c.248A>G	NP_803187.1:p.Tyr83Cys	missense	LB	7.58E-05	3.68E-05	2.64E-05	T	23.7	0.179	Uncertain_significance (Last reviewed: Dec 30, 2019)	242018	Hereditary_cancer-predisposing_syndrome|DICER1-related_pleuropulmonary_blastoma_cancer_predisposition_syndrome	criteria_provided, multiple_submitters, no_conflicts
14	95132594	T	C	DICER1	NM_177438.2:c.228A>G	NP_803187.1:p.Leu76=	synonymous	LB	1.08E-05	.	.	.	.	.	Likely_benign (Last reviewed: Oct 31, 2018)	693632	Hereditary_cancer-predisposing_syndrome|not_provided	criteria_provided, multiple_submitters, no_conflicts
14	95132597	T	C	DICER1	NM_177438.2:c.225A>G	NP_803187.1:p.Val75=	synonymous	LB	2.71E-05	1.84E-05	8.79E-06	.	.	.	Likely_benign (Last reviewed: Dec 31, 2019)	400514	Hereditary_cancer-predisposing_syndrome|not_provided	criteria_provided, multiple_submitters, no_conflicts
14	95132599	C	G	DICER1	NM_177438.2:c.223G>C	NP_803187.1:p.Val75Leu	missense	LB	5.42E-06	.	.	T	22.8	0.255	.	.	.	.
14	95132624	A	G	DICER1	NM_177438.2:c.198T>C	NP_803187.1:p.Thr66=	synonymous	LB	1.08E-05	.	.	.	.	.	.	.	.	.
14	95132625	G	C	DICER1	NM_177438.2:c.197C>G	NP_803187.1:p.Thr66Ser	missense	pDeleterious	2.17E-05	.	.	D	13.03	0.299	.	.	.	.
14	95132626	T	C	DICER1	NM_177438.2:c.196A>G	NP_803187.1:p.Thr66Ala	missense	pDeleterious	5.42E-06	.	.	D	11.45	0.446	Uncertain_significance (Last reviewed: Mar 7, 2019)	528581	DICER1-related_pleuropulmonary_blastoma_cancer_predisposition_syndrome	criteria_provided, single_submitter
14	95132638	C	T	DICER1	NM_177438.2:c.184G>A	NP_803187.1:p.Val62Ile	missense	LB	2.71E-05	.	0	T	23.4	0.129	Uncertain_significance (Last reviewed: Sep 8, 2019)	528549	Hereditary_cancer-predisposing_syndrome|DICER1-related_pleuropulmonary_blastoma_cancer_predisposition_syndrome	criteria_provided, multiple_submitters, no_conflicts
14	95132643	G	A	DICER1	NM_177438.2:c.179C>T	NP_803187.1:p.Thr60Ile	missense	LB	7.04E-05	0.0001	0.0001	T	15.46	0.32	Uncertain_significance (Last reviewed: Dec 21, 2018)	137707	Hereditary_cancer-predisposing_syndrome|DICER1-related_pleuropulmonary_blastoma_cancer_predisposition_syndrome|not_specified	criteria_provided, multiple_submitters, no_conflicts
14	95133330	C	T	DICER1	NM_177438.2:c.129G>A	NP_803187.1:p.Thr43=	synonymous	LB	1.63E-05	.	.	.	.	.	Likely_benign (Last reviewed: Dec 31, 2019)	464773	Hereditary_cancer-predisposing_syndrome|DICER1-related_pleuropulmonary_blastoma_cancer_predisposition_syndrome	criteria_provided, multiple_submitters, no_conflicts

© 2021 Mirshahi UL et al. *JAMA Network Open.*

14	95133331	G	A	DICER1	NM_177438.2:c.128C>T	NP_803187.1:p.Thr43Met	missense	LB	1.63E-05	5.54E-05	3.59E-05	T	27.6	0.429	Uncertain_significance (Last reviewed: Dec 25, 2019)	400202	Hereditary_cancer-predisposing_syndrome|DICER1-related_pleuropulmonary_blastoma_cancer_predisposition_syndrome	criteria_provided, multiple_submitters, no_conflicts
14	95133333	A	G	DICER1	NM_177438.2:c.126T>C	NP_803187.1:p.Tyr42=	synonymous	LB	1.08E-05	1.85E-05	.	.	.	.	Likely benign (Last reviewed: Dec 31, 2019)	770017	Hereditary cancer-predisposing_syndrome|not_provided	criteria provided, multiple submitters, no_conflicts
14	95133339	G	A	DICER1	NM_177438.2:c.120C>T	NP_803187.1:p.Asn40=	synonymous	LB	5.42E-06	.	.	.	.	.	.	.	.	.
14	95133343	T	C	DICER1	NM_177438.2:c.116A>G	NP_803187.1:p.Asp39Gly	missense	LB	1.08E-05	.	0	T	23.9	0.396	.	.	.	.
14	95133345	A	G	DICER1	NM_177438.2:c.114T>C	NP_803187.1:p.His38=	synonymous	LB	5.42E-06	.	.	.	.	.	.	.	.	.
14	95133360	T	C	DICER1	NM_177438.2:c.99A>G	NP_803187.1:p.Gln33=	synonymous	LB	5.42E-06	.	.	.	.	.	Conflicting_interpretations_of_pathogenicity (Last reviewed: Jun 17, 2019)	400011	Hereditary_cancer-predisposing_syndrome|DICER1-related_pleuropulmonary_blastoma_cancer_predisposition_syndrome	criteria_provided, conflicting_interpretations
14	95133375	A	G	DICER1	NM_177438.2:c.84T>C	NP_803187.1:p.Phe28=	synonymous	LB	1.08E-05	.	.	.	.	.	Likely_benign (Last reviewed: Dec 31, 2019)	528588	not_provided	criteria_provided, single_submitter
14	95133382	G	A	DICER1	NM_177438.2:c.77C>T	NP_803187.1:p.Pro26Leu	missense	LB	5.42E-06	.	0	T	27.8	0.333	Uncertain_significance (Last reviewed: Dec 15, 2019)	464777	Medulloblastoma|Hereditary_cancer-predisposing syndrome|DICER1-related_pleuropulmonary_blastoma_cancer_predisposition_syndrome	criteria_provided, multiple_submitters, no_conflicts
14	95133389	T	C	DICER1	NM_177438.2:c.70A>G	NP_803187.1:p.Met24Val	missense	LB	5.42E-06	.	.	T	20.7	0.268	Uncertain_significance (Last reviewed: Nov 25, 2019)	242021	Hereditary_cancer-predisposing_syndrome|DICER1-related_pleuropulmonary_blastoma_cancer_predisposition_syndrome	criteria_provided, multiple_submitters, no_conflicts
14	95133400	G	A	DICER1	NM_177438.2:c.59C>T	NP_803187.1:p.Ala20Val	missense	LB	0.0007476	0.0007	0.0009	T	28.3	0.302	Conflicting_interpretations_of_pathogenicity (Last reviewed: Dec 31, 2019)	137703	Pleuropulmonary_blastoma|Hereditary_cancer-predisposing_syndrome|not_specified|not_provided	criteria_provided, conflicting_interpretations
14	95133401	C	T	DICER1	NM_177438.2:c.58G>A	NP_803187.1:p.Ala20Thr	missense	LB	5.42E-06	.	.	T	29.2	0.276	Uncertain_significance (Last reviewed: Dec 26, 2018)	528553	Hereditary_cancer-predisposing_syndrome|DICER1-related_pleuropulmonary_blastoma_cancer_predisposition_syndrome	criteria_provided, multiple_submitters, no_conflicts
14	95133434	G	A	DICER1	NM_177438.2:c.25C>T	NP_803187.1:p.Leu9Phe	missense	LB	3.25E-05	.	0	T	24	0.281	Uncertain_significance (Last reviewed: Mar 27, 2019)	528942	Hereditary_cancer-predisposing_syndrome|DICER1-related_pleuropulmonary_blastoma_cancer_predisposition_syndrome	criteria_provided, multiple_submitters, no_conflicts
14	95133437	G	T	DICER1	NM_177438.2:c.22C>A	NP_803187.1:p.Pro8Thr	missense	LB	5.42E-06	.	.	T	8.093	0.082	.	.	.	.
14	95133441	C	T	DICER1	NM_177438.2:c.18G>A	NP_803187.1:p.Leu6=	synonymous	LB	5.42E-06	2.18E-05	9.04E-06	.	.	.	Likely_benign(Last reviewed: Dec 31, 2019)	693633	not_provided	criteria_provided, single_submitter
14	95133448	G	C	DICER1	NM_177438.2:c.11C>G	NP_803187.1:p.Pro4Arg	missense	LB	1.08E-05	.	.	T	23.8	0.196	.	.	.	.
14	95133457	A	G	DICER1	NM_177438.2:c.2T>C	NP_803187.1:p.Met1?	initiator_codon	pLOF	2.71E-05	.	9.16E-06	T	24.1	0.245	Uncertain_significance (Last reviewed: Nov 21, 2019)	242022	Hereditary_cancer-predisposing_syndrome|DICER1-related_pleuropulmonary_blastoma_cancer_predisposition_syndrome	criteria_provided, multiple_submitters, no_conflicts
14	95133505	C	T	DICER1	NM_177438.2:c.-45-2A>G		splice_acceptor	pLOF	5.42E-06	.	.	.	.	.	.	.	.	.

© 2021 Mirshahi UL et al. *JAMA Network Open.*

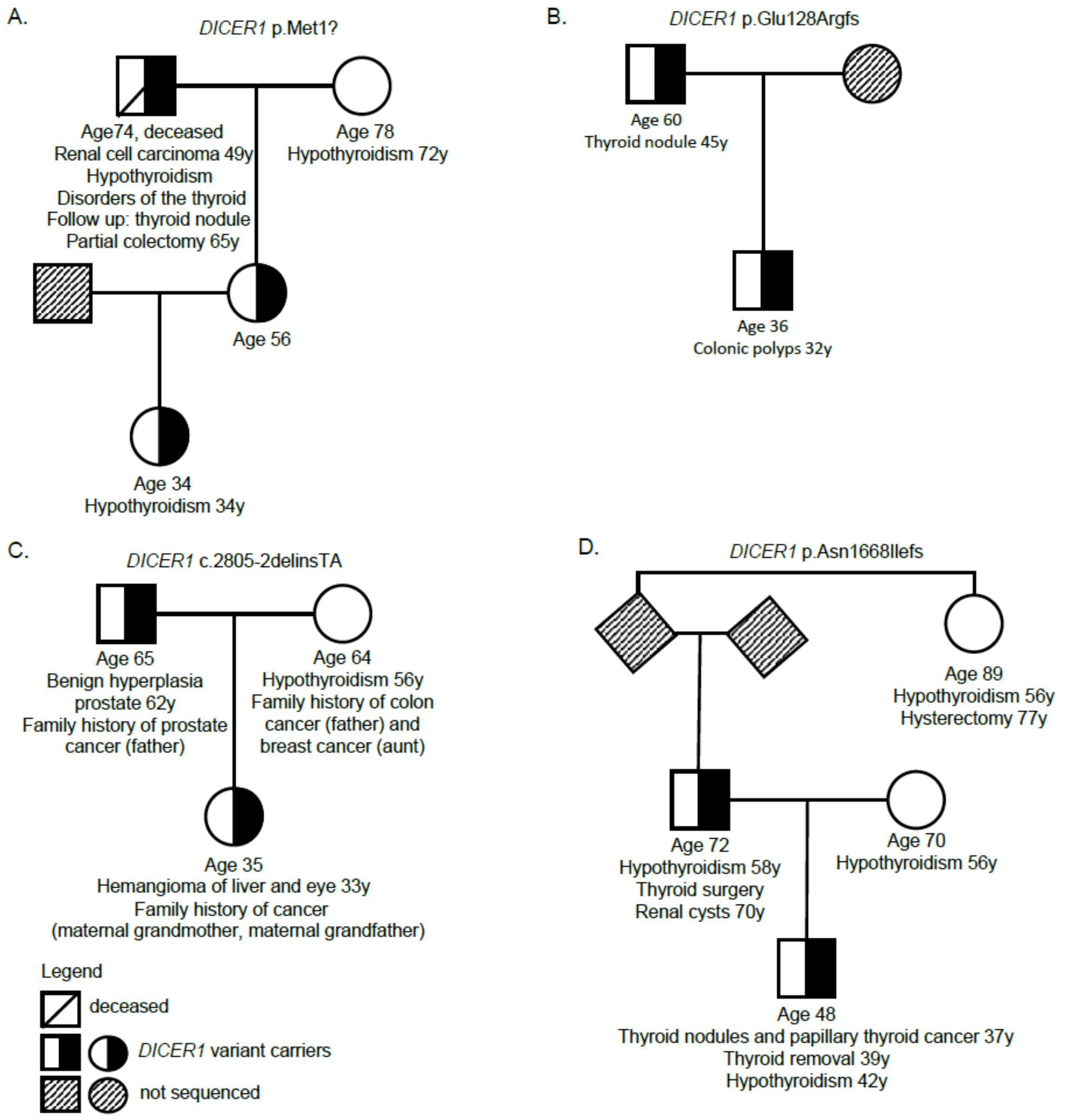

eFigure 1. Inferred pedigrees of carriers of *DICER1* putative loss-of-function variation

© 2021 Mirshahi UL et al. *JAMA Network Open.*

eTable 4. Demographic characteristics of 25 subjects with putative loss-of-function or hotspot *DICER1* variation in the Geisinger DiscovEHR study.

Variable	Values
Number of individuals	25
Race, %	
White	96
African American/Black	4
Ethnicity	
Non-Hispanic	96
Unknown	4
Sex, % female	72
Number deceased (%)	2 (8)
Current age, years[a]	
Mean	57
Median	57
Range	2 – 89
Number of clinical encounters	
Mean	68
Median	43
Range	6-249
Years of clinical encounters	
Mean	11
Median	14
Range	2 - 18
Smoking status, %	
Current smoker	48

[a]Non-deceased subjects

© 2021 Mirshahi UL et al. *JAMA Network Open.*

eTable 5. **Association of germline *DICER1* variants and thyroid phenotypes stratified by pathogenicity.** Odds ratios and p-values for thyroid traits for individuals from pLOFs, pDeleterious, VUS, and LB compared to non-carriers are calculated using Fisher's exact test. The total number of individuals (N) who were carriers and non-carriers of each *DICER1* variant class is noted under the classification group column header. Non-carriers were matched by sex, ethnicity, and smoking-status.

Group<br>N carriers | N non-carriers	pLOF & Hotspot<br>25 | 7550	pDeleterious<br>84 | 23,351	VUS<br>27 | 9268	Likely Benign<br>2289 | 80,491
Goiters				
Uni-nodular	2.7 (0.8 – 8.4)	1.4 (0.6 – 3.4)	1.1 (0.1 – 6.2)	0.8 (0.6 – 1.0)
Multi-nodular	0	0.4 (0.04 – 2.4)	1.1 (0.1 – 6.2)	1.0 (0.8 – 1.3)
Any	1.8 (0.7 – 4.9)	1.3 (0.7 -2.5)	0.9 (0.2 – 3.7)	0.9 (0.8 – 1.0)
Hypothyroidism	2.3 (1.1 – 5.1)	1.0 (0.6 – 1.7)	3.3 (1.6 – 7.2)[a]	0.7 (0.6 – 0.9)[a]
Hyperthyroidism	3.7 (0.9 – 13.6)	0.7 (0.1 – 4.2)	0	1.0 (0.8 – 1.4)
Thyroidectomy	6.0 (2.2 – 16.3)[a]	1.0 (0.2 – 3.5)	1.6 (0.1 – 9.3)	0.8 (0.6 – 1.1)
Malignancies				
Thyroid	9.2 (2.1 – 34.7)	1.7 (0.2 – 9.2)	0.9 (0.2 – 3.7)	0.9 (0.8 – 1.0)
Any malignancy	1.2 (0.4 – 3.4)	1.1 (0.6 – 2.0)	0.5 (0.1 – 2.1)	0.9 (0.8 – 1.1)

[a]denotes p<0.008, considered statistically significant with Bonferroni correction for multiple testing.
Abbreviations- pLOFs, putative loss of function; pDeleterious, predicted deleterious; VUS, variant of unknown significance; OR, odds ratio; CI, confidence intervals

© 2021 Mirshahi UL et al. *JAMA Network Open.*

eTable 6. Germline and somatic *DICER1* variants in cancers of individuals with germline *DICER1* predicted deleterious variants

Germline *DICER1* Variant	Malignancy	Age at biopsy (decade)	Sex	Ethnicity	Germline allele frequency[a] % (alt/total reads)	Somatic allele frequency[a] %
Thr66Ser	Multiple myeloma	40s	Male	White	63 (25/40)	Insufficient DNA
Val100Gly	Infiltrating duct carcinoma, NOS	60s	Female	White	46 (54/118)	Insufficient DNA
Thr806Met	"History of thyroid cancer" by ICD9 code; no pathology records in registry	60s	Male	White	40 (17/43)	Insufficient DNA
Thr806Met	Sigmoid colon, Adenocarcinoma	60s	Male	White	39 (14/36)	No hotspot
Thr806Met	Breast, infiltrating duct carcinoma	60s	Female	White	48 (45/94)	Insufficient DNA
Thr806Met	Intrahepatic bile duct, adenocarcinoma	50s	Male	White	52 (16/31)	Insufficient DNA
Leu881Val	Bone marrow, multiple myeloma	70s	Male	White	54 (61/113)	Insufficient DNA
	Prostate gland, adenocarcinoma	50s				Insufficient DNA
Ser1160Tyr	Lung, adenocarcinoma	80s	Female	Unknown	50 (66/132)	Insufficient DNA
Tyr1345Cys	Intraductal carcinoma	60s	Female	White	46 (19/41)	Insufficient DNA
Gly1364Ala	Testis, Seminoma	40s	Male	White	38 (28/74)	Gly1809Arg 1.5
Gly1364Ala	Thyroid gland, Papillary adenocarcinoma	30s	Female	White	50 (58/115)	Insufficient DNA
Trp1481Arg	Pancreas, Malignant Carcinoid tumor	60s	Female	White	39 (30/77)	Insufficient DNA

[a]Allele frequency determined as % alternate reads/total reads. Abbreviations: NOS: not otherwise specified; Insufficient DNA, DNA of insufficient quality for sequencing; alt, alternate variant

© 2021 Mirshahi UL et al. *JAMA Network Open.*

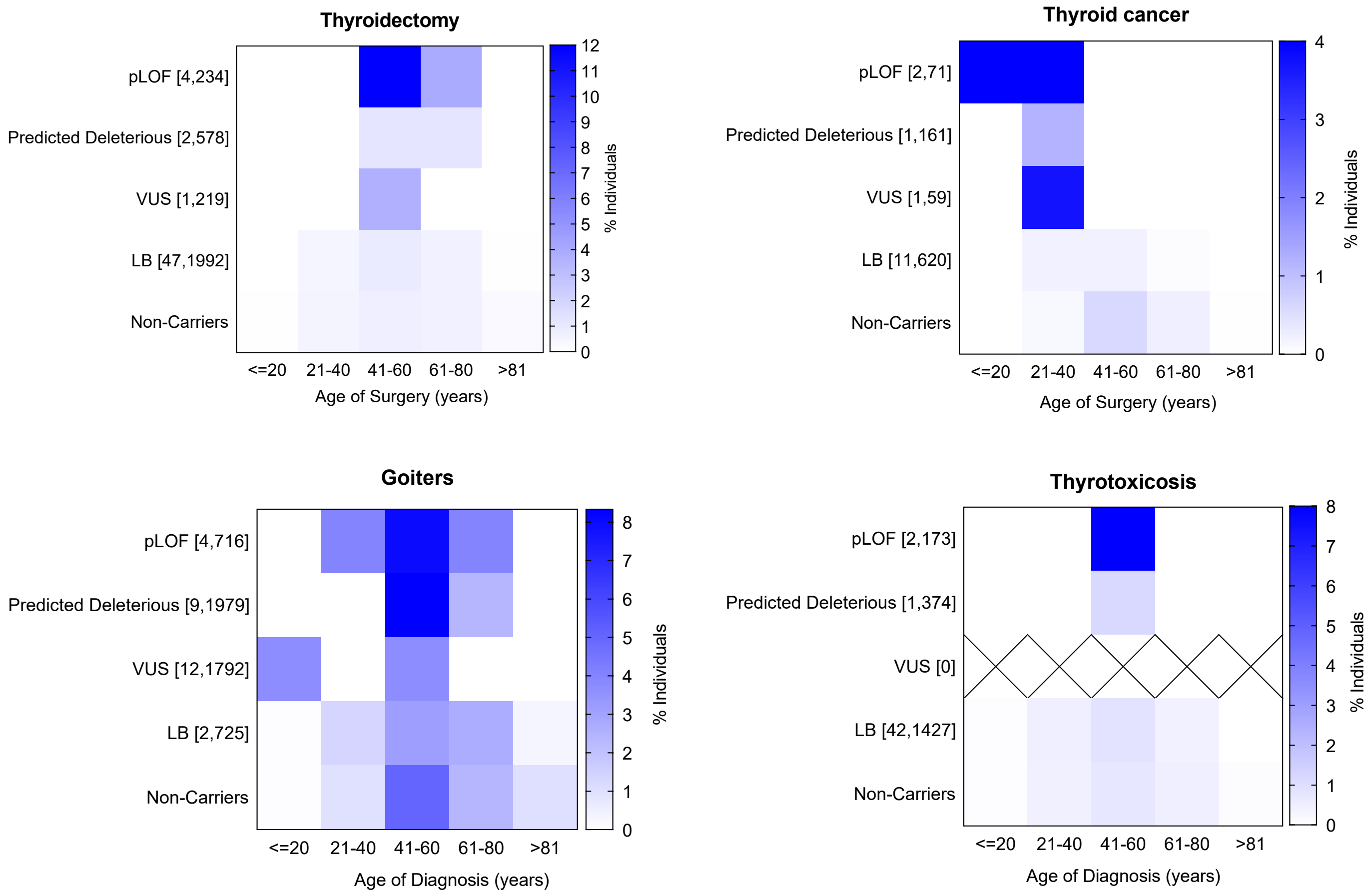

eFigure 2. Age of Diagnosis and Frequency of Diagnosis for Thyroid Conditions in Carriers and Non-Carriers (NC) of *DICER1* Variants. The frequency of carriers and non-carriers for *DICER1* groups (left y-axis) with each thyroid clinical trait expressed as % cases/group were separated per two decades based on the first age of diagnosis in EHR. Heatmaps of age-at-diagnosis (x-axis) and frequency in different age ranges (right y-axis) are shown. Darker shade of blue indicates higher frequency. The numbers in brackets next to each variant classification group indicate "n cases" for carriers or matched non-carriers. The "X" symbol (panel D) means no observations. The total number of individuals in each group is shown in Figure 1. pLOF: predicted loss of function, VUS: variant of uncertain significance; LB: likely benign

© 2021 Mirshahi UL et al. *JAMA Network Open.*
